# New Cinnamamide and Derivatives as New Larvicides against *Aedes aegypti* Vector Larvae: Facile Synthesis, *In Silico* Study, *In Vitro* Noncytotoxicity, and Nontoxicity against Zebrafish

**DOI:** 10.1021/acsomega.5c08177

**Published:** 2025-11-23

**Authors:** Adrielle Firmino da Silva, Saraliny Bezerra França, Erick Gabriel Alves Ferreira, Emiliano de Oliveira Barreto, Jeniffer Mclaine Duarte de Freitas, Johnnatan Duarte de Freitas, Edeildo Ferreira da Silva Júnior, Ana Catarina Rezende Leite, Pedro Correia Gomes dos Santos, Rafael David Souto de Azevedo, Josefa Gerlane da Silva, Dimas José da Paz Lima

**Affiliations:** † Institute of Chemistry and Biotechnology, 28112 Federal University of Alagoas, AC. Simões campus, Lourival Melo Mota Avenue, Maceió, Alagoas 57072-970, Brazil; ‡ Institute of Biological and Health Science, Federal University of Alagoas, AC. Simões campus, Lourival Melo Mota Avenue, Maceió, Alagoas 57072-970, Brazil; § Federal Institute of Alagoas, Rua Mizael Domingues, 530, Maceió, Alagoas 57020-600, Brazil; ∥ 502001 University of Pernambuco − UPE, Campus Garanhuns, São José, Garanhuns, Pernambuco 55294-902, Brazil; ⊥ Institute Keizo Asami, Federal University of Pernambuco, Av. Prof. Moraes Rego 1235, Cidade Universitária, Recife, State of Pernambuco 50670-901, Brazil

## Abstract

Every year, millions of people are infected by arboviruses, such as Dengue, a neglected disease that mainly affects regions with lower socioeconomic development. One of the main strategies to combat these diseases is population control of mosquito vectors, such as *Aedes aegypti*. Chemical control is an effective approach to reducing the population of these vectors; however, challenges such as toxicity and increasing resistance in populations make it necessary to search for new bioactive and selective substances continuously. In this sense, this study presents the synthesis and evaluation of the larvicidal activity of cinnamamides derived from cinnamic acid against the larvae of the *A. aegypti* mosquito. Given the increase in resistance to traditional larvicides, this research aims to offer safer and more effective alternatives for vector control. In this study, we synthesized 21 cinnamamides featuring scaffolds with and without electron-withdrawing substituents as well as variations in the amide side chain with aliphatic and aromatic groups (the latter containing a −CH_2_– spacer). The compounds were obtained via Steglich coupling or acid chloride formation followed by amidation and characterized by ^1^H NMR, ^13^C DEPTQ, and HRMS (for novel molecules). Larvicidal assays against fourth-instar *A. aegypti* revealed two active compounds: (*E*)-*N*-allyl-3-(4-chlorophenyl)­acrylamide (**AF03**, LC_50_ = 41.6 μg/mL) and (*E*)-*N*-allyl-3-(4-(trifluoromethoxy)­phenyl)­acrylamide (**AF18**, LC_50_ = 45.4 μg/mL). Cytotoxicity tests indicated that both molecules were nontoxic to A549 cells (5–100 μg/mL). **AF03** was further evaluated in chicken erythrocytes, showing no effect on oxygen transport, and in zebrafish, where it caused mild irritation but no mortality. Enzymatic assays showed that **AF03** increased acetylcholinesterase (AChE) activity in the zebrafish brain, slightly elevated catalase (CAT) activity in the liver and heart (similar to solvent effects), and increased superoxide dismutase (SOD) activity in the brain but not in the heart. Morphological analysis suggested potential involvement of receptor, transport, or digestive proteins as biological targets. Molecular docking identified sterol carrier protein-2 (AeSCP-2, PDB: 2ksi) and kynurenine aminotransferase (AeKAT, PDB: 1yiy) as promising targets, with AeSCP-2 selected for molecular dynamics simulations. MD results confirmed a stable and specific interaction between **AF03** and AeSCP-2, supported by the global structural stability of the protein and the low RMSD values of the ligand. This study suggests that cinnamamides may lead to the development of new, effective larvicidal agents with a low environmental impact.

## Graphical abstract

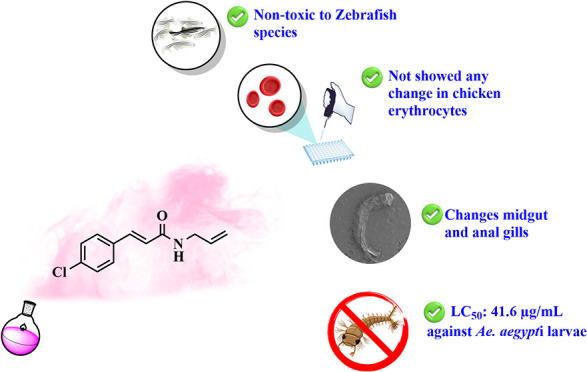

## 1 Introduction

Diseases caused by arboviruses represent a major global public health challenge with a wide geographical distribution, particularly in tropical regions. This is largely due to highly efficient vectors such as the *Aedes aegypti* mosquito, which is responsible for the transmission of emerging and re-emerging arboviruses, including Zika (ZIKV), Chikungunya (CHIKV), and Dengue (DENV).^1–3^ Between January and July 2025, more than 4 million cases and over 3.000 deaths were reported to the World Health Organization (WHO) across 97 countries.^4^ Most of these cases occurred in regions monitored by the WHO and the Pan American Health Organization (PAHO), which documented over 11 million notifications in 2024 alone. Within PAHO, the cumulative number of deaths from Dengue has surpassed 6.000, corresponding to a case fatality rate (CFR) of 0.057%.^5^ The economic burden of Dengue management is considerable, driving a continuous increase in healthcare expenditures due to the rising incidence of the disease.^6^

Although a vaccine has already been developed for the Dengue virus (Qdenga, CYD-TDV: Dengvaxia), its use remains restricted.^7^ Furthermore, no widely available and approved vaccines exist for ZIKV and CHIKV.^8^ Thus, vector control remains the primary method of preventing these diseases.^9^ Other strategies include habitat management and biological and chemical control. With a focus on chemical control, the main larvicides used belong to the organochlorine and pyrethroid groups, which act on ion channels.^10^ In addition, carbamates and organophosphates are widely used, acting as acetylcholinesterase inhibitors.^11^

Temephos, an organophosphate larvicide, was widely used to control *A. aegypti* and, for many years, was the only larvicide approved by the WHO for use in potable water.^12^ However, since the late 1990s, several studies have indicated an increase in the resistance of this vector to temephos, compromising the effectiveness of this larvicide.^1,13–16^ In addition, studies have shown the toxicity and potential carcinogenic effects of substances on nontarget organisms, including humans and aquatic fauna.^13^ Nowadays, the larvicides available are based on microbial agents such as spinosad, insect growth regulators (IGRs) such as pyriproxyfen, and other botanical origins.^17^ Regarding pyriproxyfen, studies have already reported its toxicity to nontarget species.^18^ This underscores the necessity of continuously developing new substances to control this vector. Addressing this challenge requires the development of effective, safe, and environmentally sustainable larvicides.

In this context, cinnamic acids and their derivatives, both natural and synthetic, have been widely studied for their larvicidal action against the larvae of the *A. aegypti* vector. Among these compounds, acid, ester, and aldehyde derivatives stand out, as they have documented larvicidal activity.^19–26^ However, the literature does not report on the larvicidal action of amides derived from these acids on the vector in question.

Cinnamamides, amides derived from cinnamic acid, have versatile structures that have been investigated in pharmaceuticals, agriculture, and biological products. Several studies have reported their leishmanicidal,^27^ antimicrobial,^28^ antiproliferative, and antimetastatic activity,^29^ as well as their anti-inflammatory,^30^ antitrypanosomal,^31^ antidiabetic,^32^ anticancer,^33^ antituberculosis,^34^ and antimalarial properties.^35^ In addition, various investigations into these derivatives have been reported in the field of pesticides, such as their herbicidal,^36^ fungicidal,^37,38^ nematicidal,^39,40^ insecticidal,^41^ and larvicidal activity against the larvae of the *Aedes albopictus* vector.^42^ Investigating cinnamamides as potential larvicides addresses a critical need for effective and environmentally safe strategies to control arbovirus transmission.

Previous studies by this group have demonstrated the excellent activity of cinnamic esters, with ethyl *p*-chlorocinnamate standing out, exhibiting an LC_50_ of 8.3 μg/mL.^43^ Structure–activity relationship analyses indicate that the presence of a chlorine atom confers higher larvicidal activity compared to other substituents (*F* < Br < Cl), possibly due to polarity and interactions with the molecular target. Considering these results, along with the report by Han et al.^42^ of a cinnamamide with an LC_50_ of 0.45 ppm against *A. albopictus*, and the investigation by Oliveira et al.^44^ on tryptamine-derived amides against *A. aegypti*, it was observed that an increase in the number of aliphatic carbons in the amide side chain enhances larvicidal potency, possibly related to hydrophobicity. Chlorinated derivatives showed up to ten times higher activity than nonchlorinated compounds.^44^

Based on these findings, this study investigated the larvicidal activity of cinnamamides with and without electron-withdrawing groups, containing both aliphatic and aromatic substituents. In this context, considering the low toxicity of substances derived from cinnamic acid and the unprecedented larvicidal action of cinnamamides against the larvae of the *A. aegypti* vector, this research set out to synthesize, characterize, and evaluate different cinnamic amides in terms of their larvicidal efficacy. Additionally, *in silico* studies and morphological analyses were performed to investigate the potential mechanism of action. Furthermore, *in vitro* toxicity assays and tests using the nontarget organism *Danio rerio* (zebrafish) were conducted to evaluate the toxic effects of the most active compound.

## 2 Experimental Section

### 2.1 Synthesis and Characterization of Cinnamic Amides

#### 2.1.1 Method A

Cinnamamides from **AF01** to **AF12** were synthesized by the Steglich reaction^45^ using cinnamic acids, primary amines, dicyclohexylcarbodiimide (DCC) as a coupling reagent, dimethylaminopyridine (DMAP) as a catalyst, and acetonitrile under anhydrous conditions and an inert atmosphere. The compounds were characterized by hydrogen nuclear magnetic resonance (^1^H NMR) and carbon nuclear magnetic resonance (^13^C NMR) using the DEPTQ technique, using Bruker equipment with frequencies of 600, 400, 150, and 100 MHz. The deuterated solvents used to prepare the samples were obtained commercially from Cambridge Isotope Laboratories and contained tetramethylsilane (TMS) as an internal standard. TopSpin 4.0.8 software (BRUKER) was used to analyze the ^1^H and ^13^C spectra. The multiplicities of the hydrogen nuclei energy absorption signals in the ^1^H NMR spectra are indicated according to the following convention: s (singlet), d (doublet), t (triplet), and m (multiplet). The chemical shifts were expressed in parts per million (scale δ) and the coupling constants in Hertz (Hz). All of the compounds’ infrared (IR) spectra, obtained using the Fourier Transform Infrared (FT-IR) spectroscopy technique, were obtained on a Shimadzu IRPrestige apparatus. Finally, the mass spectrometry analysis for the unpublished compounds was carried out on a Bruker model HCT Ultra ion trap mass spectrometer coupled to HPLC (mass range: 50–6000 m/z) (See the Supporting Information).

##### 2.1.1.1 (*E*)-3-(4-Chlorophenyl)-*N*-hexylacrylamide (**AF01**)

White solid, purity: 100%; yield: 54%; mp: 113–114 °C. ^1^H NMR (600 MHz, CDCl_3_, δ (ppm)): 7.56 (d, *J* = 16.0 Hz, 1H); 7.40 (d, *J* = 8.4 Hz, 2H); 7.30 (d, *J* = 8.4 Hz, 2H); 6.35 (d, *J* = 16.0 Hz, 1H); 3.37 (q, *J* = 6.8 Hz, 2H); 1.56 (q, *J* = 7.2 Hz, 2H); 1.3 (m, 6H); 0.85 (t, *J* = 6.8 Hz, 3H); 5.69 (s, 1H); ^13^C NMR (150 MHz, CDCl_3_, δ (ppm)): 165.50 (C=O); 135.42 (C–Cl); 133.46 (C); 139.47 (CH); 129.04 (CH); 121.43 (CH); 39.86 (CH_2_); 31.47 (CH_2_); 29.63 (CH_2_); 26.61 (CH_2_); 22.52 (CH_2_); 13.95 (CH_3_); FT-IR (cm^–1^): 3296 (vs N–H); 2927 (vs C–H and vas C–H); 2854 (vs CH_2_/CH_3_ and vas CH_2_/CH_3_); 1537–1614 (*v* C=C aromatic ring); 1320–1279 (vs C–O); 1708 (C=O); 1651 (*trans* double bond); 727 (long chain band); 821 (*p*-disubstituted ring).^46^

##### 2.1.1.2 (*E*)-3-(4-Chlorophenyl)-*N*-phenethylacrylamide (**AF02**)

White solid, purity: 100%; yield: 47%; mp: 147–148 °C. ^1^H NMR (600 MHz, CDCl_3_, δ (ppm)): 7.58 (d, *J* = 16.0 Hz, 1H); 7.40 (d, *J* = 8.2 Hz, 2H); 7.32 (t, *J* = 7.1 Hz, 4H); 7.25 (q, *J* = 6.9 Hz, 3H); 6.28 (d, *J* = 16.0 Hz, 1H); 5.67 (s, 1H); 3.67 (q, *J* = 6.6 Hz, 2H); 2.89 (t, *J* = 6.8 Hz, 2H); ^13^C NMR (150 MHz, CDCl_3_, δ (ppm)): 165.52 (C=O); 138.81 (C–Cl); 135.51 (C); 133.36 (C); 139.71 (CH); 128.71 (CH); 126.70 (CH); 121.20 (CH); 40.80 (CH_2_); 35.64 (CH_2_); FT-IR (cm^–1^): 3308 (vs N–H); 2930 (vs C–H and vas C–H); 2848 (vs CH_2_/CH_3_ and vas CH_2_/CH_3_); 1486–1615 (C=C of aromatic ring); 1326–1214 (vs C–O); 1651 (C=O); 972 (*trans* double bond); 820 (ring *p*-substituted).^47^

##### 2.1.1.3 (*E*)-*N*-Allyl-3-(4-chlorophenyl)­acrylamide (**AF03**)

White solid, purity: 98%; yield: 51%; mp: 117–119 °C. ^1^H NMR (600 MHz, CDCl_3_, δ (ppm)): 7.63 (d, *J* = 16.0 Hz, 1H); 7.45 (d, *J* = 8.4 Hz, 2H); 7.36 (d, *J* = 8.4 Hz, 2H); 6.38 (d, *J* = 16.0 Hz, 1H); 5.90 (m, 1H); 5.73 (s, 1H); 5.24 (dd, *J* = 1.2 and 1.1 Hz, 1H); 5.18 (dd, *J* = 0.9 and 0.9 Hz, 1H); 4.04 (t, *J* = 5.7 Hz, 2H); ^13^C NMR (150 MHz, CDCl_3_, δ (ppm)): 165.35 (C=O); 135.57 (C–Cl); 133.33 (C); 116.73 (CH_2_); 139.97 (CH); 134.02 (CH); 129.09 (CH); 128.94 (CH); 121.01 (CH); 42.19 (CH_2_); FT-IR (cm^–1^): 3257 (vs N–H); 2924 (vs C–H and vas C–H); 2842 (vs CH_2_ and vas CH_2_); 1657 (C=O); 1615–1491 (C=C aromatic ring); 1084 (vs C–O); 972 (*trans* double bond); 813 (*p*-displaced ring); HRMS (ESI) calcd for C_12_H_12_ClNO [M + H]^+^, 221.06812, found, 222.06812.

##### 2.1.1.4 *N*-Hexylcinnamamide (**AF04**)

Colorless oil, purity: 100%; yield: 72%. ^1^H NMR (600 MHz, CDCl_3_, δ (ppm)): 7.63 (d, *J* = 16.0 Hz, 1H); 7.49 (d, *J* = 7.0 Hz, 2H); 6.38 (d, *J* = 16.0 Hz, 1H); 7.35 (d, *J* = 7.0 Hz, 3H); 5.78 (s, 1H); 3.36 (q, *J* = 6.4 Hz, 2H); 1.57 (q, *J* = 7.6 Hz, 2H); 1.35 (m, 6H); 0.88 (t, *J* = 6.4 Hz, 3H); ^13^C NMR (150 MHz, CDCl_3_, δ (ppm)): 165.88 (C=O); 134.95 (C); 140.83 (CH); 128.78 (CH); 127.74 (CH); 120.85 (CH); 39.84 (CH_2_); 31.50 (CH_2_); 29.65 (CH_2_); 22.54 (CH_2_); 13.98 (CH_3_); FT-IR (cm^–1^): 3290 (vs N–H); 2994 (vs C–H and vas C–H); 2850 (vs CH_2_/CH_3_ and Vas CH_2_/CH_3_); 1450–1615 (C=C of aromatic ring); 1332–1219 (vs C–O); 1651 (C=O); 974 (*trans* double bond); 720 (long chain band); 690 (singly substituted ring).^48^

##### 2.1.1.5 *N*-Phenethylcinnamamide (**AF05**)

White solid, purity: 100%; yield: 64%; mp 121–122 °C. ^1^H NMR (600 MHz, CDCl_3_, δ (ppm)): 7.58 (d, *J* = 16.0 Hz, 1H); 7.46 (d, *J* = 5.4 Hz, 2H); 7.33 (q, *J* = 6.4 Hz, 5H); 7.23 (q, *J* = 7.1 Hz, 1H); 6.29 (d, *J* = 16.0 Hz, 1H); 5.67 (s, 1H); 3.63 (q, *J* = 6.6 Hz, 2H); 2.86 (t, *J* = 6.9 Hz, 2H); ^13^C NMR (150 MHz, CDCl_3_, δ (ppm)): 165.85 (C=O); 138.89 (C); 134.88 (C); 141.03 (CH); 129.63 (CH); 128.79 (CH); 128.69 (CH); 127.76 (CH); 126.56 (CH); 120.69 (CH); 40.79 (CH_2_); 35.69 (CH_2_); FT-IR (cm^–1^): 3296 (vs N–H); 2924 (vs C–H and vas C–H); 2854 (vs CH_2_–/CH_3_ and vas CH_2_/CH_3_); 1444–1610 (C=C aromatic ring); 1214–1196 (vs C–O); 1651 (C=O); 967 (*trans* double bond); 690 (singly substituted ring).^49^

##### 2.1.1.6 *N*-Allylcinnamamide (**AF06**)

White solid, purity: 98%; yield: 51%; mp 88–89 °C. ^1^H NMR (600 MHz, CDCl_3_, δ (ppm)): 7.64 (d, *J* = 16.0 Hz, 1H); 7.50 (d, *J* = 6.2 Hz, 2H); 7.36 (d, *J* = 6.5 Hz, 3H); 6.41 (d, *J* = 16.0 Hz, 1H); 5.88 (m, 1H); 5.76 (s, 1H); 5.23 (d, *J* = 17.1 Hz, 1H); 5.17 (d, *J* = 10.2 Hz, 1H); 4.03 (t, *J* = 5.4 Hz, 2H); ^13^C NMR (150 MHz, CDCl_3_, δ (ppm)): 165.70 (C=O); 134.81 (C); 116.65 (CH_2_); 141.31 (CH); 134.14 (CH); 129.68 (CH); 128.81 (CH); 127.78 (CH); 120.48 (CH); 42.2 (CH_2_); FT-IR (cm^–1^): 3279 (vs N–H); 3060 (vs C–H and vas C–H); 2913 (vs CH_2_ and vas CH_2_); 1651 (C=O); 1444–1621 (C=C aromatic ring); 1214 (vs C–O); 972 (*trans* double bond); 690 (single substitute ring).^50^

##### 2.1.1.7 (*E*)-3-(4-Chlorophenyl)-*N*-(4-fluorophenethyl)­acrylamide (**AF07**)

White solid, purity: 100%; yield: 55%; mp 153–154 °C. ^1^H NMR (600 MHz, CDCl_3_, δ (ppm)): 7.55 (d, *J* = 16.0 Hz, 1H); 7.40 (d, *J* = 8.2 Hz, 2H); 7.32 (d, *J* = 8.2 Hz, 2H); 7.17 (q, *J* = 5.6 Hz, 2H); 7.00 (t, *J* = 8.6 Hz, 2H); 6.32 (d, *J* = 16.0 Hz, 1H); 5.72 (s, 1H); 3.61 (q, *J* = 6.7 Hz, 2H); 2.85 (t, *J* = 6.9 Hz, 2H); ^13^C NMR (150 MHz, CDCl_3_, δ (ppm)): 165.56 (C=O); 162.54 (C–F); 135.57 (C–Cl); 134.44 (C); 133.29 (C); 139.85 (CH); 130.19 (CH); 129.07 (CH); 128.94 (CH); 121.06 (CH); 115.56 (CH); 115.42 (CH); 40.93 (CH_2_); 34.88 (CH_2_); FT-IR (cm^–1^): 3287 (vs N–H); 2927 (vs C–H and vas C–H); 2854 (vs CH_2_ and vas CH_2_); 1654 (C=O); 1614–1500 (C=C of aromatic ring); 1320 (vs C–O); 820 (*p*-disubstituted ring); HRMS (ESI) calcd for C_17_H_15_ClFNO [M + H]^+^, 305.15692, found, 305.15692.

##### 2.1.1.8 (*E*)-*N*-Benzyl-3-(4-chlorophenyl)­acrylamide (**AF08**)

White solid, purity: 98% yield: 51%; mp: 145–147 °C. ^1^H NMR (600 MHz, CDCl_3_, δ (ppm)): 7.62 (d, *J* = 16.0 Hz, 1H); 741 (d, *J* = 8.4 Hz, 2H); 7.30 (m, 7H); 6.37 (d, *J* = 16.0 Hz, 1H); 5.95 (s, 1H); 4.58 (d, *J* = 5.6 Hz, 2H); ^13^C NMR (150 MHz, CDCl_3_, δ (ppm)): 165.41 (C=O); 138.09 (C–Cl); 135.60 (C); 133.31 (C); 140.12 (CH); 129.09 (CH); 128.95 (CH); 128.79 (CH); 127.93 (CH); 127.66 (CH); 43.95 (CH_2_); FT-IR (cm^–1^): 3290 (vs N–H); 2919 (vs C–H and vas C–H); 2842 (vs CH_2_ and vas CH_2_); 1657 (C=O); 1486–1615 (C=C of aromatic ring); 1220 (vs C–O); 967 (*trans* double bond); 695 (monosubstituted ring); 813 (*p*-substituted ring).^46^

##### 2.1.1.9 (*E*)-3-(4-Chlorophenyl)-*N*-cyclohexylacrylamide (**AF09**)

White solid, purity: 100%; yield: 53%; mp 199–200 °C. ^1^H NMR (600 MHz, CDCl_3_, δ (ppm)): 7.52 (d, *J* = 16.0 Hz, 1H); 7.39 (d, *J* = 6.2 Hz, 2H); 7.30 (d, *J* = 6.5 Hz, 2H); 6.30 (d, *J* = 16.0 Hz, 1H); 5.51 (s, 1H); 3.88 (t, *J* = 5.4 Hz, 2H); 1.96 (d, *J* = 10.2 Hz, 1H); 1.72 (m, 4H); 1.37 (m, 2H); 1.16 (m, 2H) ^13^C NMR (150 MHz, CDCl_3_, δ (ppm)): 164.55 (C=O); 135.37 (C–Cl); 133.51 (C); 139.35 (CH); 129.04 (CH); 128.87 (CH); 121.75 (CH); 48.44 (CH); 33.22 (CH_2_); 25.55 (CH_2_); 24.83 (CH_2_); FT-IR (cm^–1^): 3279 (vs N–H); 3060 (vs C–H and vas C–H); 2913 (vs CH_2_ and vas CH_2_); 1651 (C=O); 1444–1621 (C=C aromatic ring); 1214 (vs C–O); 972 (double *trans*); 690 (single substitute ring).^51^

##### 2.1.1.10 *N*-(4-Fluorophenethyl)­cinnamamide (**AF10**)

White solid, purity: 99%; yield: 55%; mp 144–145 °C. ^1^H NMR (600 MHz, CDCl_3_, δ (ppm)): 7.44 (d, *J* = 16.0 Hz, 1H); 7.32 (d, *J* = 7.5 Hz, 2H); 7.15 (d, *J* = 5.9 Hz, 3H); 7.13 (q, *J* = 5.6 Hz, 2H); 6.97 (t, *J* = 8.6 Hz, 2H); 6.28 (d, *J* = 16.0 Hz, 1H); 5.62 (s, 1H); 3.58 (q, *J* = 6.6 Hz, 2H); 2.82 (t, *J* = 6.9 Hz, 2H); ^13^C NMR (150 MHz, CDCl_3_, δ (ppm)): 165.85 (C=O); 162.54 (C–F); 134.81 (C); 134.50 (C); 141.19 (CH); 130.20 (CH); 129.69 (CH); 128.80 (CH); 127.77 (CH); 120.52 (CH); 115.54 (CH); 115.40 (CH); 40.90 (CH_2_); 34.92 (CH_2_); FT-IR (cm^–1^): 3326 (vs N–H); 2924 (vs C–H and vas C–H); 2854 (vs CH_2_ and vas CH_2_); 1657 (C=O); 1615–1503 (C=C aromatic ring); 1220 (vs C–O); 967 (*trans* double bond); 825 (*p*-disubstituted ring). HRMS-ESI calcd for C_17_H_16_FNO [M + H]^+^, 270.12897, found, 270.12897.

##### 2.1.1.11 *N*-Benzylcinnamamide (**AF11**)

White solid, purity: 98%; yield: 55%; mp 100–101 °C. ^1^H NMR (600 MHz, CDCl_3_, δ (ppm)): 7.50 (d, *J* = 16.0 Hz, 1H); 7.48 (m, 2H); 7.40 (m, 8H); 6.40 (d, *J* = 16.0 Hz, 1H); 5.97 (s, 1H); 4.57 (d, *J* = 5.6 Hz, 2H); ^13^C NMR (150 MHz, CDCl_3_, δ (ppm)): 165.73 (C=O); 138.21 (C); 134.82 (C); 141.45 (CH); 129.72 (CH); 128.82 (CH); 128.77 (CH); 127.94 (CH); 127.80 (CH); 127.61 (CH); 120.45 (CH); 43.91 (CH_2_). FT-IR (cm^–1^): 3326 (vs N–H); 2924 (vs C–H and vas C–H); 2854 (vs CH_2_ and vas CH_2_); 1657 (C=O); 1550–1615 (C=C of aromatic ring); 1220 (vs C–O); 972 (*trans* double bond); 666 (monosubstituted ring); 825 (*p*-substituted ring).^52^

##### 2.1.1.12 *N*-Cyclohexylcinnamamide (**AF12**)

White solid, purity: 100%; yield: 51%; mp 178–179 °C. ^1^H NMR (600 MHz, CDCl_3_, δ (ppm)): 7.63 (d, *J* = 16.0 Hz, 1H); 7.49 (d, *J* = 6.5 Hz, 2H); 7.34 (m, 2H); 6.37 (d, *J* = 16.0 Hz, 1H); 5.60 (s, 1H); 3.91 (m, 1H); 1.99 (d, *J* = 9.8 Hz, 2H); 1.72 (m, 2H); 1.63 (d, *J* = 13.1, 1H); 1.37 (m, 2H); 1.18 (t, *J* = 5.6, 3H); ^13^C NMR (150 MHz, CDCl_3_, δ (ppm)): 164.91 (C=O); 135.01 (C); 140.69 (CH); 129.50 (CH); 128.76 (CH); 127.71 (CH); 121.20 (CH); 127.71 (CH); 121.20 (CH); 48.40 (CH); 33.25 (CH_2_); 25.57 (CH_2_); 24.85 (CH_2_); FT-IR (cm^–1^): 3266 (vs N–H); 2913 (vs C–H and vas C–H); 2848 (vs CH_2_ and Vas CH_2_); 1651 (C=O); 1545–1615 (C=C of aromatic ring); 1220 (vs C–O); 978 (*trans* double bond); 677 (singly substituted ring).^53^

#### 2.1.2 Method B

Cinnamamides from **AF13** to **AF21** were obtained by converting cinnamic acids to acyl chlorides for subsequent amidation reactions. This method used thionyl chloride, anhydrous dichloromethane, anhydrous dimethylformamide, and primary amines.^54^

##### 2.1.2.1 (*E*)-*N*-Allyl-3-(4-methoxyphenyl)­acrylamide (**AF13**)

White solid, purity: 97%; yield: 75%; mp 117–118 °C. ^1^H NMR (600 MHz, CDCl_3_, δ (ppm)): δ 3.85 (s, 3H); 4.03 (dd, *J* = 7.0 Hz, 2H); 5.17–5.27 (2H, 5.17 (dd, *J* = 16.0, 1.3 Hz); 5.27 (dd, *J* = 10.0, 1.3 Hz)); 5.89 (1H, ddt, *J* = 16.0 Hz, 10.0, 5.5 Hz, 1H); 6.28 (d, *J* = 15.5 Hz, 1H); 6.90 (d, *J* = 8.8 Hz, 2H); 7.46 (d, *J* = 8.8 Hz, 2H); 7.61 (d, *J* = 15.5 Hz); ^13^C NMR (150 MHz, CDCl_3_, δ (ppm)): 166.01 (C); 160.95 (C) 140.90 (CH); 134.29 (CH); 129.52 (CH); 129.32 (CH); 127.56 (C); 118.09 (CH); 116.52 (C); 114.28 (CH); 55.34 (CH_3_); 42.13 (CH_2_); FT-IR (cm^–1^): 3221 (vs N–H); 2346 (vs C–H and vas C–H); 1651 (v C=O); 1597–1497 (C=C aromatic ring); 1244 (vs C–O); 990 (*trans* double bond); 813 (*p*-displaced ring). HRMS (ESI) calcd for C_13_H_15_NO_2_ [M + H]^+^, 218.11758, found, 218.11758.

##### 2.1.2.2 (*E*)-*N*-Allyl-3-(4-bromophenyl)­acrylamide (**AF14**)

White solid, purity: 100%; yield: 95%; mp 150–151 °C. ^1^H NMR (600 MHz, CDCl_3_, δ (ppm)): δ 4.0 (dd, *J* = 7.0 Hz, 2H); 5.16–5.27 (2H, 5.16 (dd, *J* = 16.0, 1.3 Hz); 5.27 (dd, *J* = 10.0, 1.3 Hz)); 5.67 (s, 1H); 5.85 (1H, ddt, *J* = 16.0 Hz, 10.0, 5.5 Hz, 1H); 5.90 (d, *J* = 15.5 Hz); 7.36 (d, *J* = 8.8 Hz, 2H); 7.50 (d, *J* = 8.8 Hz, 2H); 7.63 (d, *J* = 15.5 Hz); ^13^C NMR (150 MHz, CDCl_3_, δ (ppm)): 165.38 (C); 133.73 (C); 140.05 (CH); 133.98 (CH); 132.05 (CH); 129.20 (CH); 123.85 (CH); 121.09 (CH); 116.75 (CH_2_) 42.20 (CH_2_); FT-IR (cm^–1^): 3272 (vs N–H); 1645 (v C=O); 1615–1527 (C=C aromatic ring); 1214 (vs C–O); 972 (*trans* double bond); 813 (*p*-displaced ring). HRMS (ESI) calcd for C_12_H_12_BrNO [M + H]^+^, 266.01740, found, 266.01740.

##### 2.1.2.3 (*E*)-*N*-Allyl-3-(4-cyanophenyl)­acrylamide (**AF15**)

White solid, purity: 100%; yield: 98%; mp: 159–164 °C. ^1^H NMR (600 MHz, CDCl_3_, δ (ppm)): δ 4.04 (tt, *J* = 1.5, 3.0 e 7.8 Hz, 2H); 5.19–5.29 (2H, 5.19 (dd, *J* = 10.0, 1.5 Hz, 1H); 5.29 (dd, *J* = 16.0, 1.5 Hz, 1H)); 5.76 (s, 1H); 5.87 (ddt, 5.7, 10, 16 Hz, 1H); 6.50 (d, *J* = 15.5 Hz, 1H); 7.57 (d, *J* = 7.5 Hz, 2H); 7.63 (d, *J* = 7.5 Hz, 2H); 7.67 (d, *J* = 15.5 Hz); ^13^C NMR (150 MHz, CDCl_3_, δ (ppm)): 165.05 (C); 139.66 (CH); 138.21 (C); 133.85 (CH); 131.12 (CH); 127.91 (CH); 125.23 (CH); 122.89 (CH); 116.87 (CH_2_); 42.25 (CH_2_); HRMS (ESI) calcd for C_13_H_12_N_2_O_1_ [M + H]^+^, 213.1019, found, 213.1019.

##### 2.1.2.4 (*E*)-*N*-Allyl-3-(4-fluorophenyl)­acrylamide (**AF16**)

White solid, purity: 100%; yield: 98%; mp: 159–164 °C. ^1^H NMR (600 MHz, CDCl_3_, δ (ppm)): δ 4.01 (tt, *J* = 1.5, 4.3 e 6.2 Hz, 2H); 5.15–5.27 (5.15 (dd, *J* = 10.0, 1.5 Hz, 1H); 5.27 (dd, *J* = 16.0, 1.5 Hz, 1H)); 5.82 (s, 1H); 5.85 (ddt, 5.7, 10, 16 Hz, 1H); 6.33 (d, *J* = 15.5 Hz, 1H); 7.03 (dd, *J* = 2.0, 8.6 Hz, 2H); 7.05 (dd, *J* = 2.0, 8.6 Hz, 2H); 7.46 (d, *J* = 15.5 Hz); ^13^C NMR (150 MHz, CDCl_3_, δ (ppm)): δ 42.81 (1 CH_2_); 115.82 (2 CH); 116.04 (1 CH_2_); 120.16 (1 CH); 129.54 (2 CH); 134.06 (1 CH); 140.08 (1 CH); 162.31 (C); 164.79 (C–F); 165.69 (C=O); FT-IR (cm^–1^): 3234 (vs N–H); 1662 (v C=O); 1607–1504 (C=C aromatic ring); 1205 (vs C–O); 968–919 (*trans* double bond); 827 (*p*-displaced ring). HRMS (ESI) calcd for C_12_H_12_FNO [M + H]^+^, 206.0976, found, 206.0976.

##### 2.1.2.5 (*E*)-*N*-Allyl-3-(4-(trifluoromethyl)­phenyl)­acrylamide (**AF17**)

White solid, purity: 98%; yield: 95%; mp 143–144 °C. ^1^H NMR: δ 4.0 (tt, *J* = 1.5, 2.3 e 7.2 Hz, 2H); 5.19–5.30 (5.19 (dd, *J* = 1.3, 10.0 Hz, 1H); 5.30 (dd, *J* = 17.0, 1.3 Hz, 1H); 5.71 (s, 1H); 5.87 (ddt, *J* = 5.7, 10.0, 17.0 Hz, 2H)); 6.48 (d, *J* = 15.6 Hz, 1H); 7.60 (d, *J* = 7.5 Hz, 2H); 7.66 (d, *J* = 7.5 Hz, 2H); 7.71 (d, *J* = 15.6 Hz); ^13^C NMR (150 MHz, CDCl_3_, δ (ppm)): δ 42.28 (1 CH_2_); 112.85 (1 CH_2_); 116.93 (1 C); 118.45 (1 C); 123.92 (1 CH); 128.17 (2 CH); 132.60 (2 CH); 139.16 (1 CH); 139.21 (1 C) 164.75 (1 C). FT-IR (cm^–1^): 3266 (vs N–H); 1657 (v C=O); 1615–1550 (C=C aromatic ring); 1061 (vs C–O); 972–913 (*trans* double bond); 825 (*p*-displaced ring). HRMS (ESI) calcd for C_13_H_12_F_3_NO [M + H]^+^, 256.09430, found, 256.09430.

##### 2.1.2.6 (*E*)-*N*-Allyl-3-(4-(trifluoromethoxy)­phenyl)­acrylamide (**AF18**)

White solid, purity: 98%; yield: 90%; mp 120–123 °C. ^1^H NMR (600 MHz, CDCl_3_, δ (ppm)): δ 4.03 (tt, *J* = 1.4, 2.8 e 7.2 Hz, 2H); 5.18 (dd, *J* = 1.4, 11.0 Hz, 1H); 5.24 (dd, *J* = 1.4, 17.0 Hz, 1H); 5.84 (s, 1H); 5.88 (ddt, *J* = 5.7, 11.0, 17.0 Hz, 1H); 6.39 (d, *J* = 15.6 Hz, 1H); 7.21 (d, *J* = 8.0 Hz, 2H); 7.52 (d, *J* = 8.0 Hz); 7.60 (d, *J* = 15.6 Hz, 1H); ^13^C NMR (150 MHz, CDCl_3_, δ (ppm)): δ 42.22 (1 CH_2_); 116.80 (1 CH_2_); 121.16 (1 CH); 121.29 (1 CH); 129.18 (2 CH); 133.42 (1 C); 133.95 (1 CH); 139.74 (1 CH); 165.31 (1 C); FT-IR (cm^–1^): 3072 (vs N–H); 1657 (v C=O); 1610–1503 (C=C aromatic ring); 1084 (vs C–O); 967–908 (*trans* double bond); 825 (*p*-displaced ring). HRMS (ESI) calcd for C_13_H_12_F_3_NO_2_ [M + H]^+^, 272.08896, found, 272.08896.

##### 2.1.2.7 (*E*)-*N*-Allyl-3-(4-nitrophenyl)­acrylamide (**AF19**)

Yellow solid, purity: 100%; yield: 98%; mp 158–160 °C. ^1^H NMR (600 MHz, CDCl_3_, δ (ppm)): δ 4.03 (tt, *J* = 1.4, 3.0, 5.7 Hz); 5.18 (dd, *J* = 17.3, 1.3 Hz); 5.28 (dd, *J* = 10.6, 1.3 Hz); 5.85 (s, 1H); 5.86 (ddt, *J* = 17.3, 10.6, 5.7 Hz, 1H); 6.53 (d, *J* = 15.6 Hz); 7.63 (d, *J* = 8.8 Hz); 7.67 (d, *J* = 15.6 Hz, 1H); 7.71 (ddd, *J* = 8.7, 1.9, 0.5 Hz, 2H); 8.22 (d, *J* = 8.8 Hz, 2H); ^13^C NMR (150 MHz, CDCl_3_, δ (ppm)): δ 42.31 (1 CH_2_); 117.01 (1 CH_2_); 124.15 (2 CH); 124.58 (1 CH); 128.37 (2 CH); 133.70 (1 CH); 138.73 (1 CH); 141.06 (1 C); 148.20 (1 C) 164.57 (1 C); FT-IR (cm^–1^): 3261 (vs N–H); 3072 (vs NO_2_); 1709 (v C=O); 1651–1509 (C=C aromatic ring); 1326–1208 (vas NO_2_); 1084 (vs C–O); 972 (*trans* double bond); 831 (*p*-displaced ring). HRMS (ESI) calcd for C_12_H_12_N_2_O_3_ [M + H]^+^, 233.09218, found, 233.09218.

##### 2.1.2.8 (*E*)-3-(4-Chlorophenyl)-*N*-ethylacrylamide (**AF20**)

White solid, purity: 100%; yield: 70%; mp 146–147 °C. ^1^H NMR (400 MHz, CDCl_3_, δ (ppm)): 7.55 (d, *J* = 16.0 Hz, 1H); 7.43 (d, *J* = 8.4 Hz, 2H); 7.41 (d, *J* = 8.4, 2H); 6.37 (d, *J* = 16.0 Hz, 1H); 5.66 (s, 1H); 3.40 (m, 2H); 1.19 (t, *J* = 7.2, 3H); ^13^C NMR (100 MHz, CDCl_3_, δ (ppm)): 165.46 (C=O); 135.43 (C–Cl); 133.39 (C); 129.01 (CH); 128.91 (CH); 121.30 (CH); 34.67 (CH_2_); 14.88 (CH_3_); FT-IR (cm^–1^): 3225 (vs N–H); 3060 (vs C–H and vas C–H); 2871 (vs CH_2_ and vas CH_2_); 1645 (C=O); 1556 (*trans* double bond); 813 (*p*-substituted ring). HRMS (ESI) calcd for C_11_H_12_ClNO [M + H]^+^, 210.06804, found, 210.06804.

##### 2.1.2.9 Ethylcinnamamide (**AF21**)

White solid, purity: 100%; yield: 88%; mp 91–92 °C. ^1^H NMR (600 MHz, CDCl_3_, δ (ppm)): 7.49 (d, *J* = 16.0 Hz); 7.48 (m, 2H); 7.47 (m, 3H); 6.35 (d, *J* = 16.0 Hz, 1H); 5.68 (s, 1H); 3.39 (m, 2H); 1.18 (t, *J* = 7.2 Hz, 3H); ^13^C NMR (100 MHz, CDCl_3_, δ (ppm)): 165.80 (C=O); 134.89 (C); 140.85 (CH); 129.61 (CH); 120.75 (CH); 128.81 (CH); 127.76 (CH); 14.91 (CH_3_); 34.64 (CH_2_); FT-IR (cm^–1^): 3271 (vs N–H); 2978 (vs C–H and vas C–H); 2908 (vs CH_2_ and vas CH_2_); 2854 (C=O); 1220 (vs C–O); 972 (*trans double*); 716 (singly substituted ring).^55^

### 2.2 Biological Tests

#### 2.2.1 Maintenance of *A. aegypti*

The *A. aegypti* strain used in this study comes from the Laboratory of Organic Chemistry Applied to Materials and Bioactive Compounds of the Institute of Chemistry and Biotechnology of the Federal University of Alagoas, Maceio, Brazil. Eggs of *A. aegypti* were hatched in tap water under static conditions, containing cat food at a temperature and humidity equivalent to 28 ± 2 °C and 80 ± 4%, respectively, at a 12 h photoperiod. Fourth-instar larvae were collected after three days of hatching according to the protocol described by the World Health Organization (WHO),^56^ with some adaptations.^57,58^

#### 2.2.2 Larvicidal Activity

The stock solution (100 μg/mL) was prepared by dissolving 0.01 g of cinnamamide (**AF01**–**AF21**) in 0.02% DMSO (v/v) and 0.01% Tween 80, followed by the addition of distilled water to a final volume of 100 mL. All tests were performed in triplicate by adding twenty-fourth-instar larvae of the vector into containers containing the cinnamamides solution (20 mL) at concentrations ranging from 5 to 100 μg/mL.^59^

Larval mortality was recorded at 48 h, when no larval movement was observed.^56^ The controls consisted of temephos as the positive control and distilled water containing 0.02% DMSO (v/v) and 0.01% Tween 80 as the negative control. The larvicidal assay initially consisted of a qualitative analysis considering the mean mortality percentage (%M) to classify the compound’s potential according to the following activity levels: active (*M* > 75%), moderately active (50% < *M* ≤ 75%), weakly active (25% < *M* ≤ 50%), and inactive (*M* < 25%). When control mortality was below 5%, the mortality percentage was corrected using Abbott’s equation.^60^ Compounds considered promising were subsequently subjected to quantitative analysis, along with assessment of the positive control, by calculating the lethal concentrations required to eliminate 10, 50, and 90% of the organisms (LC_10_, LC_50_, and LC_90_, respectively).^61^

#### 2.2.3 Application of Scanning Electron Microscopy (SEM) in Larval Analysis

Fourth-instar larvae, after 24 h of exposure to (*E*)-*N*-allyl-3-(4-chlorophenyl)­acrylamide **(AF03)**, the positive control (temephos), or the negative control (0.02% v/v Tween 80), were collected and treated for 2 h in a solution of 2.5% (v/v) glutaraldehyde in 0.1 M cacodylate buffer (pH 7.4).^62^ The samples were then dehydrated for 10 min using an ascending ethanol series (15, 30, 60, 90, and 100%). The dehydrated samples were subsequently placed on SEM stubs using graphite double-sided tape, sputter-coated with gold (Quorum Technology, model Q150R ES, East Sussex, UK), and analyzed using a field emission scanning electron microscope (XL30S FEG, Philips Electron Optics B.V., Netherlands) coupled to an EDXS system (energy-dispersive X-ray spectroscopy, Oxford INCA x-act, Oxford Instruments).^63,64^

#### 2.2.4 Assessment of Cell Viability Using the MTT Assay

The effect of the samples on the viability of A549 cells was assessed using the MTT test (3-(4,5-dimethylthiazol-2yl)-2,5-diphenyl tetrazolium bromide). After treating the cells for 24 h with different concentrations of the samples (5, 10, 25, 50, 75, and 100 μg/mL), MTT was added to the culture medium at a final concentration of 5 mg/mL. The culture was kept at 37 °C for 3 h in a humidified CO_2_ incubator. After this time, DMSO was added, and the optical density (OD) was measured by a spectrophotometer at 570 nm, using DMSO as a blank. Cell viability was expressed as a percentage of untreated cells (control, 100%).^65^

#### 2.2.5 Chicken Erythrocytes Function Evaluation

The protocol described by Sales et al. (2022),^66^ was followed, in which oxygen consumption by erythrocytes (Ery) was measured using an OXIGY oxygraph electrode (Hansatech Instruments) in a 1.0 mL glass chamber equipped with a magnetic stirrer. The initial oxygen concentration in the reaction medium at 28 °C was 225 nmol O_2_ mL^–1^. The only difference was that Ery used in this protocol were from chickens,^67^ and whole blood was collected in heparinized tubes. Ery samples were subjected to total protein quantification using the Bradford method,^68^ and we used 325 μg/mL. The experiments were performed in phosphate buffer (0.1 M) at pH 7.4, in which the Ery samples were incubated for 3 min in the presence of different concentrations of the compound **AF03**, in addition to controls corresponding to the solvents used in the solubilization of this compound (DMSO and Tween).

#### 2.2.6 Ecotoxicity Assessment of Cinnamamide AF03 Using Zebrafish

##### 2.2.6.1 Animal Care

Adult wild-type zebrafish were kept in the Laboratory of Mitochondrial Metabolism of Zebrafish (Mitofish), University of Pernambuco (UPE), in the city of Garanhuns. The zebrafish were maintained in a recirculating system at 24 ± 1 °C, and physicochemical parameters were monitored daily. The light/dark cycle was 10 h light/14 h dark, and the zebrafish density in the aquarium was 5 animals/L. The animals were fed once a day with Tropical Poytara and Artemia *spp. ad libitum*. For compound testing, healthy male zebrafish were included in the experimental design. All experimental procedures were evaluated and approved by the Ethics Committee on the Use of Animals of University of Pernambuco (CEUA–UPE; process number 004/2024).

##### 2.2.6.2 In Vivo Exposure Using Zebrafish

The new larvicide **AF03** was administered to adult zebrafish by oral gavage.^69^ The concentrations tested (41.6 and 20.8 μg/mL) were based on the LC_50_ results with *A. aegypti*. Zebrafish were anesthetized using MS-222 (10 mg/mL) for 2 min, and gavage was carried out daily for 3 days. Saline was used as the negative control, and 0.1% DMSO was included as a positive control. After **AF03** administration, the zebrafish were placed in a tank with fresh water for 2 min until recovery from anesthesia. After full recovery, they were transferred to their respective experimental units.^70^

##### 2.2.6.3 Tissue Collection and Homogenate Obtention

The animals were anesthetized using 20 mg/mL MS-222, followed by manual decapitation according to ethical guidelines. Tissues of zebrafish (liver, brain, and heart) were collected after **AF03** exposure and homogenized using a tissue homogenizer (Nova Técnica) in 0.1 M PBS buffer (pH 7.4) containing 137 mM NaCl, 10 mM NaH_2_PO_4_, 1.76 mM KH_2_PO_4_, 1 mM orthovanadate, and 200 μg/mL PMSF, kept on ice. The tissue homogenates were centrifuged at 4 °C and 8000*g* for 10 min. The supernatant was collected and used for the enzymatic assays. The protein content of zebrafish homogenates was determined using the BCA method.^71^

##### 2.2.6.4 Enzymatic Assays

The homogenates obtained from liver, brain, and heart were used to estimate the enzymatic activity of acetylcholinesterase [(AChE) EC 3.1.1.7], superoxide dismutase [(SOD) EC 1.15.1.1], and catalase [(CAT) EC 1.11.1.6].^72–74^ The AChE assay was performed at 405 nm using zebrafish brain homogenate and 0.25 mM DTNB (pH 7.4). AChE activity was monitored for 3 min, and acetylcholine (62 mM) was used as the substrate. Enzymatic activity was determined as the amount of enzyme that hydrolyzes 1 μmol of substrate per minute. The SOD assay was performed using 50 mM glycine buffer (pH 10.0). Activity was monitored for 9 min at 480 nm, and epinephrine diluted in 0.05% acetic acid was used as the SOD substrate. Results represent the SOD capacity to inhibit the autoxidation of 1 μmol of epinephrine. For the CAT assay, 300 mM H_2_O_2_ was employed as the substrate, and the assay was carried out in 50 mM PBS (pH 7.0). The rate of decomposition of H_2_O_2_ over 3 min at 37 °C was used to calculate CAT activity, and absorbance was monitored spectrophotometrically at 240 nm.

### 2.3 Computational Methods

#### 2.3.1 Molecular Docking

Molecular docking calculations were carried out, in which the designed molecules were initially drawn using Chem3D software, optimized with the Spartan program, and saved in the. mol2 format. The molecular docking studies were performed using the GOLD 3.0 program on a Windows 11 PC. The proteins were pretreated by adding polar hydrogens and removing all water molecules. The catalytic region was then selected within a 6 Å search radius. Afterward, 29 targets were screened against compound **AF03** using the scoring function. The binding sites and chemical interactions formed between proteins and ligands were analyzed using BIOVIA Discovery Studio 2019 software, and PyMOL version 2.3.1 was used to create the illustrations.^75,76^

#### 2.3.2 Molecular Dynamics Simulations

The sterol carrier protein-2 from *A. aegypti* (AeSCP-2) (PDB: 2KSI, available at: 10.2210/pdb2KSI/pdb) complexed with **AF03**, initially obtained through docking simulations, was further investigated using molecular dynamics (MD) simulations over a 100 ns time frame. The MD simulations were conducted on a high-performance Asus desktop computer (Taipei, Taiwan), equipped with a 13th-generation Intel Core i9 processor (5.8 GHz), 128 GB RAM, and an NVIDIA GeForce RTX 3080 GPU with 12 GB GDDR6 memory and 8960 CUDA cores, operating on a Linux platform. The macromolecular complex was prepared for simulations using the Protein Preparation Wizard module available in the Desmond software suite, integrated within the Maestro environment from Schrödinger 2023.4 (https://www.deshawresearch.com/resources.html). Initially, the complex was optimized at a physiological pH of 7.4 by using the PROPKA module. Then, sodium (Na^+^) and chloride (Cl^–^) ions were added to achieve a 0.15 M concentration, approximating physiological conditions, while the TIP3P explicit solvent model was applied to simulate water molecules. The complex was then placed within an orthorhombic simulation box, ensuring an appropriate microenvironment for the system. Energy minimization of the system was carried out using the OPLS_2005 force field to optimize initial atomic positions and reduce the potential energy during a preliminary 10 ns minimization run. Subsequently, a 100 ns MD simulation was performed, maintaining a constant temperature of 300 K using a Nose–Hoover thermostat and a pressure of 1.031 bar with a Martyna–Tobias–Klein barostat. Following the 100 ns MD simulation, a comprehensive clustering analysis was performed to identify the most representative conformations of the complex. Postsimulation analyses included the generation of key trajectory profiles, such as root-mean-square deviation (RMSD), root-mean-square fluctuation (RMSF), and detailed ligand–target interaction diagrams. These analyses provided crucial insights into the dynamic behavior, structural stability, and interaction patterns within the **AF03**-AeSCP-2 complex. All procedures were performed according to previously published studies from our research group.^77–79^

### 2.4 Statistical Analysis

The biological tests (larvicide and cytotoxicity) were carried out in triplicate, and the results were expressed as the mean ± standard deviation of the measurements taken. The data were submitted to analysis of variance (ANOVA) combined with Dunnett’s method for multiple comparisons.^80,81^ GraphPad Prism software was used for these statistical analyses, while the probit method was employed for quantitative larvicide analysis. The CLAD quantification was then processed using Origin Pro 9 software (Northampton, MA). Linear regression using the least-squares method was applied to obtain parameters such as the slope (α), intercept (b), coefficient of determination (*r* ^2^), and correlation coefficient (*r*). For the toxicity and enzymatic tests involving zebrafish, the data were subjected to the Shapiro–Wilk test for normality and Levene’s test for homogeneity of variances. A one-way ANOVA followed by Tukey’s post hoc test was also performed. The level of significance was set at *p* < 0.05.

## 3 Results and Discussion

### 3.1 Synthesis

The synthesis of cinnamamides was carried out using two main approaches: the Steglich reaction (**Scheme 1**) and the reaction between acyl chlorides and amines (**Scheme 2**) (Figure 1). In the Steglich reaction, 12 cinnamamides were synthesized with yields ranging from 40 to 70%. To increase yields and reduce reaction time, the cinnamic acid derivatives were converted into acyl chlorides and then reacted with primary amines, resulting in the synthesis of 9 cinnamamides, all with yields of over 75%. A total of 21 cinnamamides were synthesized, 9 of which were new. All of the compounds were characterized using ^1^H and ^13^C NMR and FT-IR.

**Figure 1:**
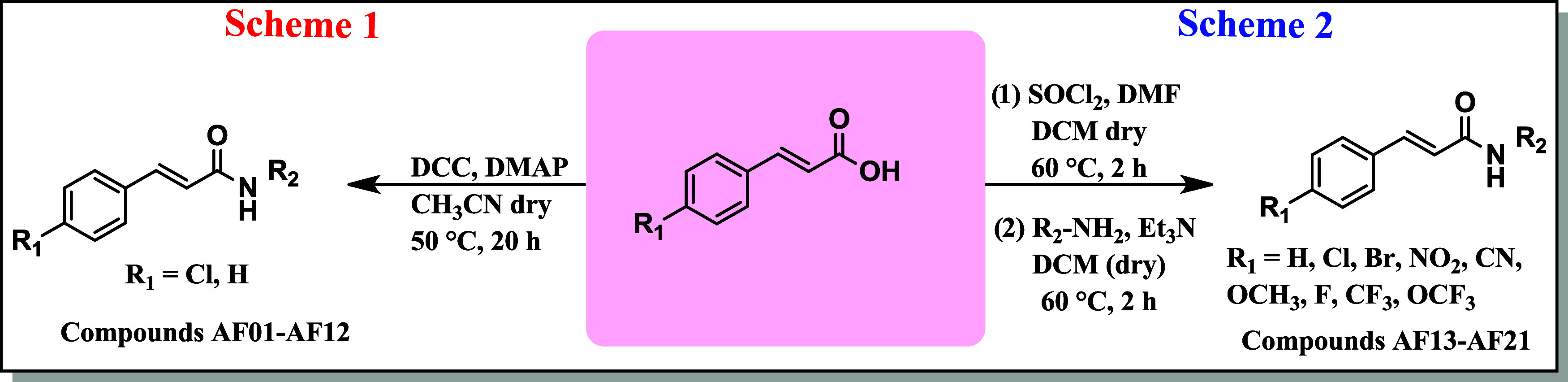
Synthesis of cinnamamides.

### 3.2 Screening of Cinnamamides against *A. aegypti* Larvae

Once all of the cinnamamides had been obtained, they were submitted to a preliminary larvicidal test. In this context, the results of the larvicidal test (Table 1) indicated that, among the compounds evaluated, two showed a promising larvicidal action, according to the WHO classification: **AF03** and **AF04**. Of these, cinnamamide **AF03**, which has a chlorine atom in the *para* position of the cinnamoyl portion, stood out as the most active of the series tested. At a 95% confidence interval, there was no statistically significant difference from the positive control (temephos). On the other hand, cinnamamide **AF04**, which has an aliphatic portion in the side chain to the amide group, showed promise in the preliminary larvicidal screening. These two compounds (**AF03, AF04**) have an alkyl chain with 3 to 6 carbon atoms attached to the amide function of the molecules, respectively. In addition, two other cinnamamides stood out as being partially active: **AF05** and **AF11**, which have aromatic substituents on the side chain to the amide, noting that the increase in aromatic bonds may have caused a reduction in larvicidal efficacy, leading the compounds to be partially active.

**Table 1:**
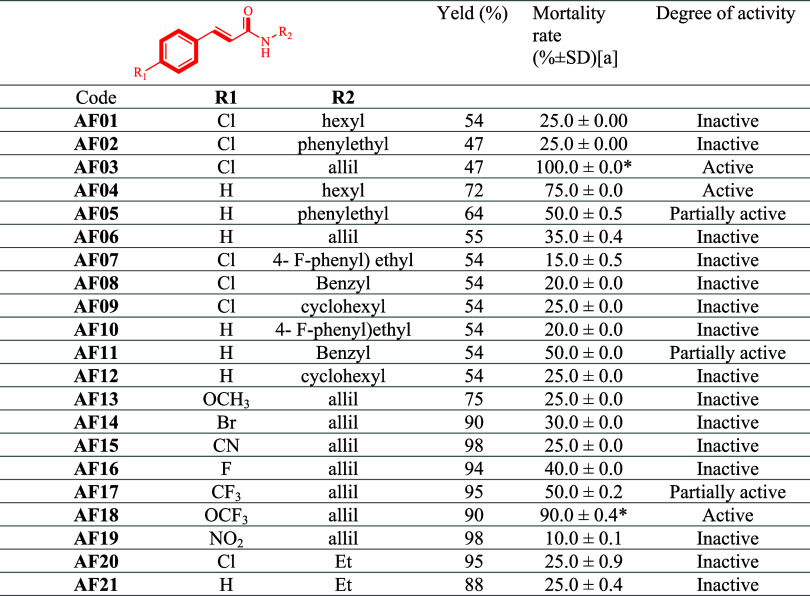
Screening of Larvicidal Activity against *A. aegypti* and Synthesis Yield of Cinnamic Amides^a^

a Mean of the three replicates with (±) standard deviation (mean, ±; n = 3) SD. A concentration of 100 μg/mL was used. * There is no significant difference in a 95% confidence interval (ANOVA ONE-WAY) compared to the reference compound (10 μg/mL).

In addition, this study compared the compounds ethylcinnamamide (**AF21**) and (*E*)-3-(4-chlorophenyl)-*N*-ethylacrylamide (**AF20**) (Table 1) with the esters ethyl cinnamate and ethyl *p*-chlorocinnamate reported by França et al. (2021),^43^ which showed mortality percentages of 95 and 100%, respectively, both at a concentration of 45 mg/mL. These compounds, which are called bioisosteres, consequently have similar physicochemical properties. Despite this, unlike the esters, compounds **AF20** and **AF21** were not promising regarding their larvicidal activity. One of the hypotheses in this regard is related to interactions in the biological target. Esters are more reactive compounds and, therefore, favorable to hydrolysis, unlike amides, which are much more stable species and less susceptible to hydrolysis.^82^

The active and partially active cinnamides were subjected to a quantitative analysis to obtain the lethal concentration for 50, 90, and 10% of a population (LC_50_, LC_90_, and LC_10_), as shown in Table 2. The LC_50_ of the insecticide temephos was also calcd for comparison purposes. Quantitative analysis showed an increase in the LC_50_ value for temephos when compared to the value reported in the literature (LC_50_ of 3.0 μg/mL), which may be related to the accelerated metabolization of this larvicide by Aedes larvae.^83^ As far as cinnamamides are concerned, temephos has a more significant larvicidal effect when compared to the promising compounds in this study, among which **AF03** is the most active of the compounds evaluated, showing the lowest LC_50_ value (Table 2), followed by cinnamamide **AF04**, **AF05**, and **AF11**, which have aromatic substituents.

**Table 2:**
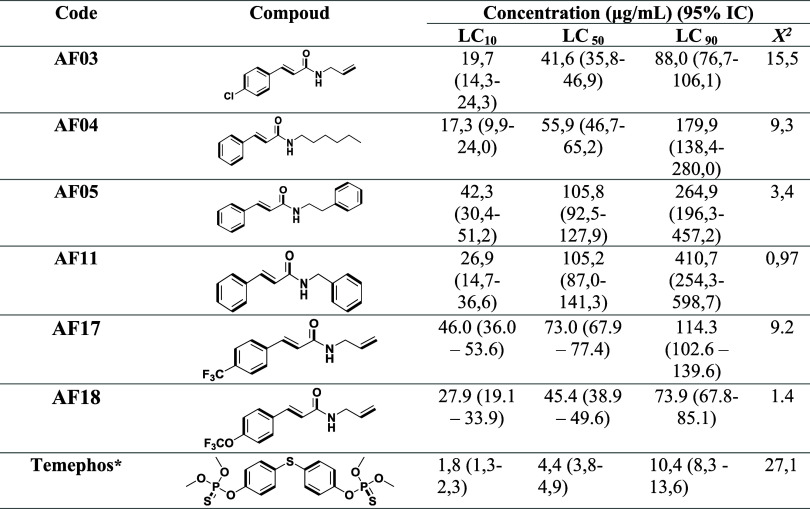
Quantitative Evaluation of the Larvicidal Activity of Cinnamamides against *A. aegypti* Larvae in the Fourth Stage, after 48 h^a^

a CI: 95% confidence interval; LC: lethal concentration; χ^2^ (Chi): degree of validation; * positive control.

To obtain a compound with a lower LC_50_ value, and based on the structure of compound **AF03**, seven new cinnamamides (**AF13**-**AF19**) were synthesized with different electron donor and electron-withdrawing atoms in the *para* position of the cinnamoyl portion (OCH_3_, F, CF_3_, OCF_3_, Br, CN, NO_2_) (Table 1). Only compounds **AF17** and **AF18** showed larvicidal activity against the vector’s larvae, with LC_50_ values of 45.4 and 73.0 μg/mL, respectively (Table 2).

### 3.3 Scanning Electron Microscopy

Morphological analysis of the larva exposed to the 100 μg/mL concentration of compound AF03 reduced the body mass and deformity in the midgut region of the larva. Deformities were also noted in the siphon region, along with slight shrinkage in the anal papillae (Figure 2). This organ is involved in regulating electrolyte levels^84^ and the ability to absorb sodium, potassium, chloride, and phosphate ions from the environment; this function was reduced or lost in larvae without gills. This indicates that the lack or dysfunction of the anal gills probably led to an interruption in osmotic and ionic regulation.^64,84,85^ As seen in Figure 2, the damage caused by the compound occurred mainly in the midgut. This region performs several essential functions, such as digestion, absorption of nutrition, transport of ions, ionic and osmotic regulation, storage of lipids and carbohydrates, control of the pH of the midgut lumen, and secretion of digestive enzymes.^86^

**Figure 2:**
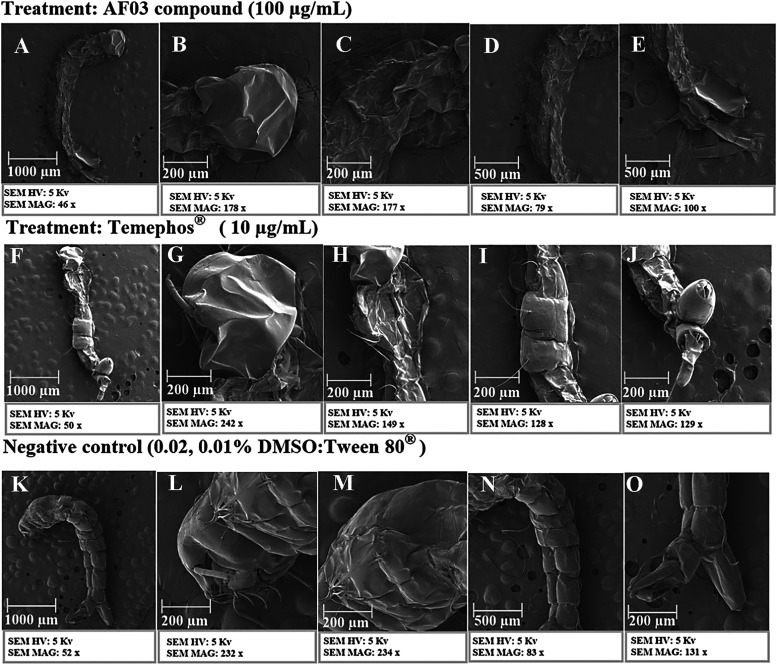
Application of SEM for morphological analysis of larvae exposed to the treated groups (compound **AF03**, temephos, and negative control). Legend: (A–E) Larvae treated with **AF03**; (F–J) larvae exposed to temephos; (K–O) larvae treated with temephos; (B, G, and L) larval head; (C, H, and M) thorax; (D, I, and N) abdomen; (E, J, and O) anal papillae and siphon.

The midgut mainly comprises epithelial cells supported by a basal membrane that lines the body wall. Similar to other insects, the larval stomach functions not only in digestion but also in chemical and mechanical defense against pathogens.^87^ These harmful changes in the organism’s midgut indicate that they are a joint response to cellular intoxication. Therefore, the larvae’s degenerative responses lead to dysfunction, which can cause death.^88^

### 3.4 Molecular Docking

As observed in the morphological analysis results, it is assumed that the biological targets involved may be receptor proteins, transporters, or digestive system proteins. In this sense, molecular docking calculations were carried out with compound **AF03** against different targets present in the fourth-stage larva of the vector under study, with 29 structures available in the Protein Data Bank (PDB). The results pointed to two macromolecules whose FitScore value was higher: sterol carrier protein (PDB: 2ksi) and kynurenine aminotransferase (PDB: 1yiy) (Figure 3A). Subsequently, these two targets were selected for docking calculations involving the other compounds, whose LC_50_ was determined experimentally, to compare. The results shown in Figure 3B indicated that the macromolecule sterol carrier protein (PDB: 2ksi) exhibited the best performance according to the FitScore value and was therefore chosen for molecular dynamics studies (Figure 3B). The *Ae*SCP-2 protein and kynurenine aminotransferase (*Ae*KAT), including insects, are expressed throughout the animal kingdom. *Ae*KAT is a multifunctional enzyme that catalyzes the transamination of various amino acids and is expressed mainly in adults’ heads, indicating its essential role in the central nervous system.^89–91^ This enzyme catalyzes the conversion of L-kynurenine to kynurenic acid. *Ae*KAT is essential for neuromuscular homeostasis and larval development, influencing the balance between quinolinic and kynurenic acid, which are fundamental for growth and protection against oxidative damage.^90^ In addition, it is a promising target for insecticides and chemical interventions. The inhibition of this could jeopardize the mosquito’s life cycle, helping to control diseases transmitted by *A. aegypti*.^92^

**Figure 3:**
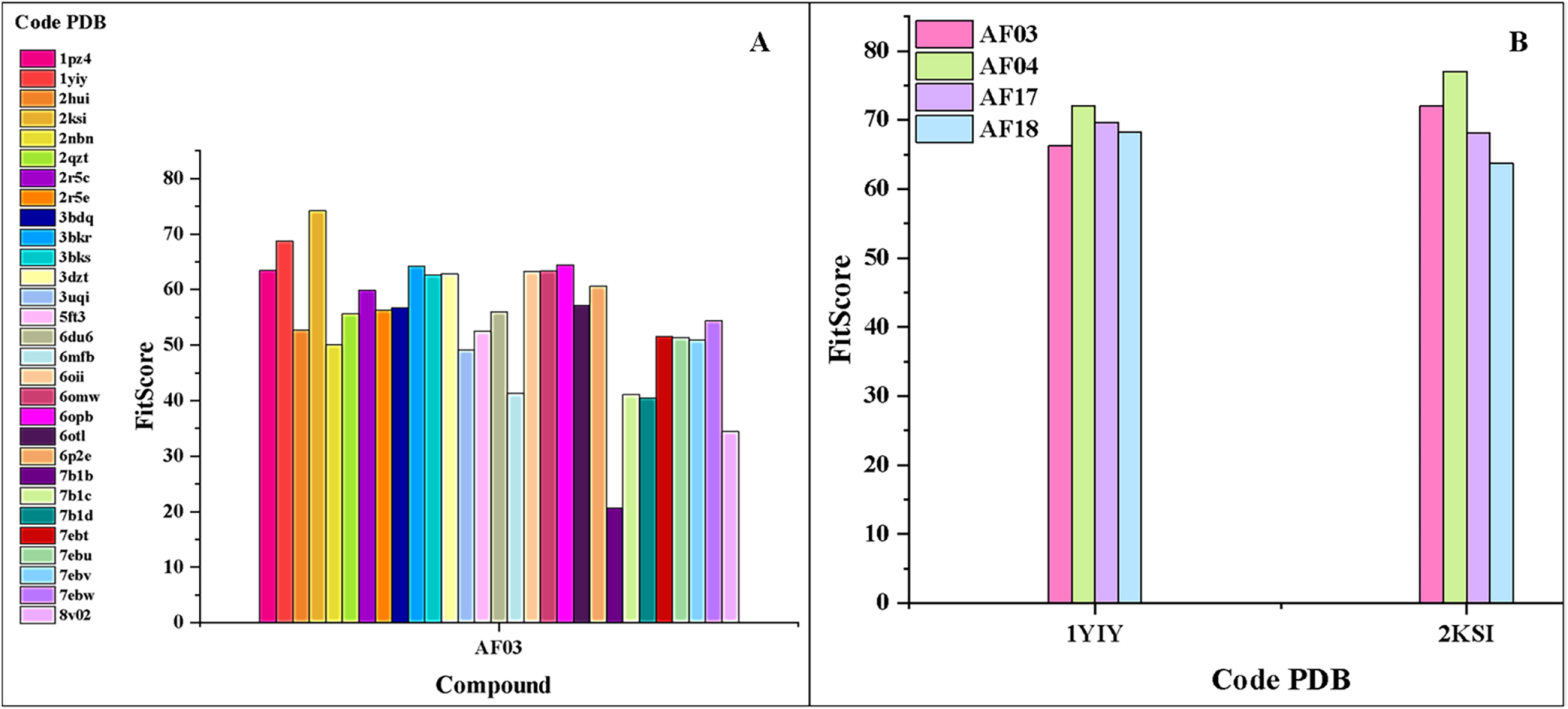
(A) Result of the docking calculation for compound **AF03** against 29 macromolecules with the ChemPLP scoring function; (B) FitScore of the most active compounds against the most promising targets selected after screening with compound **AF03**.

*Ae*SCP-2 is an intracellular lipid transporter located in the midgut and is responsible for the delivery and uptake of cholesterol across the cellular barrier between the hemocele and the midgut. This protein is vital in absorbing cholesterol and fatty acids, contributing to essential lipid metabolism in both larvae and adult mosquitoes.^93,94^

*Ae*SCP-2 has an α/β-fold conformation, creating a central hydrophobic cavity ideal for binding lipid molecules. Analysis of the interactions of compound **AF03** in the active site of *Ae*SCP-2 reveals that the compound is positioned within this cavity and stabilized by van der Waals interactions with hydrophobic residues. These interactions involve 17 amino acid residues belonging to the protein’s active site cavity.^95,96^ The interactions include van der Waals interactions with VAL^8^, ILE1^9^, and MET^46^, the pi–pi stacked interaction with PHE^109^, and the interaction of the ARG^15^ residue with the chlorine atom of the cinnamoyl portion of the compound (Figure 4).

**Figure 4:**
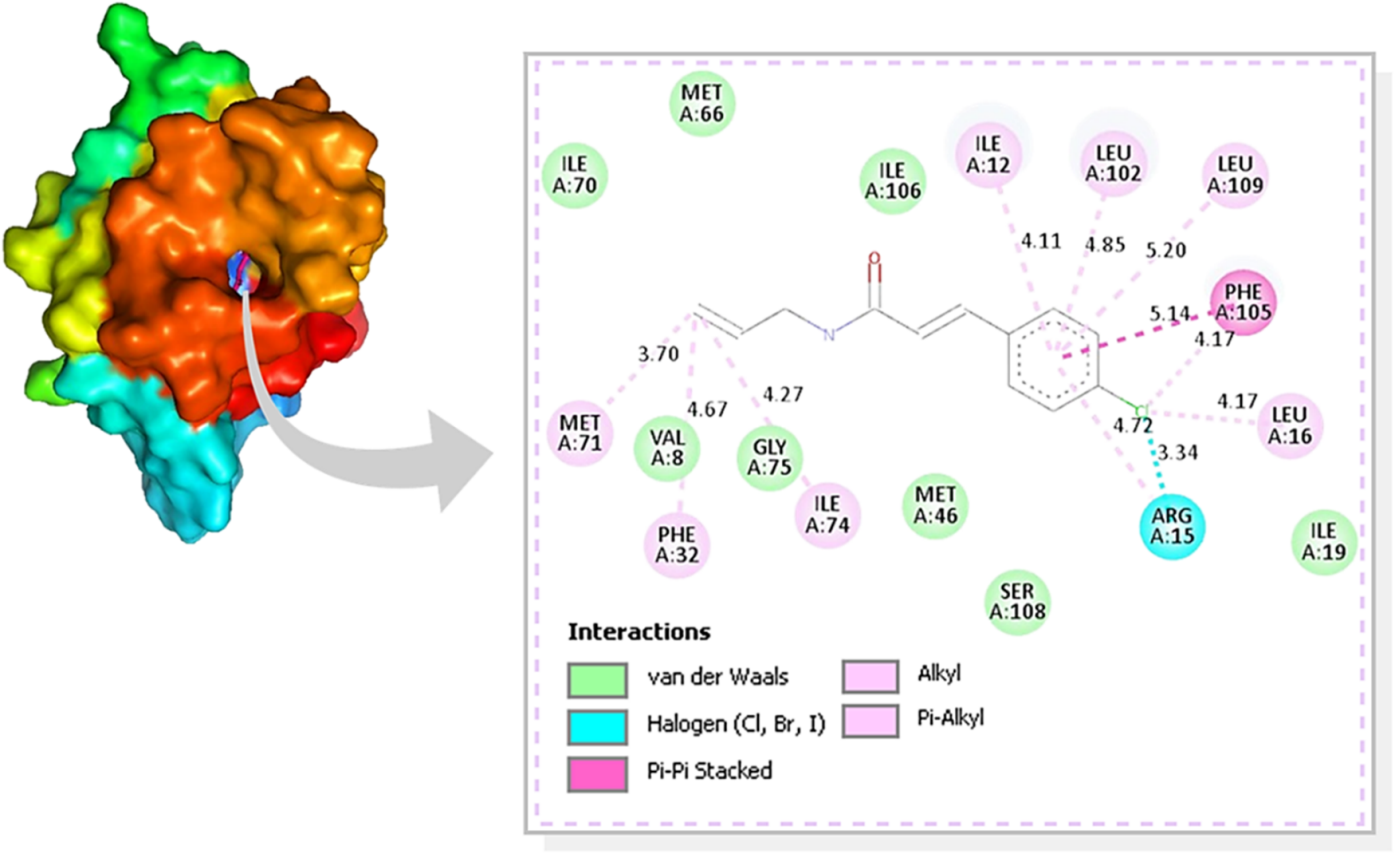
Interactions between the amino acid residues of the *Ae*SCP-2 active site and compound **AF03**.

### 3.5 Molecular Dynamics

The molecular dynamics (MD) simulation of compound **AF03** bound to *Ae*SCP-2 provided comprehensive insights into the binding stability and structural dynamics of the protein–ligand complex over a 100 ns simulation. The analyses included root-mean-square deviation (RMSD), root-mean-square fluctuation (RMSF), and its intermolecular interactions, each offering valuable details about the system’s behavior during the simulation. The RMSD profiles (Figure 5 **A**) for both the protein backbone and **AF03** were monitored to assess the structural stability and binding consistency. The protein backbone RMSD values ranged between 2.0 and 3.0 Å throughout the simulation, indicating that *Ae*SCP-2 maintained its structural integrity without significant unfolding or destabilization. During the first ∼10 ns, the system underwent an initial stabilization phase, reflecting its relaxation to equilibrium. Minor fluctuations observed after 20 ns likely correspond to natural protein dynamics rather than to any structural instability. The RMSD plot of **AF03**, calculated relative to the protein, exhibited fluctuations between 0.5 and 2.0 Å, which suggests that the ligand maintained stable binding throughout the simulation. Occasional peaks, such as the one observed at ∼20 ns, may be attributed to transient conformational rearrangements of the ligand within the binding pocket. Importantly, the ligand’s RMSD remained consistently lower than that of the protein backbone, reinforcing the hypothesis of a stable and specific interaction between **AF03** and *Ae*SCP-2. The RMSF values (Figure 5 **B**) for Cα atoms provided residue-level insights into protein flexibility during the simulation. The majority of residues exhibited RMSF values below 1.0 Å, indicating that *Ae*SCP-2’s secondary structure remained stable throughout the simulation. These stable regions likely correspond to α-helices and β-sheets, which are characterized by their structural rigidity. Peaks in the RMSF plot, particularly at the N- and C-terminal regions, indicate greater flexibility. Such behavior is expected, as terminal regions often lack strong secondary structure and experience enhanced mobility in solution. Moderate RMSF values in the ligand-binding pocket suggest a degree of flexibility that facilitates ligand accommodation and supports induced-fit binding mechanisms. This flexibility likely enhances the specificity of **AF03** binding by enabling localized conformational adjustments. The low RMSD values for **AF03** indicate the formation of a stable complex with *Ae*SCP-2. This stability may be attributed to strong noncovalent interactions, including hydrogen bonds, van der Waals forces, and π-stacking interactions within the binding pocket. The RMSF data demonstrate that while *Ae*SCP-2 retains overall structural stability, localized flexibility in specific regions, including the binding pocket, supports dynamic interactions with the ligand. Such flexibility may be essential for the protein’s biological function in sterol transport. The combined results from RMSD and RMSF analyses suggest that **AF03** binding does not perturb the global stability of *Ae*SCP-2. Instead, the ligand leverages the localized flexibility within the binding pocket to establish optimal interactions, which could contribute to its efficacy as an inhibitor. Additionally, it is verified in the RMSF plot that **AF03** interacts preferentially in α-helices structures, comprising 30–65 residues indexed. Additionally, complementary analyses of the MD simulation of **AF03** bound to *Ae*SCP-2 were performed, providing insights into residue-level interactions, structural properties, and specific binding contacts, respectively. Figure 5C illustrates the interaction fraction for the **AF03-** *Ae*SCP-2 interaction, in which Leu^48^, Leu^102^, and Phe^105^ showed high interaction fractions, suggesting their critical roles in stabilizing the protein–ligand complex. These interactions predominantly involve hydrophobic contacts. Moreover, Figure 5D provides metrics related to the protein–ligand complex’s structural stability and exposure. In this context, the radius of gyration (Rg) shows fluctuations around a consistent range (∼4.35 Å), indicating that the protein maintains its compact structure throughout the simulation. Molecular surface area (MolSA) shows minor fluctuations (ranging from 232 to 234 Å^2^), reflecting changes in the protein’s solvent-accessible regions. This suggests that the protein undergoes slight conformational rearrangements while remaining structurally stable. Then, the solvent-accessible surface area (SASA) plot shows variations in SASA, especially around 20–40 ns. These fluctuations likely correspond to transient rearrangements of hydrophobic residues interacting with **AF03**. Besides, the polar surface area (PSA) plot indicates low PSA values (48–52 Å^2^), which is consistent with the dominance of hydrophobic interactions in the binding pocket, as observed in Figure 5C. Finally, the 2D-ligand interaction map (Figure 5 **E**) provides a detailed map of specific residues involved in hydrophobic and π–π stacking interactions with **AF03**. Residues such as Ileu^19^, Phe^32^, Leu^48^, Ile^74^, Leu^101^, and Leu^102^ perform hydrophobic contacts via van der Waals interactions. The presence of π–π stacking interactions with Tyr^30^ and Phe^105^ residues enhances the specificity and stability of **AF03**. Moreover, some of these residues were observed in a previous study reported by Sivasankaran et al.^97^ Furthermore, the MD simulation results demonstrate that **AF03** forms a stable and specific interaction with *Ae*SCP-2. The protein’s overall structural stability, coupled with the ligand’s low RMSD values, reinforces the reliability of the binding mode predicted by docking studies. The moderate flexibility observed in the binding pocket highlights its adaptive nature, potentially enabling the high-affinity binding of **AF03**. These findings provide a robust foundation to validate **AF03** as a potential inhibitor of *Ae*SCP-2.

**Figure 5:**
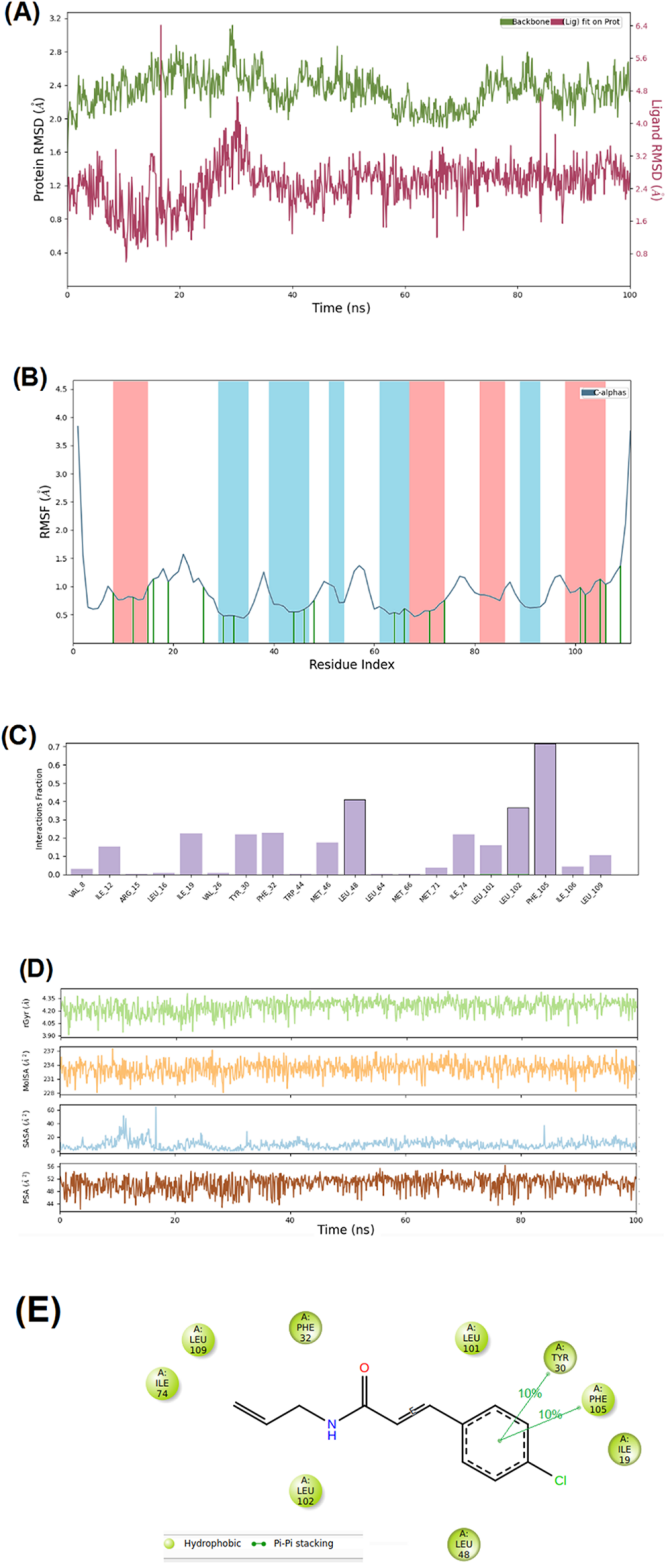
Molecular dynamics (MD) simulations for AF03-*Ae*SCP-2 complex within a 100 ns time frame. (A) Root-mean-square deviation (RMSD) plot for *Ae*SCP-2 (green line) and **AF03** (pink line). (B) Root-mean-square fluctuation (RMSF) plot for *Ae*SCP-2 in complex with **AF03**, in which beige barriers mean β-sheets, blue barriers represent α-helices, whereas green lines mean **AF03** contacts. (C) Interaction fraction shows hydrophobic interactions (purple barriers). (D) Green plot, radius of gyrations; Orange plot, molecular surface area; Blue plot, solvent-accessible surface area; Brown plot, polar surface area. (E) Map of interactions for **AF03** in complex *Ae*SCP-2.

### 3.6 Physicochemical Properties

In line with the molecular docking results, the physicochemical properties of the compounds evaluated (Figure 6) are within the norms that provide for the biorational development of a selective and environmentally safe insecticide,^98^ where for all of the compounds, it was possible to obtain MM < 435 Da, ClogP < 6, HBA < 6, HBD ≤ 2, nRBO < 9, and nARB < 17. Among these parameters, nRBO and CLogP stand out, which relate to flexibility and permeability in the insect’s lipophilic cuticle, respectively, in which the cinnamamides in question were similar to cinnamic acid derivatives containing the hydrazide group.^99^

**Figure 6:**
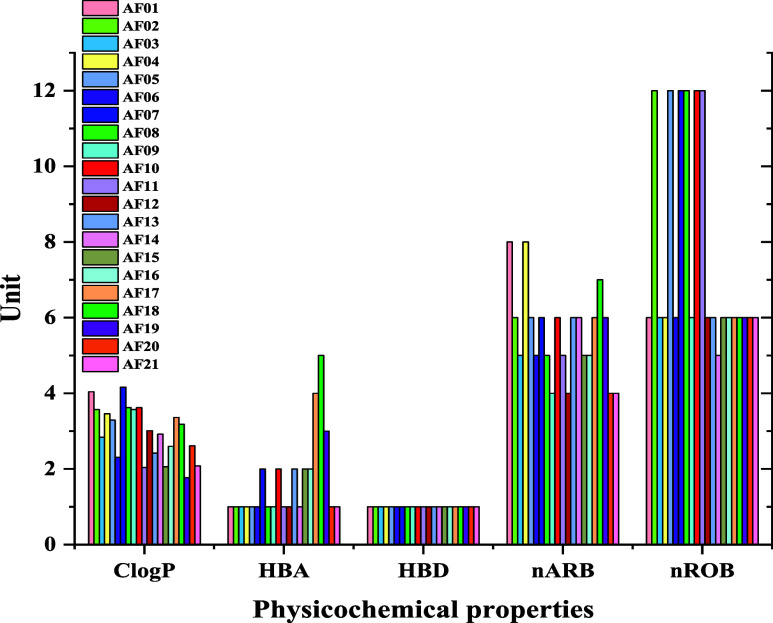
Physicochemical properties of the molecules under study.

### 3.7 Cellular Cytotoxicity

Finally, the most promising **AF03** and **AF18** compounds in the tested series were subjected to cytotoxicity analysis against human lung cancer cell lines (**A549**). Metabolic activity was then assessed using the colorimetric MTT assay in which formazan formation was quantified spectrophotometrically at 570 nm (Figure 7). Thus, according to the experimental data, none of the compounds tested affected **A549** cell viability in the range of 5 to 100 μg/mL compared to the control. The results corroborate what has already been reported in the literature about the low toxicity of cinnamic derivatives against the same cell line used in this study.^23,100^

**Figure 7:**
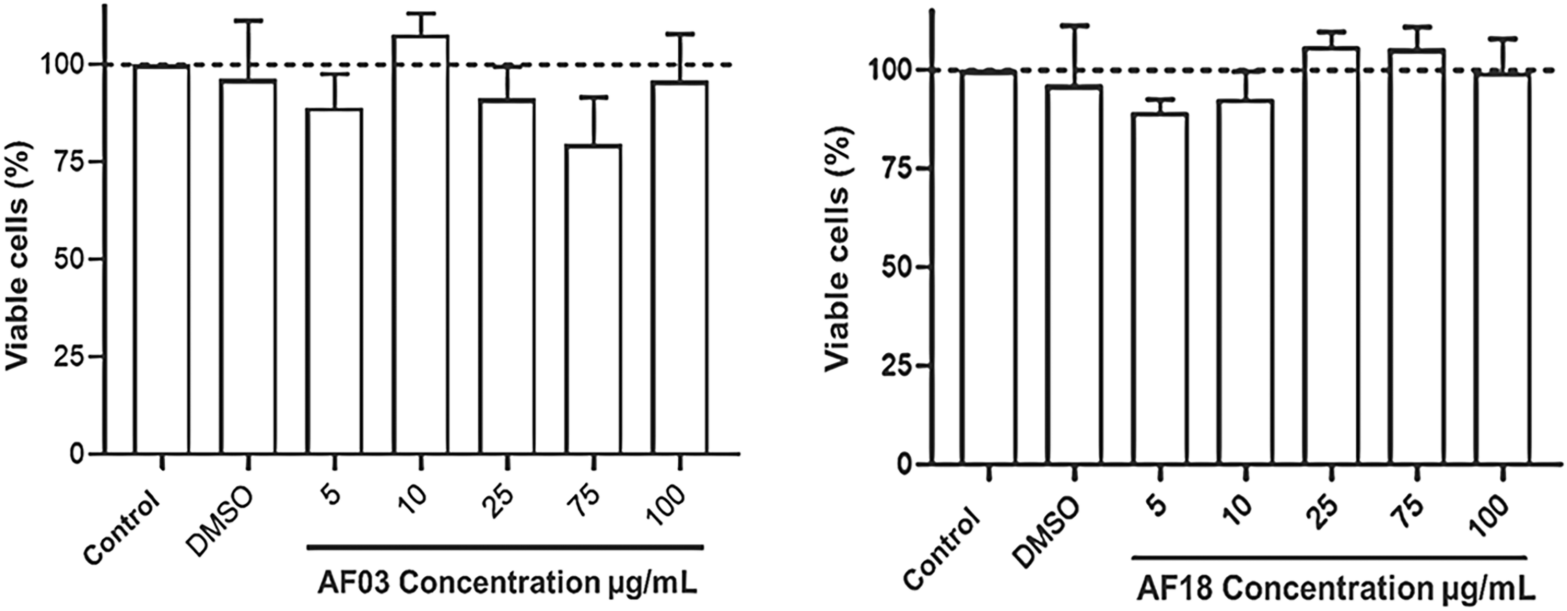
Effect on the viability of **A549** human airway epithelial cells assessed by an MTT assay after 24 h of incubation with various concentrations. The results are expressed as a percentage of the control cells. The values are reported as the mean ± the SD of three independent experiments.

### 3.8 Chicken Erythrocyte Function

Following the biological evaluations focusing on toxicity assays, we performed another type of study using erythrocytes from healthy chicken. Erythrocytes have as main function the oxygen transport that is directly performed by the protein hemoglobin. This protein has a heme ring where the iron center has an affinity to oxygen. In this study, we verify that the more promised molecule, **AF03**, showed no statistical difference when compared to the control (Figure 8). To avoid the possibility of DMSO or Tween interference, we also performed control experiments just with them, in the higher and lower concentrations used to solubilize the drug. The compromising of hemoglobin capacity to transport oxygen is indicative of toxicity, as shown in experiments conducted in human erythrocytes in the presence of ethylmercury, or mercury itself.^66,101^ In this same way, erythrocytes from hypercholesterolemic mice exposed to HgCl_2_ showed a decrease in oxygen uptake.^102^ In the present study, the compound **AF03** did not show any change in chicken erythrocytes’ main function as an oxygen carrier.

**Figure 8:**
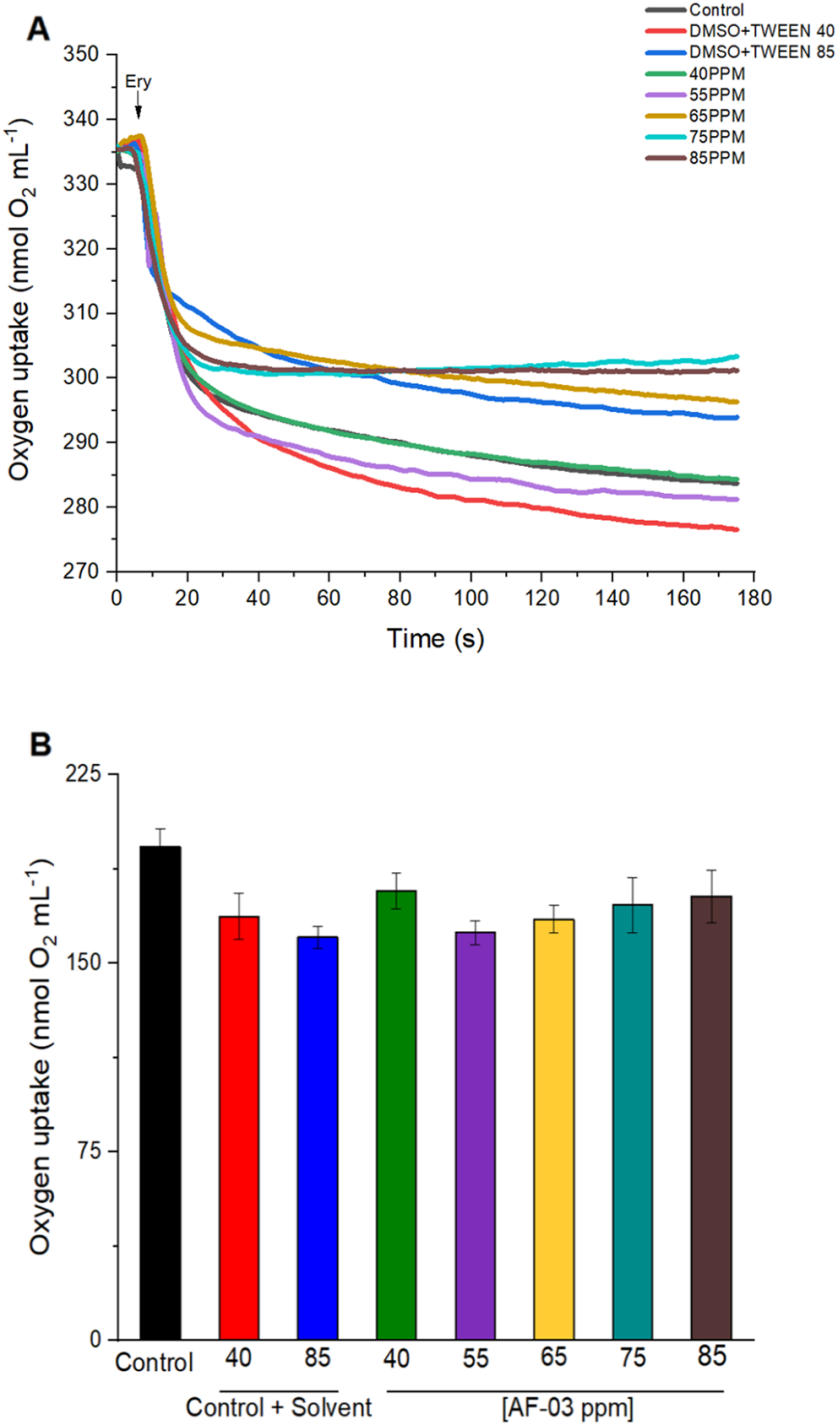
Oxygen uptake in erythrocytes exposed to AF03: (A) Representative graph where each line represents the oxygen consumption in erythrocytes at 28 °C of the control and solvent control (DMSO + Tween) groups, equivalent to the concentrations used of the **AF03** compound solutions at 40 and 85 ppm, respectively. The arrow indicates the addition of erythrocytes. (B) Quantification of the maximum oxygen consumption capacity for 10 s. The data represent *n* = 4 independent experiments performed in duplicate. One-way ANOVA followed by Tukey’s post hoc test was applied. The results did not show statistically significant differences between the groups.

### 3.9 Zebrafish Physiological Responses

During the exposure, the zebrafish gills appeared a little irritated, especially at the concentration of 41.6 μg/ml of cinnamamide **AF03**. However, the irritation does not show an impact on the behavior of zebrafish. This includes food preferences that remained unchanged during the test. This result is particularly compelling because behavioral changes are commonly observed even at low doses of agrochemicals. The identification of effects can provide early indicators of toxicity to nontarget organisms like zebrafish and signalize to effects of sublethal exposures. Furthermore, no animals died during the exposure, indicating that **AF03** at the concentrations tested was not acutely lethal.

To expand the understanding of possible sublethal effects, the enzymes AChE, SOD, and CAT were studied. Acetylcholinesterase is a well-known biomarker of neurotoxicity for a wide range of pesticides, as its inhibition is a common mechanism that leads to toxic effects in the nervous system of nontarget organisms. The result of the enzymatic activity of brain AChE is represented in Figure 9. The results show that both **AF03** concentrations increase AChE activity (*p* = 0.001). These results can be related to a potential physiological adjustment of the zebrafish nervous system, which is crucial for a nontarget organism to maintain neural function while safety environmental changes were carried out. Considering a possible influence on the redox system, the enzymes (catalase), CAT, and superoxide dismutase (SOD) were also evaluated. CAT is a key enzyme to perform hydrogen peroxide decomposition, and a myriad of conditions can impair the enzymatic activity. The results of the CAT assay using zebrafish liver, brain, and heart are presented in Figure 10. In the zebrafish liver, the results show that **AF03** at 41.6 μg/mL increases CAT levels or activity (*p* = 0.02). However, this difference was also observed in the positive control adopted, suggesting that these effects are more likely associated with DMSO + Tween, which are well-known because of the toxic effects. More than that, it is plausible to infer a possible synergism between **AF03** and the solvent, which reduces hepatic toxicity, as indicated by the results registered at 20.8 μg/mL. The results of the zebrafish brain show that any difference was observed (Figure 10B). In the zebrafish heart, the treatments of DMSO+Tween and **AF03** at 41.6 μg/mL increase the CAT activity when compared to the negative control (*p* = 0.01 and *p* = 0.05, respectively). The superoxide dismutase (SOD) assay demonstrated an increase in SOD activity across all treated groups compared to the saline control (*p* < 0.01). It is important to note that the results obtained with **AF03** are similar to those of the solvent used. In the zebrafish brain (Figure 11B), both tested concentrations appeared to increase SOD activity, possibly as a physiological adjustment after **AF03** administration. In the zebrafish heart, none of the tested **AF03** concentrations showed a significant effect on the SOD activity, indicating a lower impact. In summary, the multisystemic approach employed is helpful to gain a deeper understanding of the effects of **AF03** on different tissues.

**Figure 9:**
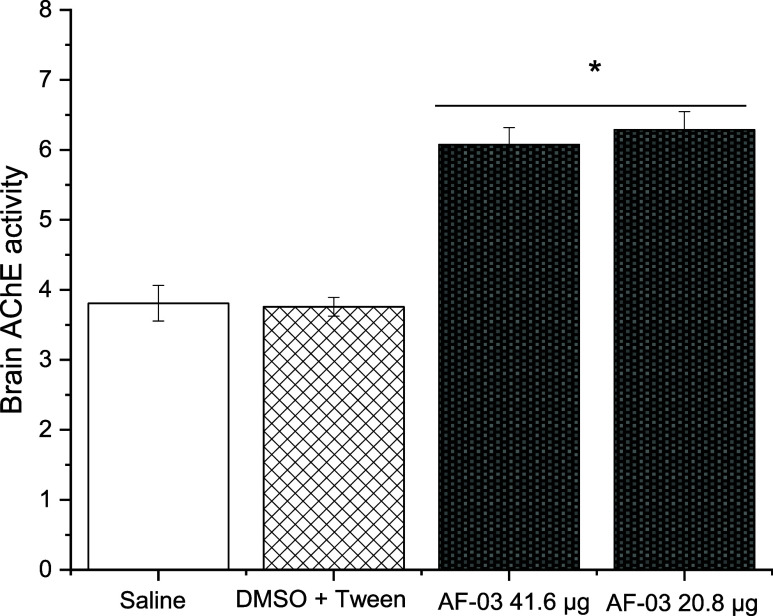
Zebrafish acetylcholinesterase activity. The acetylcholinesterase (AChE) activity was measured using zebrafish brain homogenate. The values are means ± SD (*n* = 3). * *p* < 0.001.

**Figure 10:**
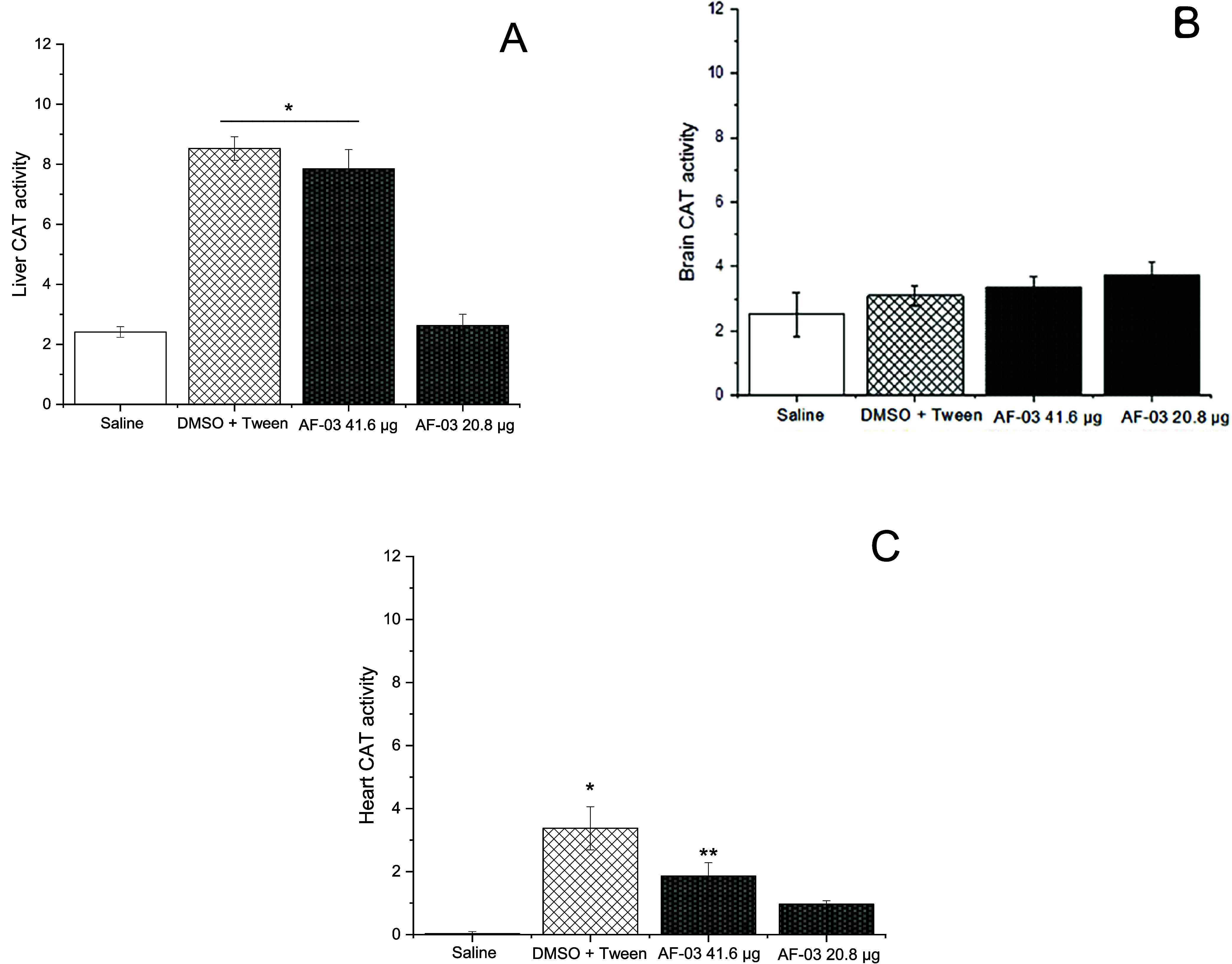
Zebrafish catalase (CAT) activity. The catalase activity was represented in the liver (A), brain (B), and heart (C) of adult zebrafish; no statistical differences were observed between the groups tested. Bars are means ± SE, and the values are the units of enzyme activity estimated as the amount of the catalase that decomposes 1 μM of H_2_O_2_ per minute. In zebrafish liver, * *p* = 0.01, and in zebrafish heart, * *p* = 0.03, ** *p* = 0.05. (*n* = 3).

**Figure 11:**
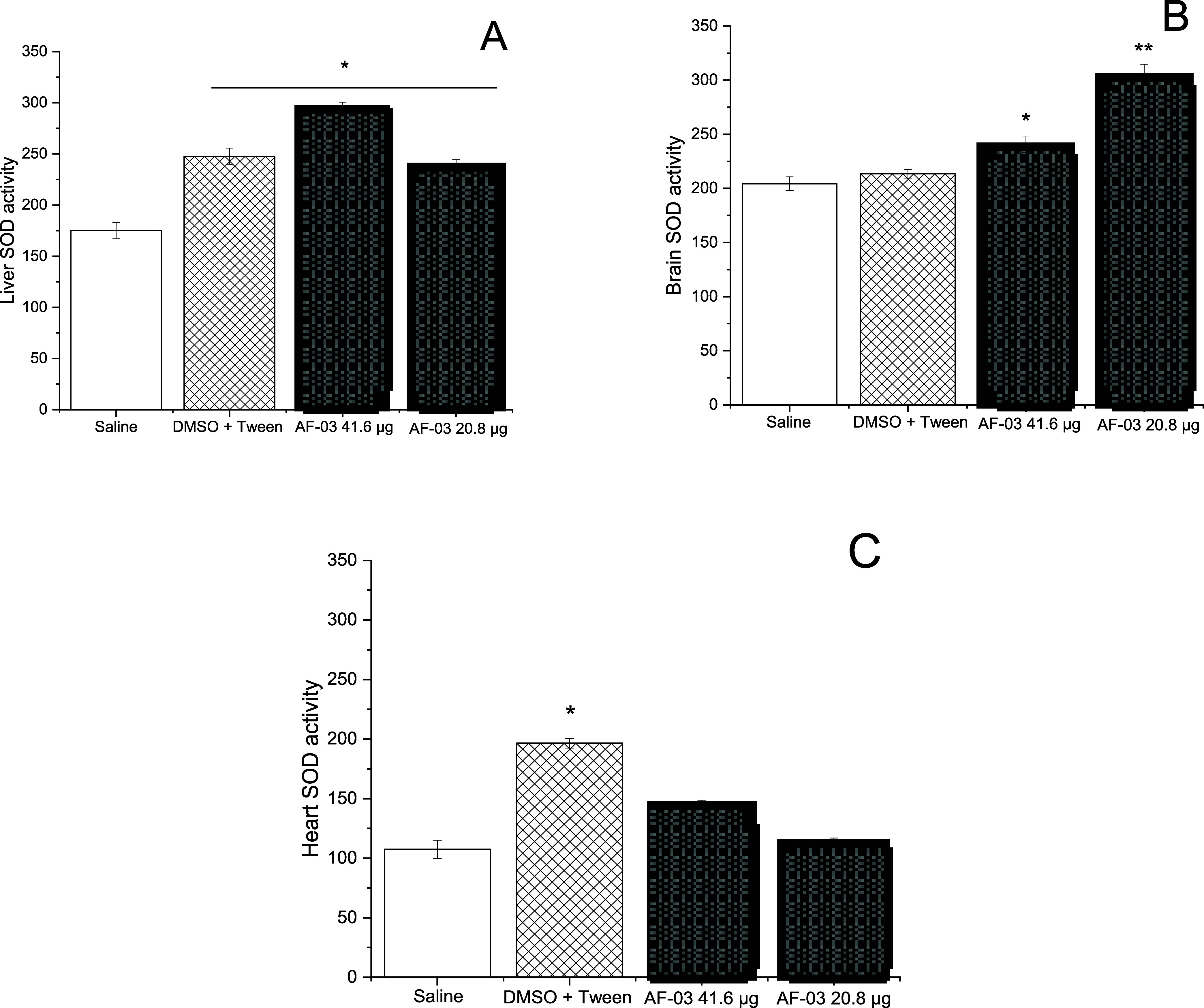
Zebrafish superoxide dismutase activity. The SOD activity was represented in adult zebrafish. The results are means ± SD of the area under the SOD curve. Liver SOD is represented in part (A) * *p* = 0.001. Brain SOD is represented in part (B) * *p* = 0.01, ** *p* = 0.001, and heart homogenate is represented in part (C) * *p* < 0.001. (*n* = 3).

## 4 Conclusion

The synthesis of cinnamamides was carried out using two main approaches and resulted in 21 compounds, nine of which were new. In the larvicidal study, compounds **AF03** and **AF18** were the most promising, with LC_50_ values of 41.6 and 45.4 μg/mL, respectively, against *A. aegypti* larvae. Morphological analysis by SEM showed that **AF03** caused significant changes in essential structures of the larvae, such as the midgut and anal gills, affecting their homeostasis and possibly leading to mortality. The molecular docking study indicated that **AF03** interacts with the *Ae*SCP-2 protein, which is a relevant target for new larvicides. This finding was reinforced by molecular dynamics studies, which confirmed the stability of the compound’s binding to the protein’s active site. In addition, the compounds tested showed physicochemical properties compatible with the criteria for developing selective and environmentally safe larvicides. The cell viability of compounds **AF03** and **AF18** was assessed in A549 adenocarcinoma cells, and no cytotoxic effect was observed. Consistently, the functionality of erythrocytes was not compromised, and their capacity for O_2_ uptake was maintained in the presence of **AF03**. In addition, short-term ecotoxicological bioassays indicated that the species *D. rerio* (zebrafish) was not sensitive to **AF03**, nor were any neurotoxic changes detected in the redox system of this animal model, which reinforces the low toxicity already described for cinnamic derivatives. Overall, this study provides unprecedented evidence of the larvicidal activity of cinnamamides and highlights **AF03** as a promising candidate for the control of *A. aegypti*. These findings contribute to the advancement of new strategies to combat arboviruses. However, further studies are needed to test its efficacy in actual environmental conditions and to evaluate possible structural modifications that could improve its larvicidal activity.

## Supplementary Material



## Notes

Experimental section; ^1^H NMR, ^13^C DEPT NMR, and FT-IR spectra of cinnamic acids and cinnamamides; HRMS data and HPLC chromatograms of cinnamamides. The Supporting Information is available free of charge at https://pubs.acs.org/doi/10.1021/acsomega.5c08177.

Experimental section; ^1^H NMR, ^13^C DEPT NMR, and FT-IR spectra of cinnamic acids and cinnamamides; HRMS data and HPLC chromatograms of cinnamamides (PDF)
