# Supplementary material for: New Cinnamamide and Derivatives as New Larvicides against Aedes aegypti Vector Larvae: Facile Synthesis, In Silico Study, In Vitro Noncytotoxicity, and Nontoxicity against Zebrafish
Source: ACS Omega. 2025 Nov 23;10(48):59078–95. doi: 10.1021/acsomega.5c08177 (PMC12771257; doi:10.1021/acsomega.5c08177)
Supplement: Supplementary file 1 [file ao5c08177_si_001.pdf]

Support Information

A new cinnamamide and derivatives: facile synthesis, new larvicide against *Aedes aegypti* vector larvae, *in silico* study, non-cytotoxic *in vitro* and non-toxic against zebrafish.

Adrielle Firmino da Silva,[a] Saraliny Bezerra França,[a] Erick Gabriel Alves Ferreira,[b] Emiliano de Oliveira Barreto,[b] Jenniffer Mclaine Duarte de Freitas,[c] Johnnatan Duarte de Freitas,[c] Edeildo Ferreira da Silva Júnior,[a] Ana Catarina Rezende Leite,[a] Pedro Correia Gomes dos Santos,[a] Rafael David Souto de Azevedo,[d] Josefa Gerlane da Silva,[d] Dimas José da Paz Lima*[a]

[a] *Institute of Chemistry and Biotechnology, Federal University of Alagoas, Maceió, Alagoas, 57072-970, Brazil.*
[b] *Research Group on Biological and Molecular Chemistry, Institute of Chemistry and Biotechnology, Federal University of Alagoas, AC. Simões campus, Lourival Melo Mota Avenue, 57072-970, Maceió, Alagoas, Brazil.*
[c] *Federal Institute of Alagoas, Rua Mizael Domingues, 530, Maceió, Alagoas 57020-600, Brazil.*
[d] *University of Pernambuco – UPE, Campus Garanhuns. 552904-902. São José. Garanhuns – PE. Brazil.*

* Corresponding author: Dimas J.P. Lima, Tel.+55 82 3214-1384,

e-mail: dimas.lima@iqb.ufal.br

SI. No.	Table of Contents	Page No.
1	Experimental section	2
2	Spectroscopic analysis of cinnamic acids derivatives	3-5
3	^{1}H NMR and DEPT ^{13}C spectra of cinnamic acids	6-13
4	FTIR spectra of cinnamic acids	14-17
5	^{1}H NMR and DEPT ^{13}C spectra of cinnamamides	18-38
6	FTIR spectra of cinnamamides	39-49
7	HRMS and HPLC of cinnamamides	49-60

Support Information

1. Experimental section

Synthesis of cinnamic acids used as starting material

Synthesis of cinnamic acids: 0.250g (2.4 mmol) of malonic acid, 0.281g (2.0 mmol) of aldehyde, solubilized in 0.328 mL (4.08 mmol) of pyridine were added to a 25 mL Schlenk tube. The system was stirred at 60 ºC for 20 min at reflux; then 0.023 mL (0.24 mmol) of piperidine was added. After 24 hours, 1.1 mL of ice-cold distilled water was added to the reaction medium, still stirring. Subsequently, 1 mL of 37% HCl was added to an ice bath and stirred for 15 min, after which the pH was checked, which should be on a scale of pH = 1. Finally, vacuum filtration was carried out with ice-cold distilled water to obtain the product without the need for purification. This procedure leads to a yield of over 80%.

Synthesis of cinnamamides AF01 to AF12

Method A: 0.75 mmol of the acid, 0.058 mL (0.43 mmol) of the amine, and 0.75 mmol of dimethylaminopyridine (DMAP) were added to 5 mL of CH_3CN (anhydrous) in a 25 mL flask. The system was kept stirring at a temperature of 0º C. A solution of 0.79 mmol of dicyclohexylcarbodiimide (DCC) dissolved in 5 mL of CH_3CN was then transferred drop by drop. The reaction mixture was kept in an argon atmosphere at 60º under stirring for 24 hours. When the reaction was complete, the mixture was transferred to a separatory funnel, diluted with 10 mL of CH_3CN and washed three times: 3 x 15 mL with 5% (w/v) HCl, 3 x 15 mL with 10% $KHCO_3$ and 3 x 15 mL with H_2O. The separated organic phase was dried with anhydrous Na_2SO_4, filtered, and rotaevaporated at reduced pressure. The solid material was then purified by silica gel column chromatography using hexane/acetate (8:2) as the mobile phase.

Synthesis of cinnamides AF13 to AF21

Method B: the first reaction consisted of using $SOCl_2$ to form the acid chloride. In a 25 mL flask, 1 mmol of the acid was added, solubilized in 3 mL of DCM (anhydrous), and

0.1 mmol of DMF (anhydrous). The system was kept stirring at reflux in an inert atmosphere for the subsequent addition of 5 mmol of $SOCl_2$ at 0°C. The system was then heated to 60° and stirred for 2 hours. The acid chloride formed was rotoevaporated at reduced pressure and solubilized in DCM for the subsequent amide formation step. In the second reaction step, 1 mmol of the amine and 1.4 mmol of Et_3N were added to a 25 mL flask and solubilized in DCM (anhydrous). The solution containing the acid chloride was then kept at a temperature of 0°C. The mixture was stirred for 2 hours at 60°C. The product formed was transferred to a separating funnel, diluted with 10 mL of ethyl acetate and subjected to three washing processes: 3 x 15 mL with 5% (w/v) HCl, 3 x 15 mL with 10% $KHCO_3$ and 3 x 15 mL with H_2O.

2. Spectroscopic analysis of cinnamic acids derivatives

(*E*)-3-(4-(trifluoromethyl)phenyl)acrylic acid

RMN ^{1}H (400MHz, DMSO$_{d6}$, δ (ppm)): 10.02 (s, 1H); 7.91 (d, *J*= 8.2 Hz, 2H); 7.64 (d, *J*= 8.2 Hz, 2H); 6.66 (d, *J*= 16.0 Hz, 1H); 4.20 (d, *J* = 16.0 Hz, 1H).

RMN ^{13}C (100MHz, DMSO$_{d6}$, δ (ppm)): 167.68 (C=O); 138.73 (C-Cl); 126.17 (C); 142.57 (1 CH); 129.31 (2 CH); 126.17 (2 CH); 122.61 (1 CH).

FT-IR (cm^{-1}): 1686 (ν_δ -C=C-CO-O- of carboxylic group); 1633, 1409 (v C=C of aromatic); 1303 (v-C-O); 813 (*p*-disubstituted ring).

(*E*)-3-(4-(trifluoromethoxy)phenyl)acrylic acid

RMN ^{1}H (400MHz, DMSO$_{d6}$, δ (ppm)): 10.02 (s, 1H); 5.32 (d, *J*= 8.0 Hz, 2H); 5.10 (d, *J*= 16.0 Hz, 2H); 4.85 (d, *J*= 16.0 Hz, 1H); 4.02 (d, *J* = 8.0 Hz, 1H).

RMN ^{13}C (100MHz, DMSO$_{d6}$, δ (ppm)): 167.84 (C=O); 134.05 (C); 130.66 (C); 142.70 (1 CH); 130.66 (2 CH); 121.78 (2 CH); 120.96 (1 CH).

FT-IR (cm^{-1}): 1680 (ν_δ -C=C-CO-O- of carboxylic group); 1627 (v C=C of aromatic); 1261 (v-C-O); 825 (p-disubstituted ring).

(*E*)-3-(4-methoxyphenyl)acrylic acid

RMN ^{1}H (400MHz, DMSO$_{d6}$, δ (ppm)): 12.30 (s, 1H); 7.61 (d, *J* = 8.8 Hz, 1H); 7.51 (d, *J* = 16.0 Hz, 1H); 6.96 (d, *J* = 8.8 Hz, 2H); 6.34 (d, *J* = 16.0 Hz, 1H); 3.78 (s, 1H).

RMN ^{13}C (100MHz, DMSO$_{d6}$, δ (ppm)): 168.29 (C=O); 161.40 (C); 127.28 (C); 144.81 (1 CH); 130.40 (2 CH); 116.96 (1 CH); 114.81 (1 CH).

FT-IR (cm^{-1}): 1668 (ν_{δ} -C=C-CO-O- of carboxylic group); 1589-1504 (v C=C of aromatic); 1315 (v-C-O); 820 (*p*-disubstituted ring).

(*E*)-3-(4-cyanophenyl)acrylic acid

RMN ^{1}H (400MHz, DMSO$_{d6}$, δ (ppm)): 12.6 (s, 1H); 7.9 (d, *J* = 6.4 Hz, 4H); 7.6 (d, *J* = 16.0 Hz, 1H); 6.7 (d, *J* = 16.0 Hz, 1H).

RMN ^{13}C (100MHz, DMSO$_{d6}$, δ (ppm)): 167.9 (C=O); 139.3 (C); 119.0 (C); 112.6 (C); 142.4 (1 CH); 133.2 (2 CH); 129.2 (2 CH); 123.2 (1 CH).

FT-IR (cm^{-1}): 1686 (ν_{δ} -C=O of carboxylic group); 1600-1415 (v C=C of aromatic); 1303 (v-C-O); 825 (*p*-disubstituted ring).

(*E*)-3-(4-nitrophenyl)acrylic acid

RMN ^{1}H (400MHz, DMSO$_{d6}$, δ (ppm)): 12.71 (s, 1H); 8.22 (d, *J* = 8.0 Hz, 2H); 7.96 (d, *J* = 8.0 Hz, 2H); 7.66 (d, *J* = 16.0 Hz, 1H); 6.71 (d, *J* = 16.0 Hz, 1H).

RMN ^{13}C (100MHz, DMSO$_{d6}$, δ (ppm)): 167.50 (C=O); 148.41 (C); 141.20 (C); 141.81 (1 CH); 129.77 (2 CH); 124.40 (2 CH); 124.07 (1 CH)

FT-IR (cm^{-1}): 3119-2314 (v O-H); 1687 (ν_{δ} -C=O of carboxylic group); 1631-1522 (v C=C of aromatic); 1333 (v-C-O); 833 (*p*-disubstituted ring).

(*E*)-3-(4-chlorophenyl)acrylic acid

RMN ^{1}H (400MHz, DMSO$_{d6}$, δ (ppm)): 7.71 (d, *J*= 7.9 Hz, 2H); 7.55 (d, *J*= 16.0 Hz, 1H); 7,48 (d, *J*= 8.3 Hz, 2H); 6.53 (d, *J*= 16.0 Hz, 1H); 12.48 (s, 1H).

RMN ^{13}C (100MHz, DMSO, δ (ppm)): 167.87 (C=O); 135.17 (C-Cl); 133.67 (C); 142.98 (CH); 130.39 (2 CH); 129.38 (2 CH); 120.53 (CH).

FT-IR (cm^{-1}): 3100-2300 (v O-H); 1668 (v C=O of carboxylic group); 1614-1409 (v C=C of aromatic); 1309-1273 (v C-O); 825 (*p*-disubstituted ring).

(*E*)-3-(4-fluorophenyl)acrylic acid

RMN ^{1}H (400MHz, DMSO$_{d6}$, δ (ppm)): 7.77 (dd, *J* = 8.0, 5.5 Hz, 2H); 7.56 (d, *J*= 16.0

Hz, 1H); 7.24 (t, *J*= 8.0 Hz, 2H); 6.24 (d, *J* = 16.0 Hz, 1H); 12.42 (s, 1H).

RMN ^{13}C (100MHz, DMSO, δ (ppm)): 167.99 (C=O); 162.37 (C-F); 131.38 (C); 143.33 (CH); 131.36 (2 CH); 119.61 (2 CH); 116.22 (CH).

FT-IR (cm^{-1}): 3100-2300 (v O-H); 1668 (v C=O of carboxylic group); 1614-1409 (v C=C of aromatic); 1309-1273 (v C-O); 825 (*p*-disubstituted ring).

(*E*)-3-(4-bromophenyl)acrylic acid

RMN ^{1}H (400MHz, $DMSO_{d6}$, δ (ppm)): 7.60 (dd, *J* = 8.0, 1.5 Hz, 4H); 7.57 (d, *J*= 16.0 Hz, 1H); 6.53 (d, *J* = 16.0 Hz, 1H); 12.46 (s, 1H).

RMN ^{13}C (100MHz, DMSO, δ (ppm)): 167.9 (C=O); 134.0 (C-Br); 123.9 (C); 143.0 (1 CH); 132.3 (2 CH); 130.6 (2 CH); 120.6 (1 CH).

FT-IR (cm^{-1}): 3100-2200 (v O-H); 1674 (v C=O of carboxylic group); 1610-1403 (v C=C of aromatic); 1285 (v C-O); 807 (*p*-disubstituted ring).

3. ^{1}H NMR and DEPT ^{13}C spectra of cinnamic acids

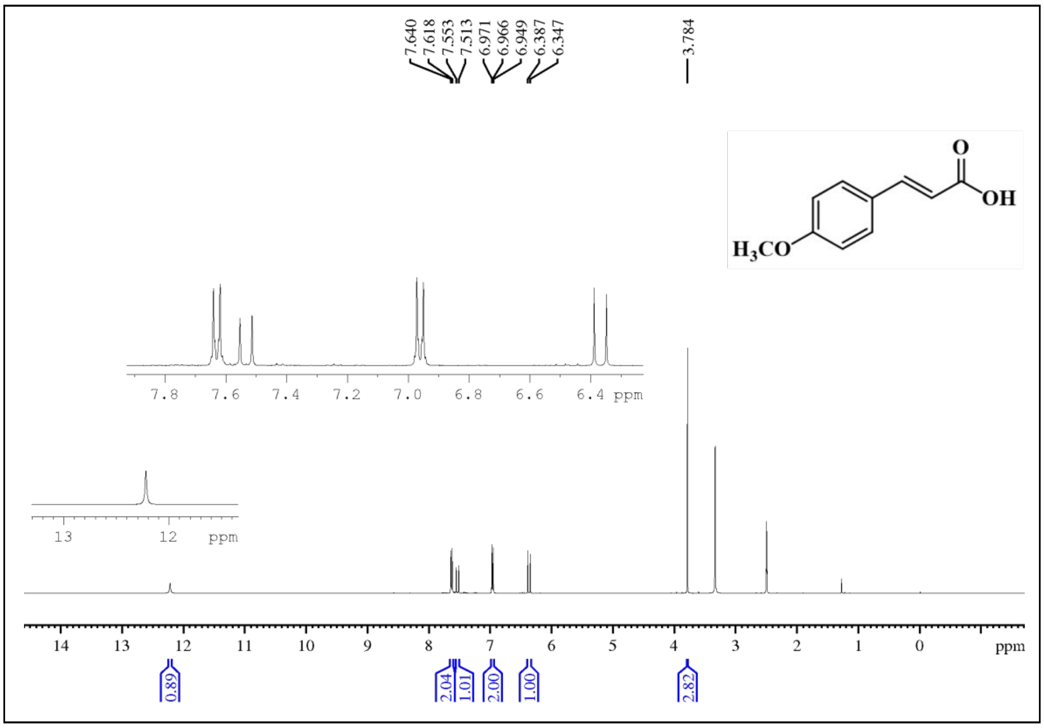

Figure S1. ^{1}H NMR of (*E*)-3-(4-(trifluoromethoxy)phenyl)acrylic acid

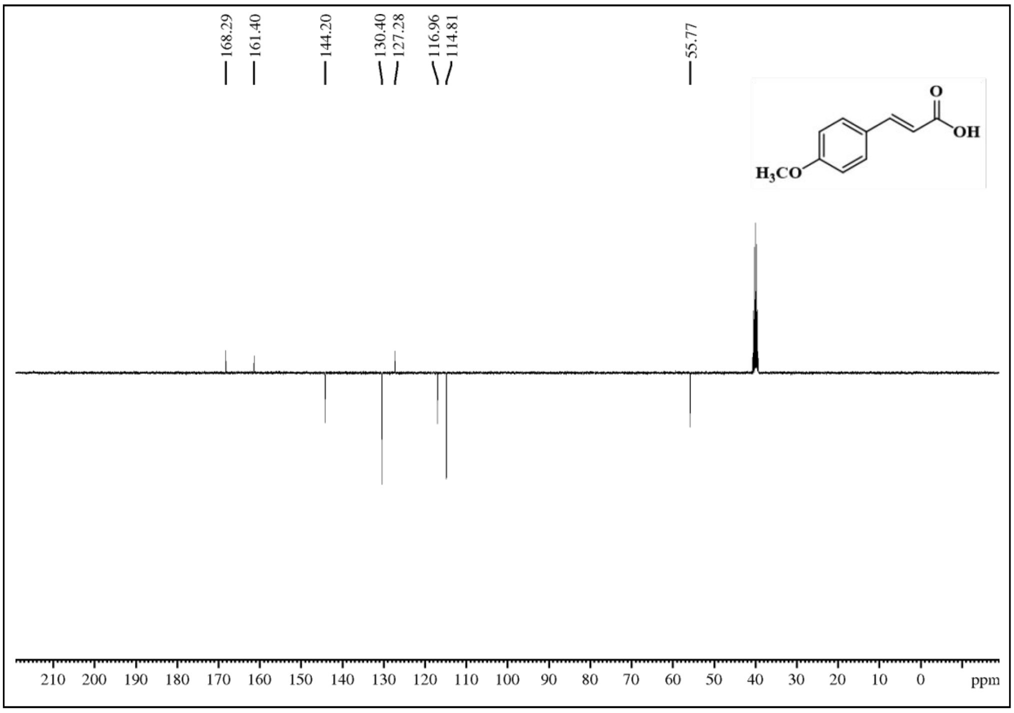

Figure S2. ^{13}C NMR of (*E*)-3-(4-(trifluoromethoxy)phenyl)acrylic acid

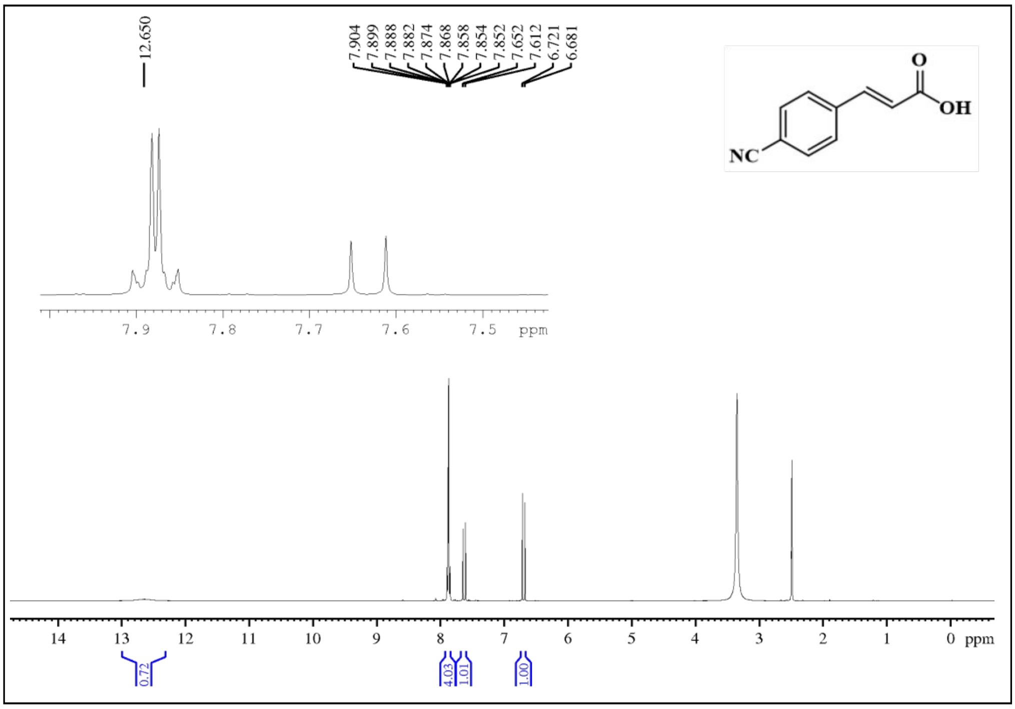

Figure S3. ^{1}H NMR of (*E*)-3-(4-cyanophenyl)acrylic acid

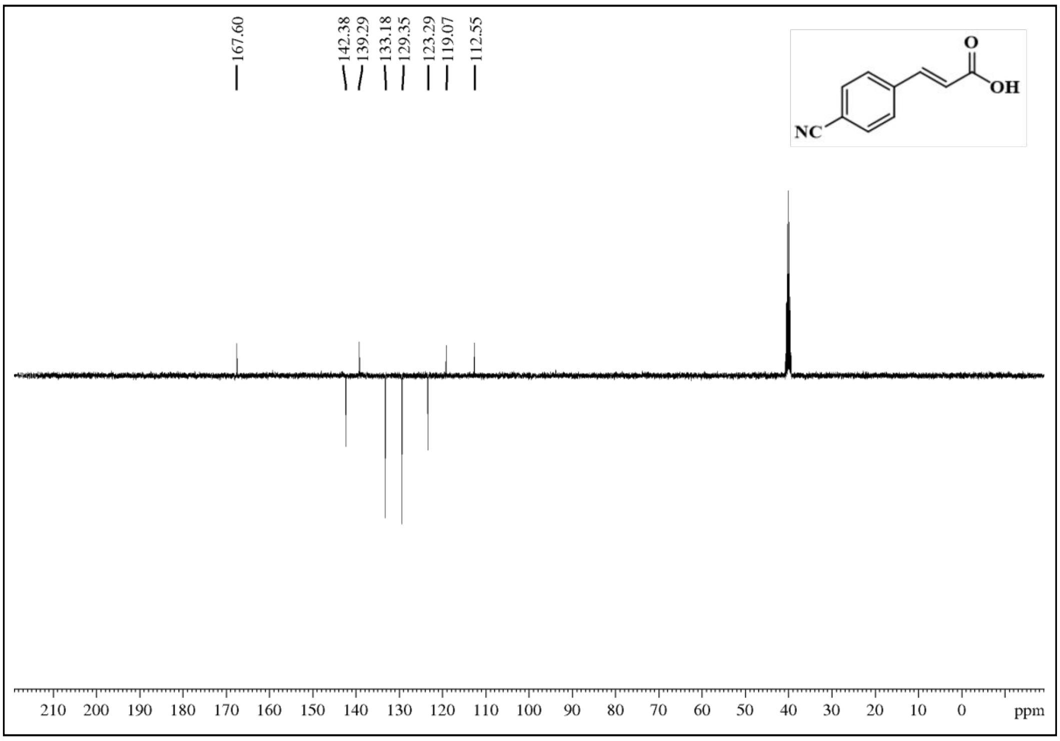

Figure S4. ^{13}C NMR of (*E*)-3-(4-cyanophenyl)acrylic acid

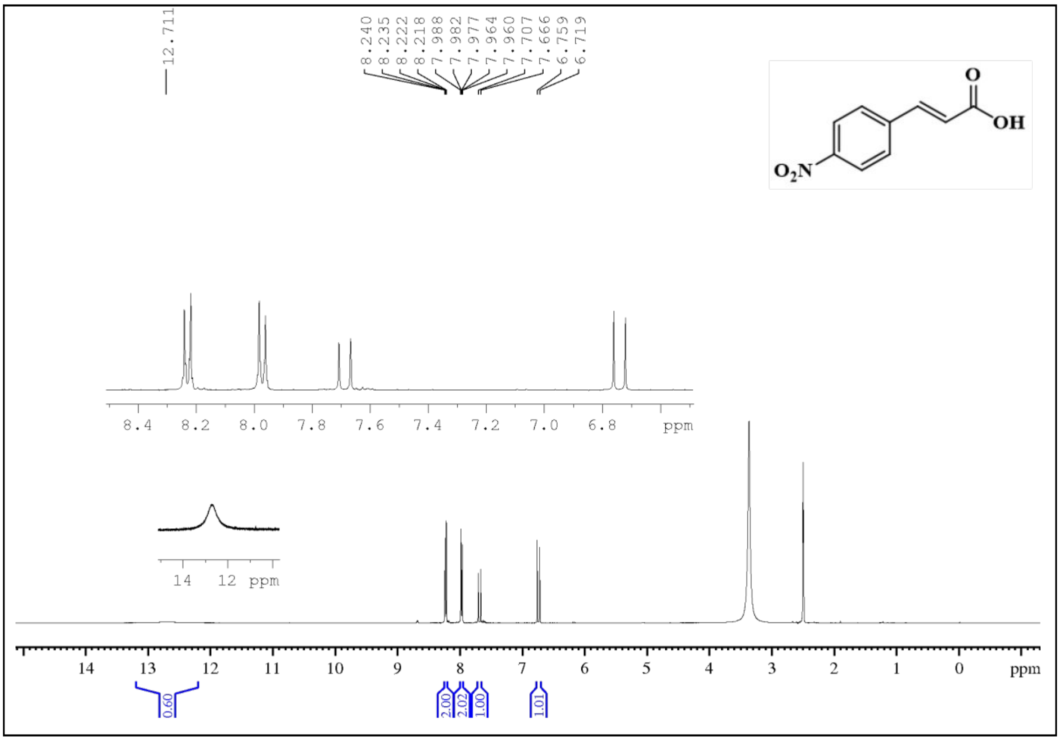

Figure S5. ^{1}H NMR of (*E*)-3-(4-nitrophenyl)acrylic acid

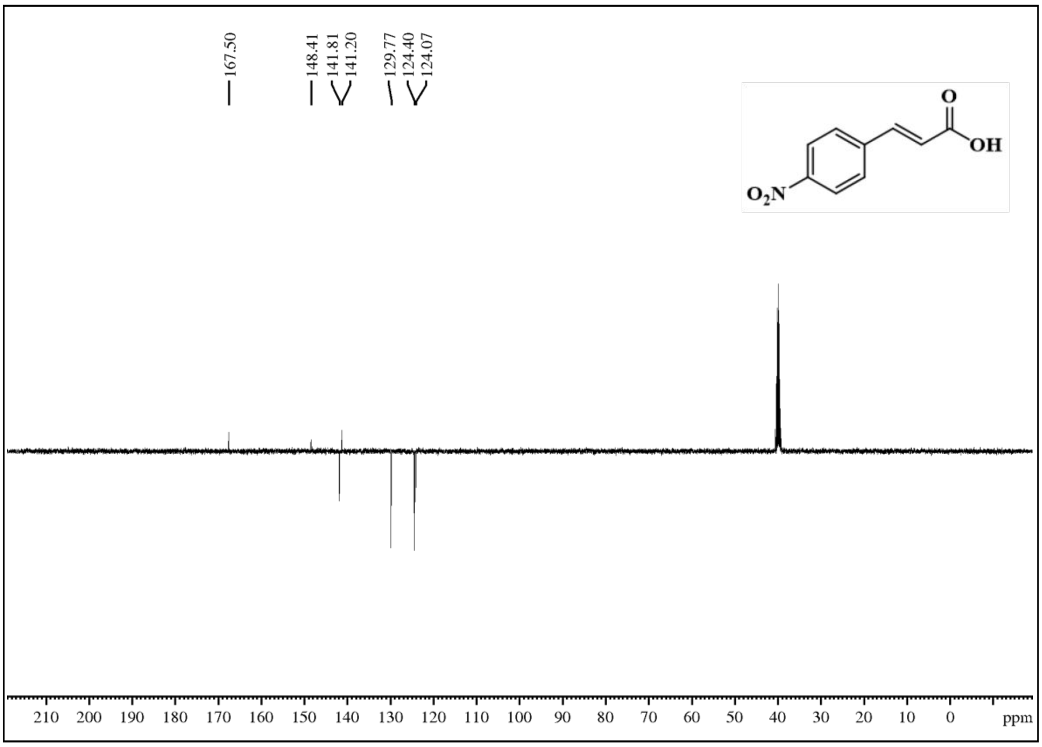

Figure S6. ^{13}C NMR of (*E*)-3-(4-nitrophenyl)acrylic acid

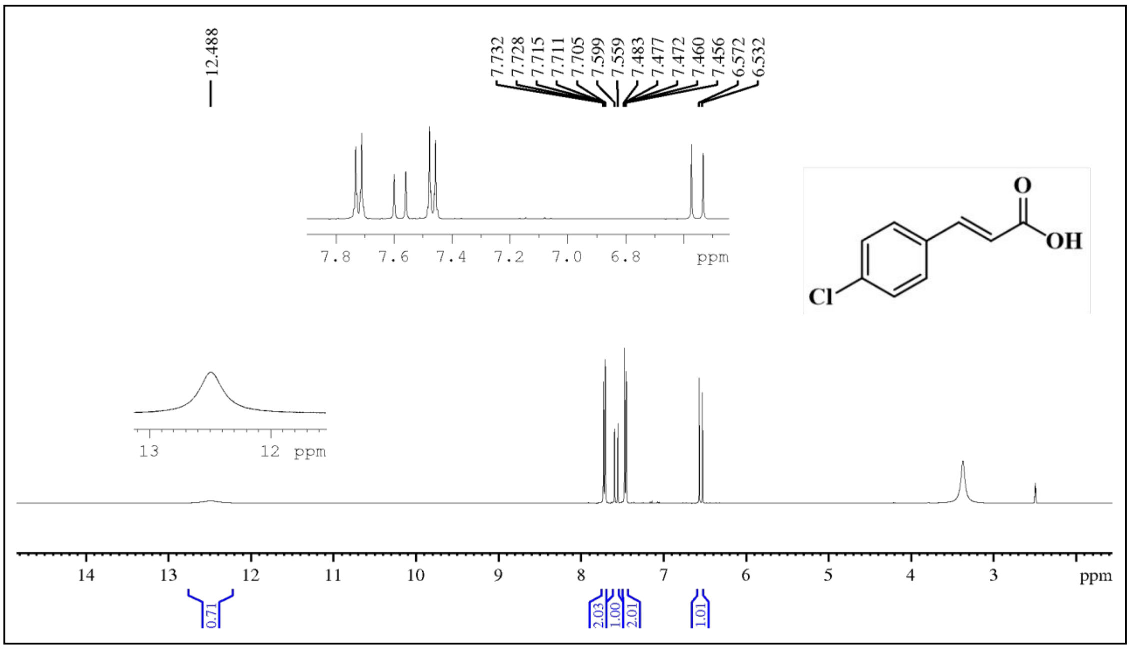

Figure S7. ^{1}H NMR of (*E*)-3-(4-chlorophenyl)acrylic acid

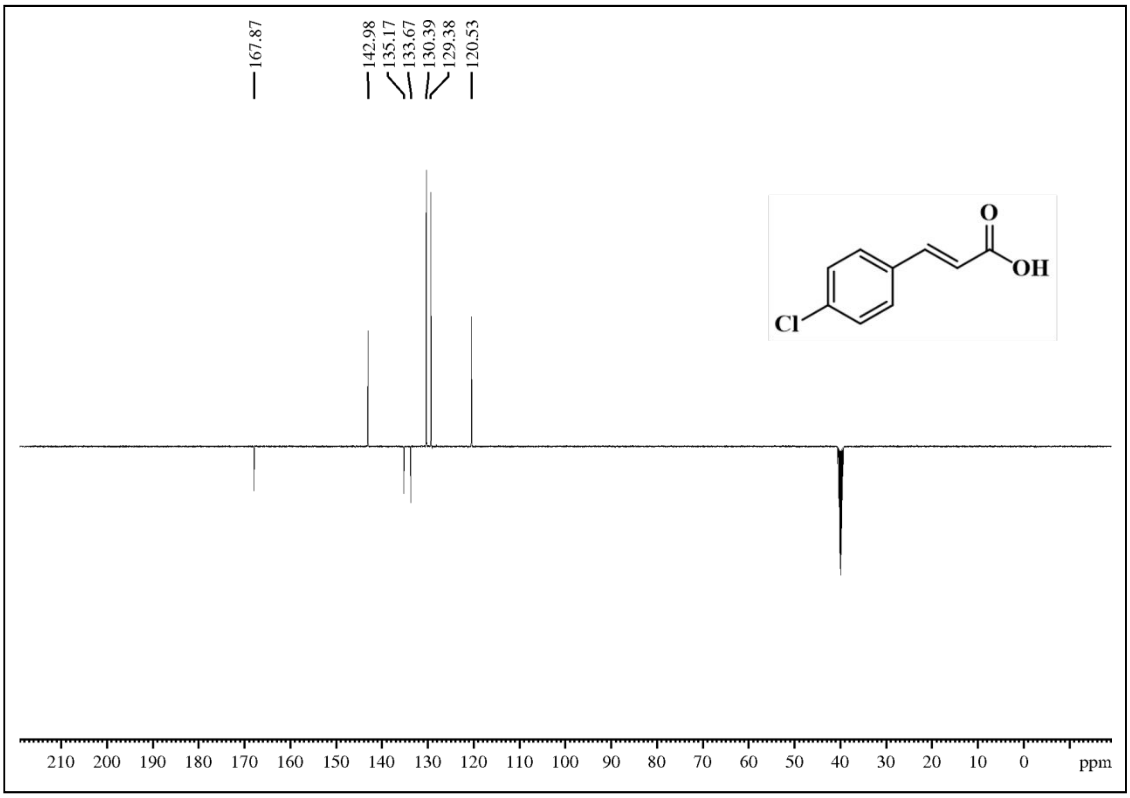

Figure S8. ^{13}C NMR of (*E*)-3-(4-chlorophenyl)acrylic acid

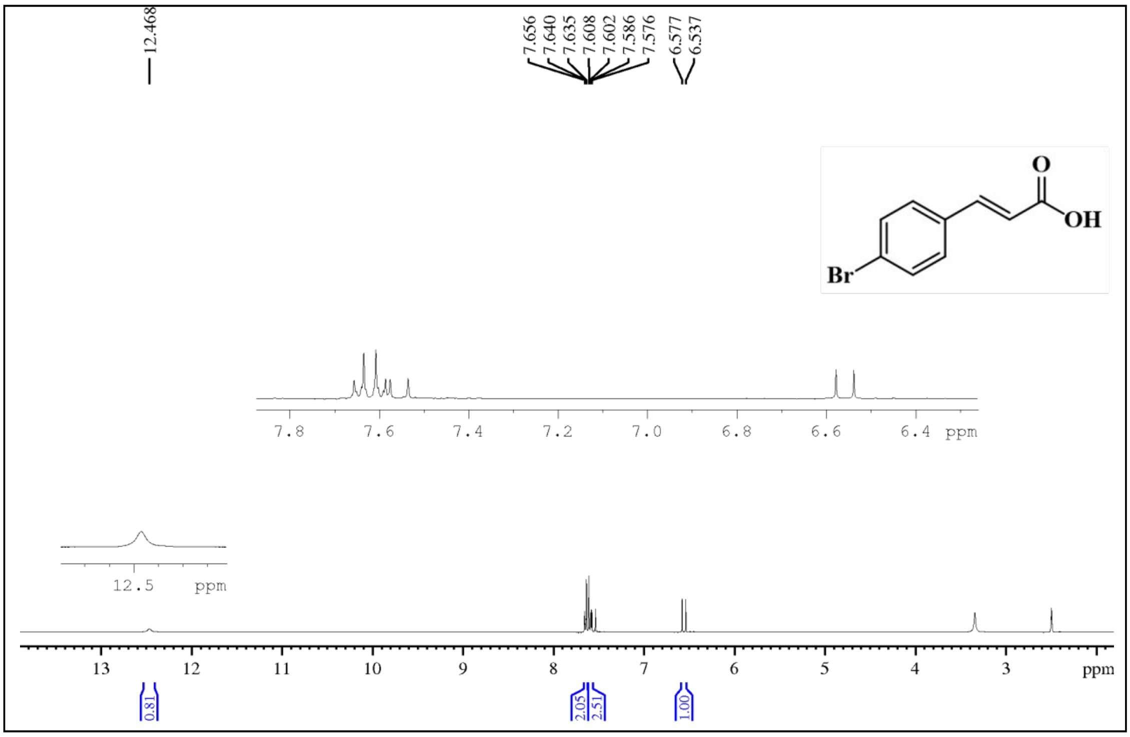

Figure S9. ^{1}H NMR of (*E*)-3-(4-bromophenyl)acrylic acid

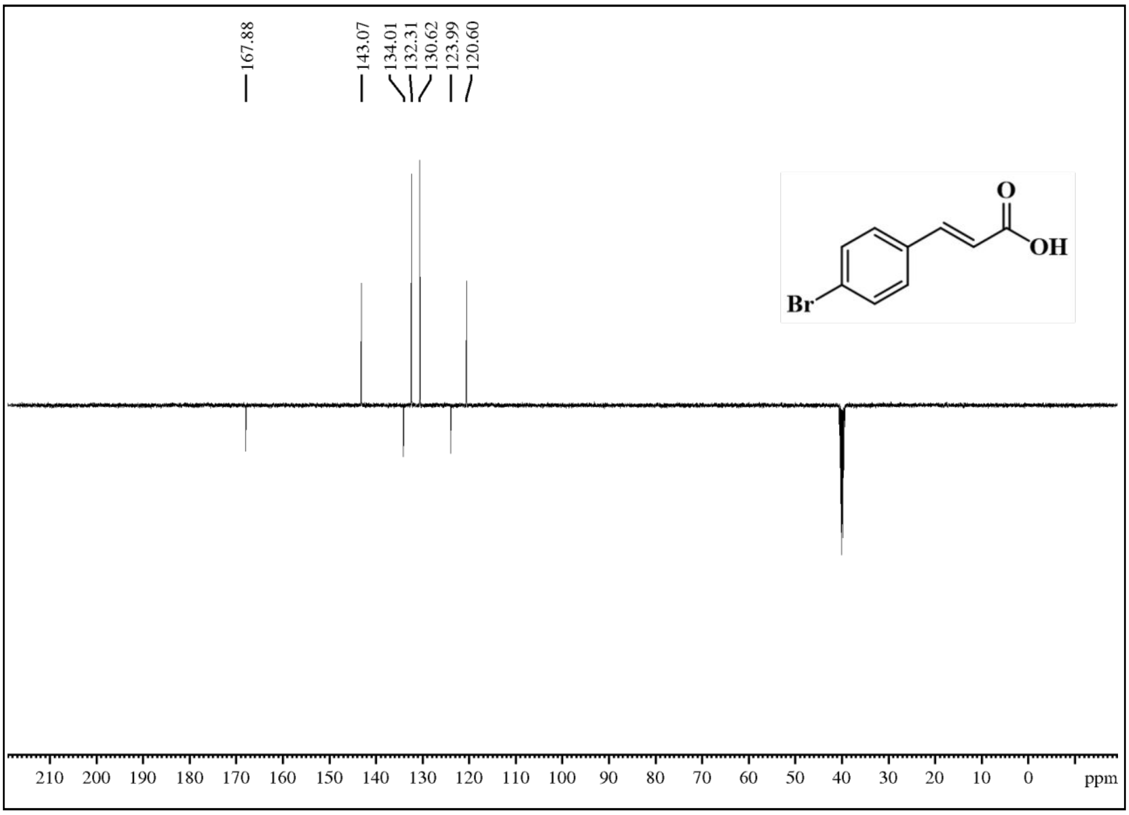

Figure S10. ^{13}C NMR of (*E*)-3-(4-bromophenyl)acrylic acid

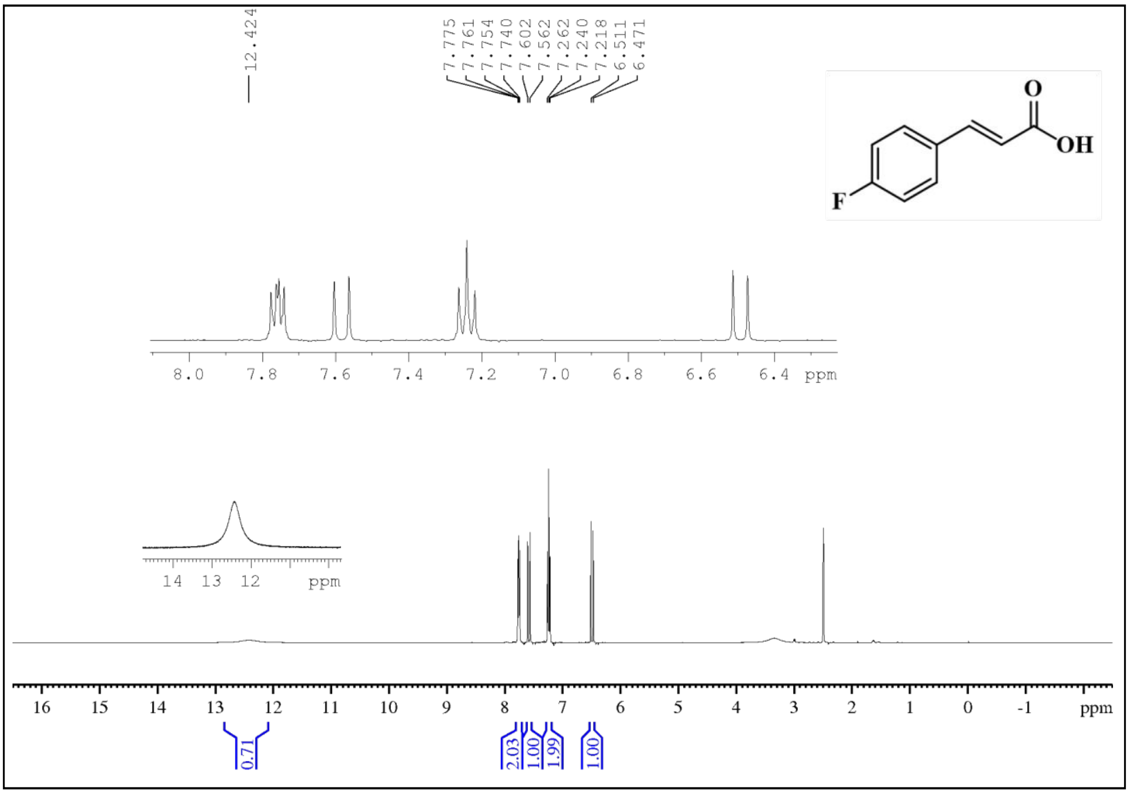

Figura S11. ^{1}H NMR of (*E*)-3-(4-fluorophenyl)acrylic acid

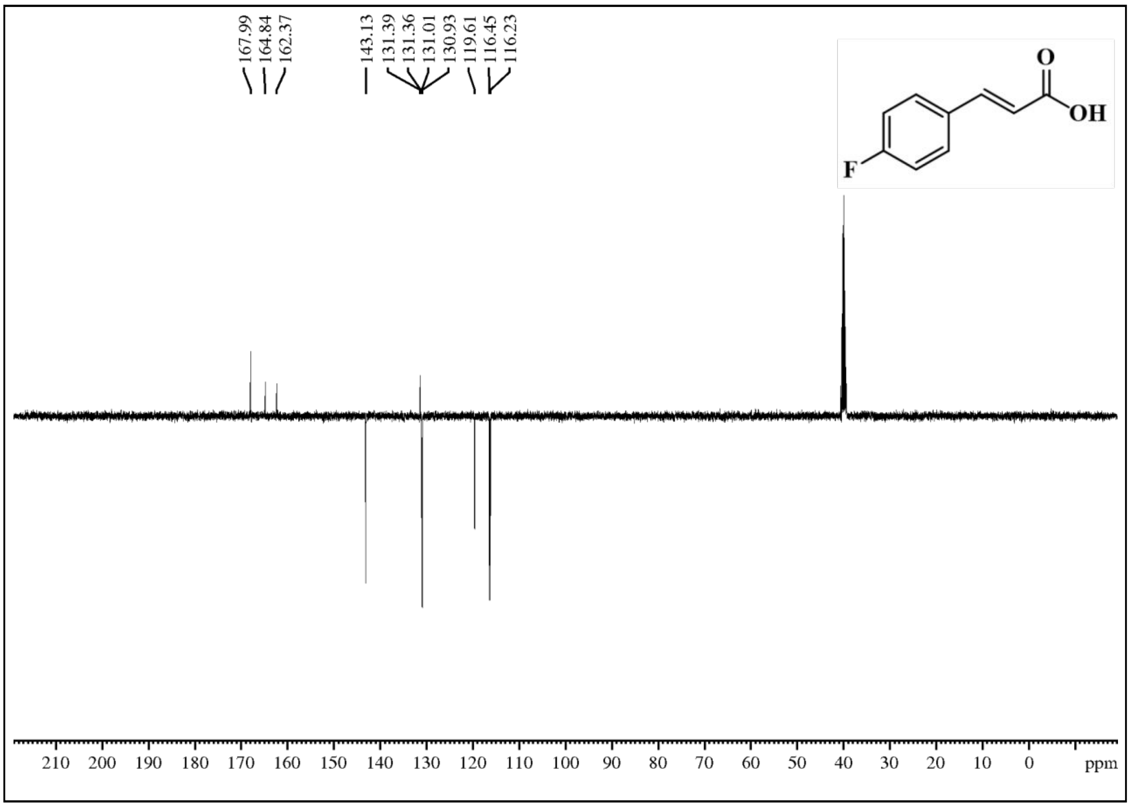

Figura S12. ^{13}C NMR of (*E*)-3-(4-fluorophenyl)acrylic acid

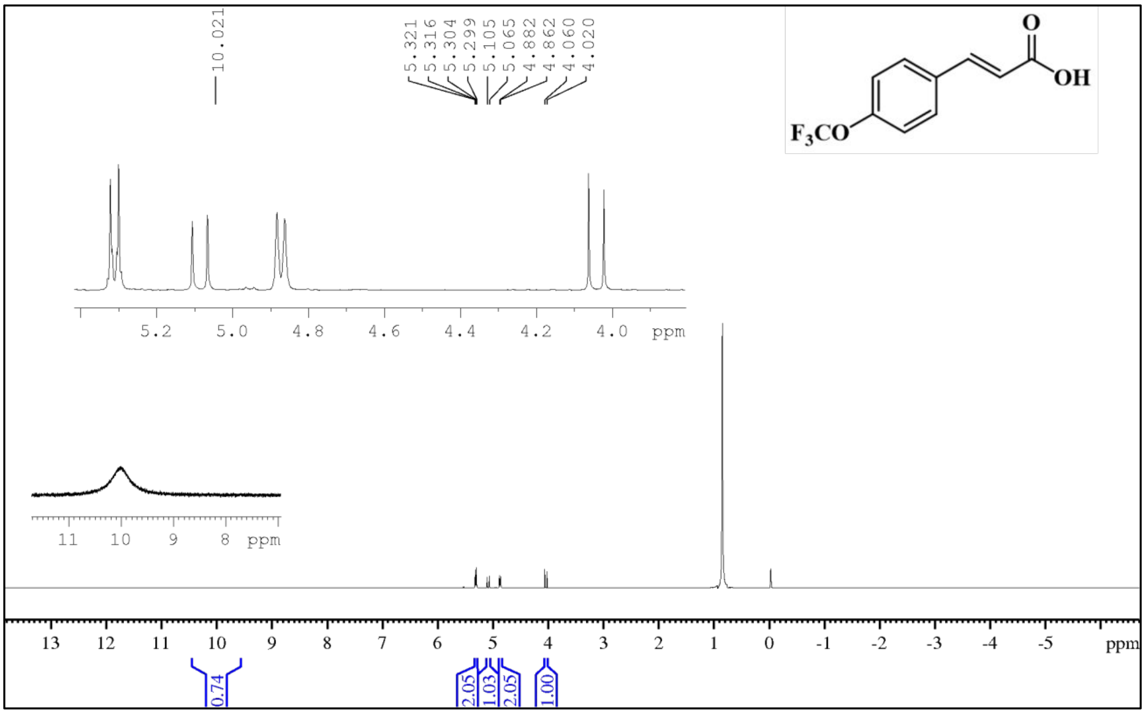

Figure S13. ^{1}H NMR of (*E*)-3-(4-(trifluoromethoxy)phenyl)acrylic acid

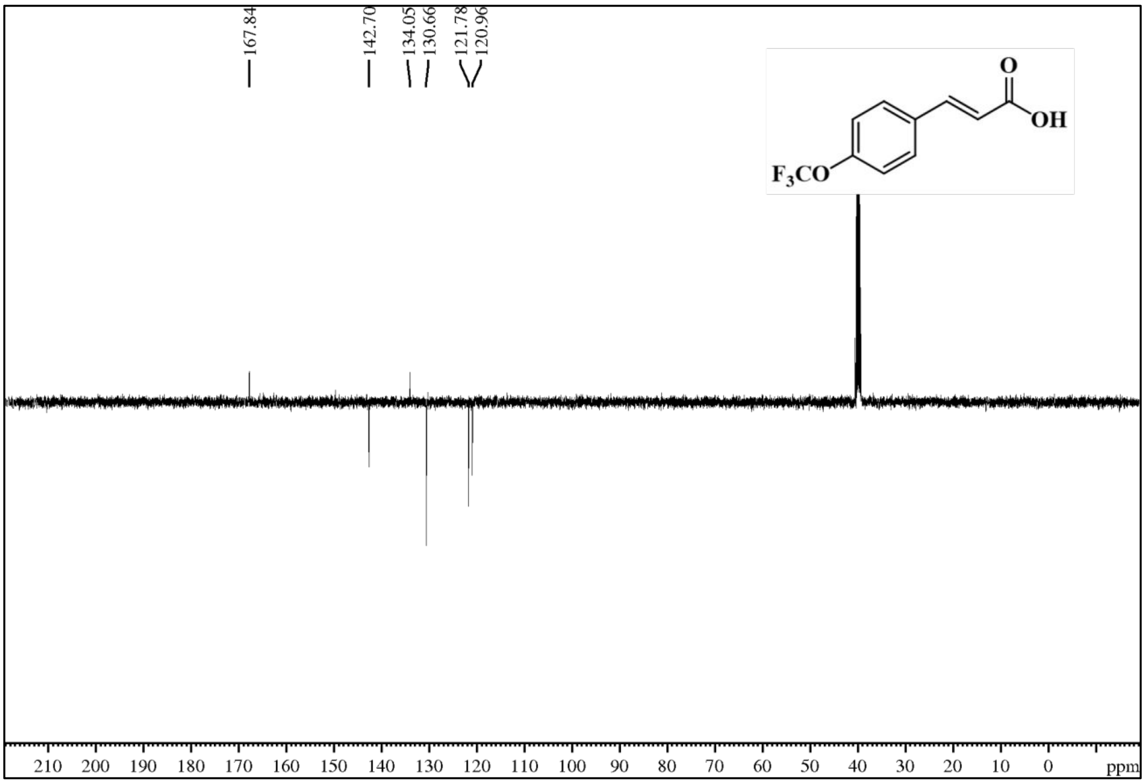

Figure S14. ^{13}C NMR of (*E*)-3-(4-(trifluoromethoxy)phenyl)acrylic acid

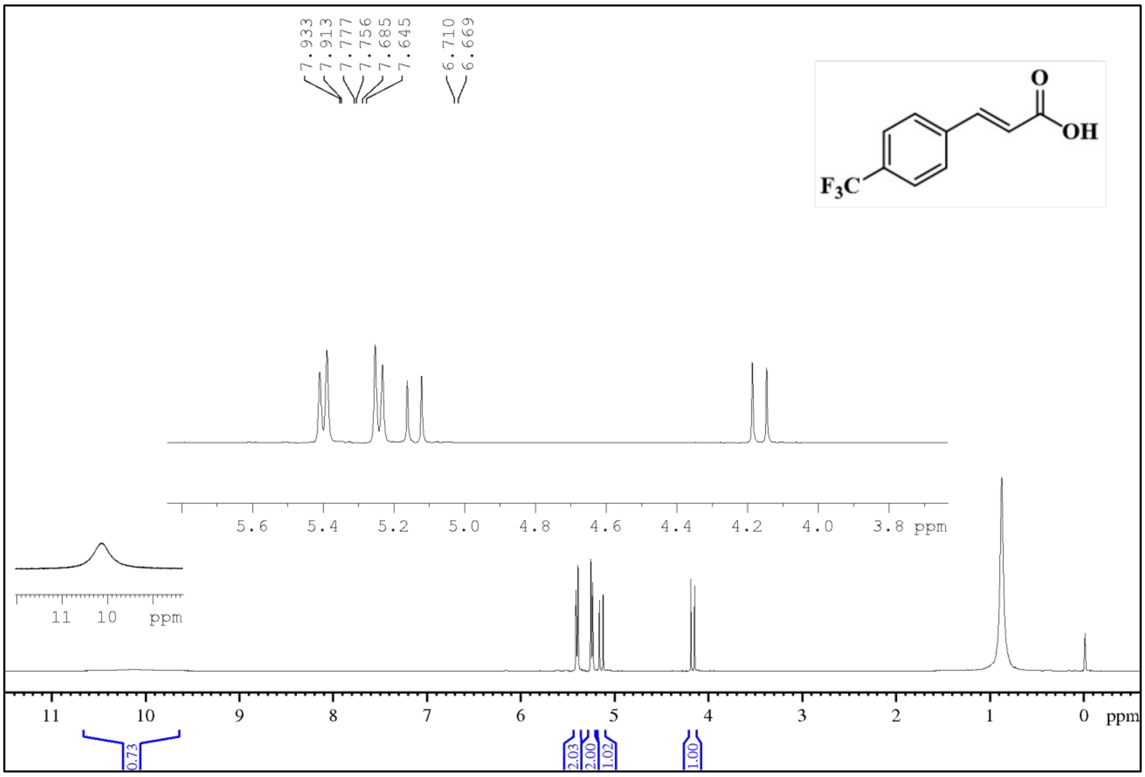

Figure S15. ^{1}H NMR of (*E*)-3-(4-(trifluoromethyl)phenyl)acrylic acid

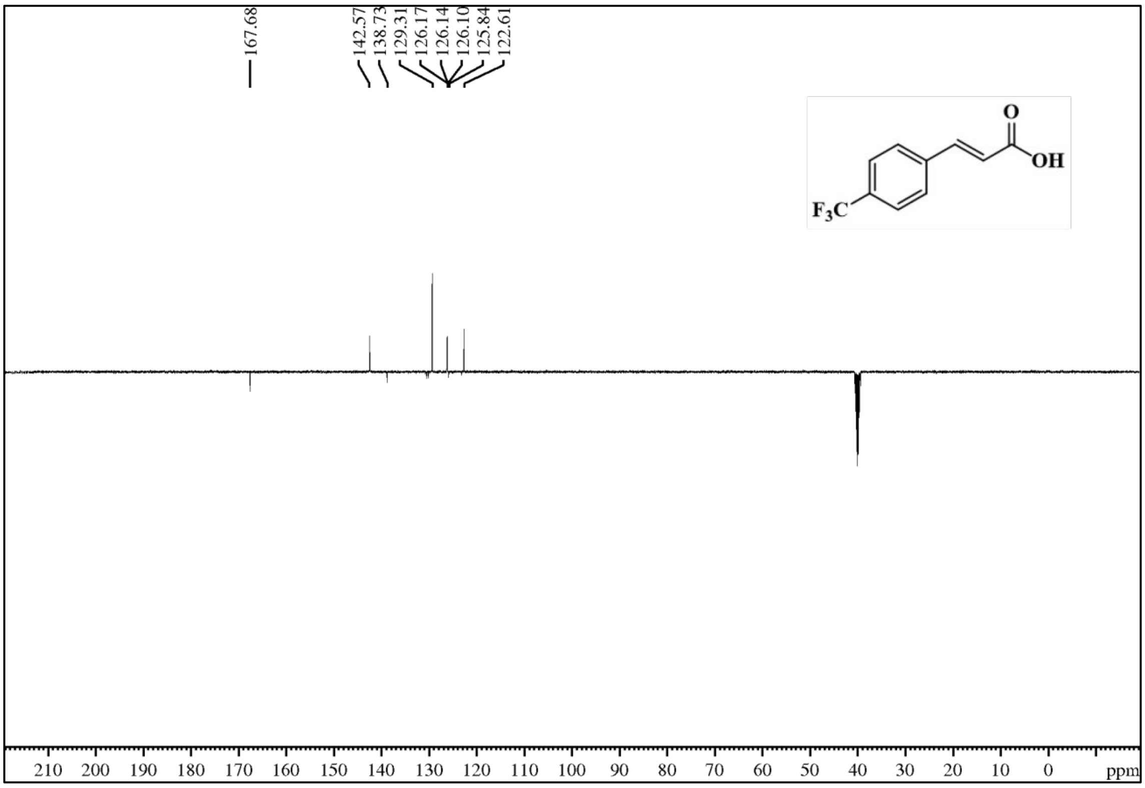

Figure S16. ^{13}C NMR of (*E*)-3-(4-(trifluoromethyl)phenyl)acrylic acid

4. FTIR spectra of cinnamic acids

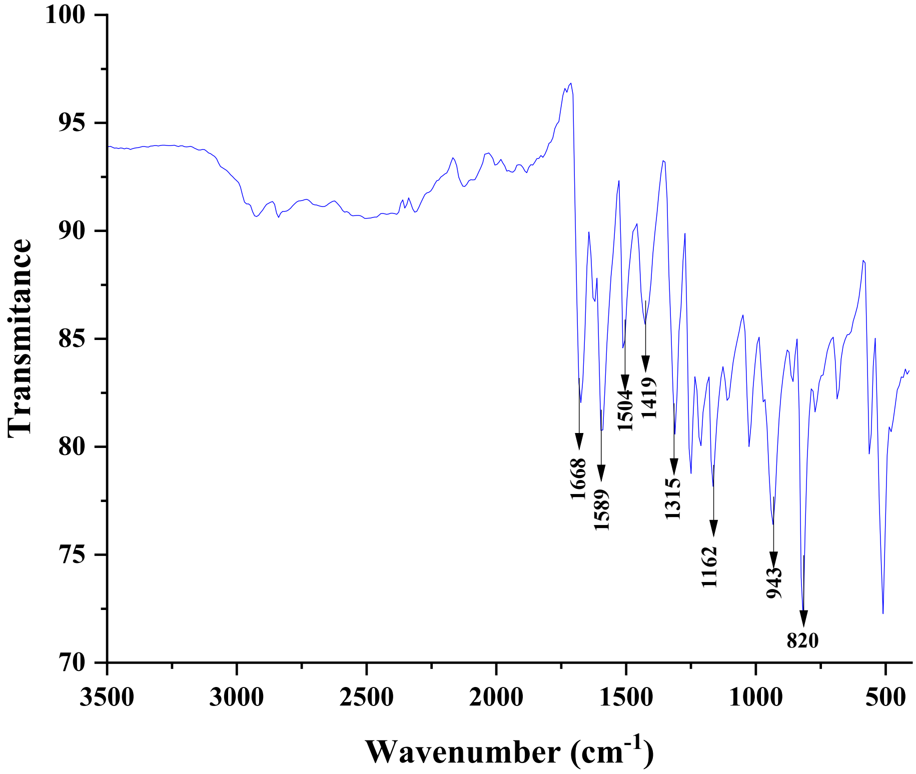

Figure S17. FT-IR of (*E*)-3-(4-(trifluoromethoxy)phenyl)acrylic acid

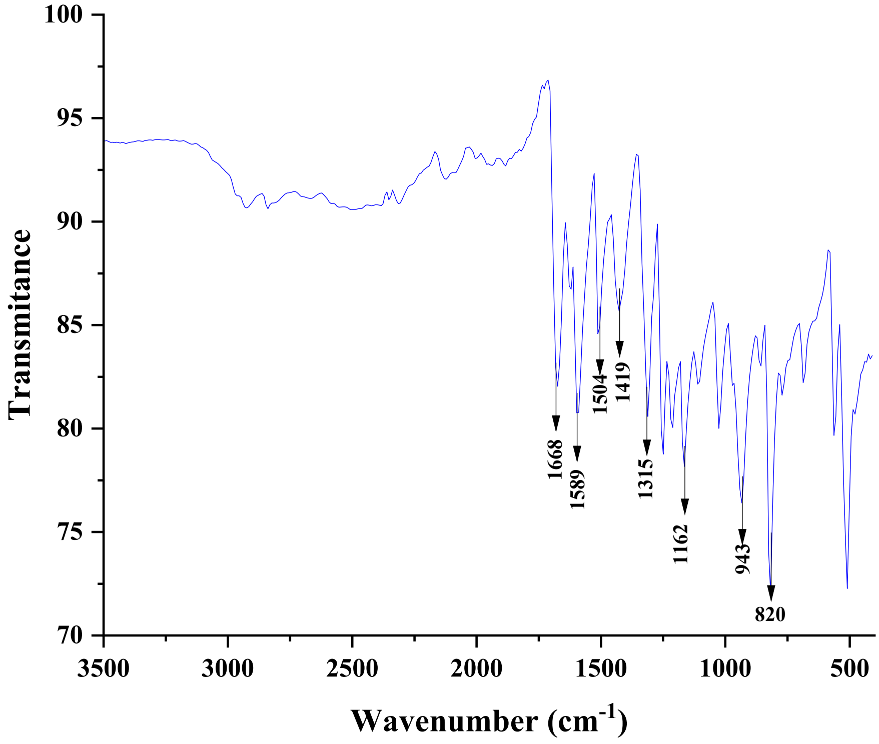

Figure S18. FT-IR of (*E*)-3-(4-(trifluoromethoxy)phenyl)acrylic acid

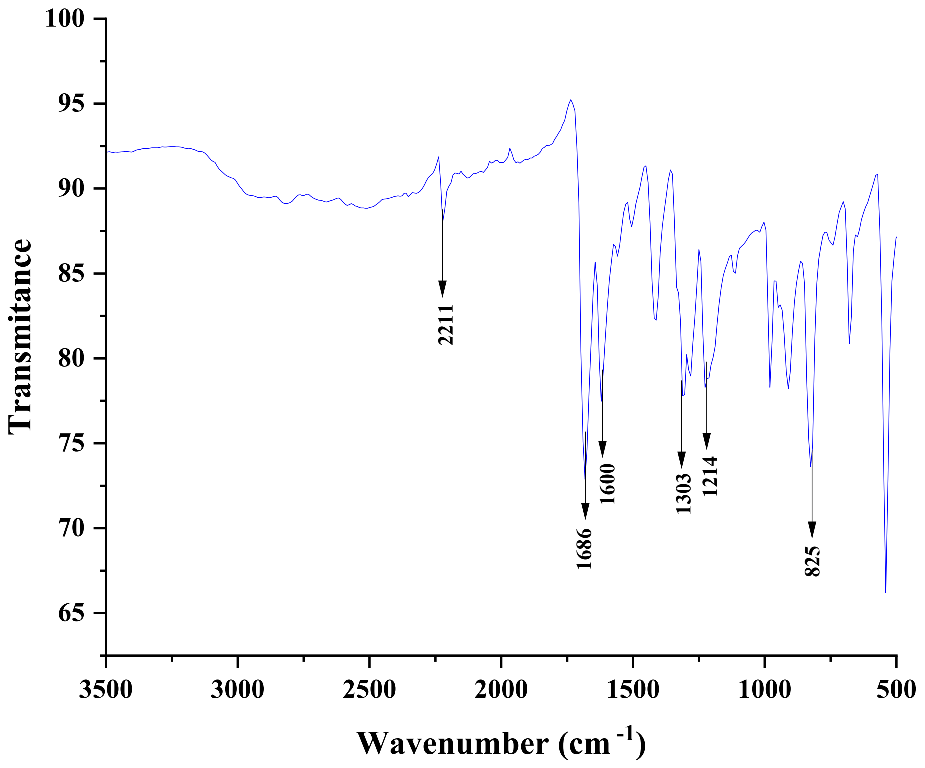

Figure S19. FT-IR of (*E*)-3-(4-cyanophenyl)acrylic acid

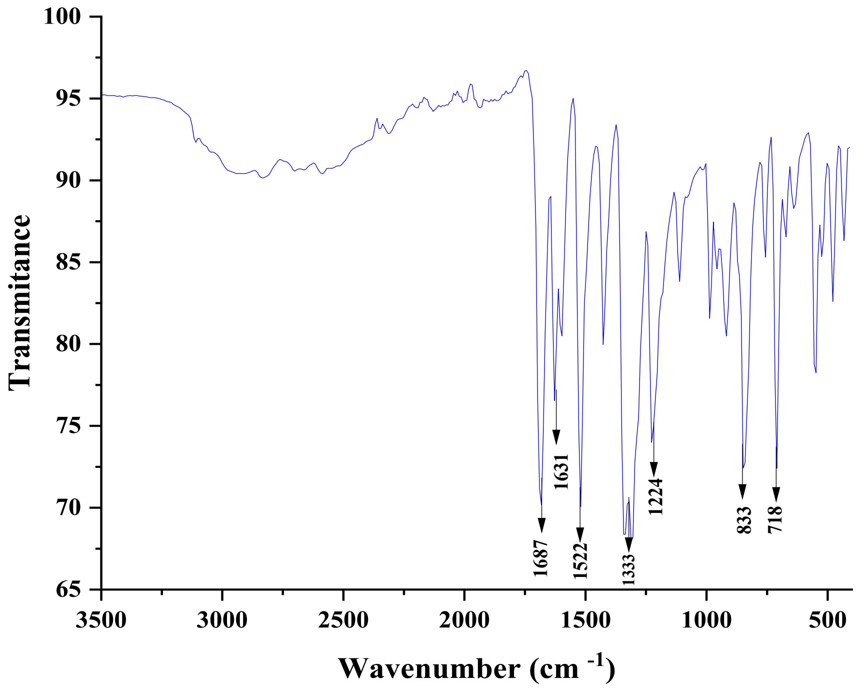

Figure S20. FT-IR of (*E*)-3-(4-nitrophenyl)acrylic acid

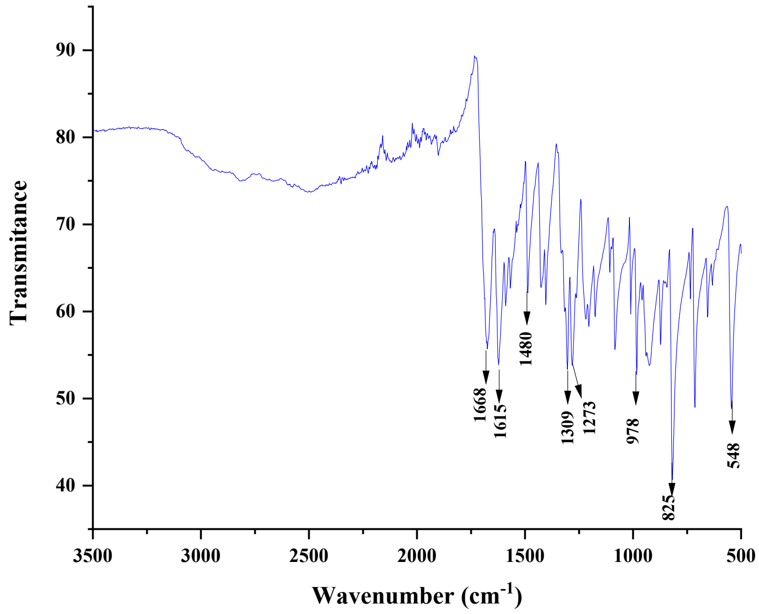

Figure S21. FT-IR of (*E*)-3-(4-chlorophenyl)acrylic acid

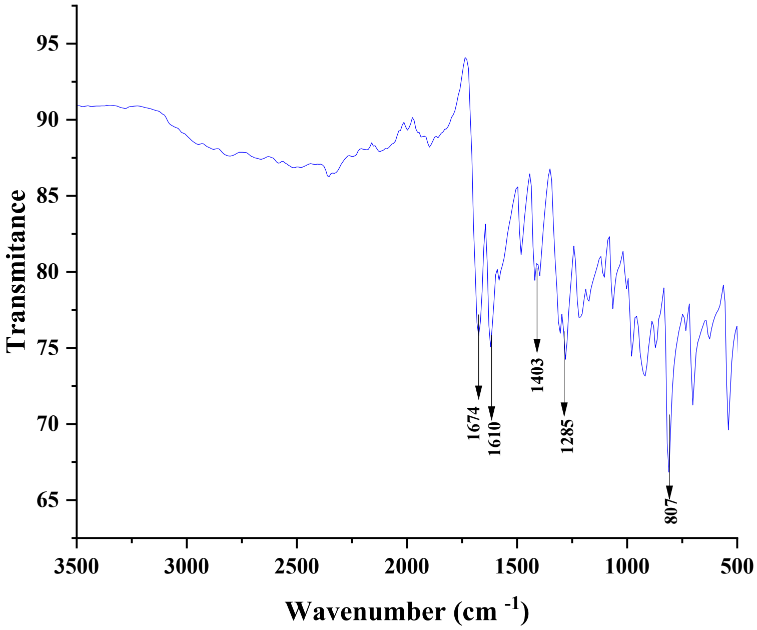

Figure S22. FT-IR of (*E*)-3-(4-bromophenyl)acrylic acid

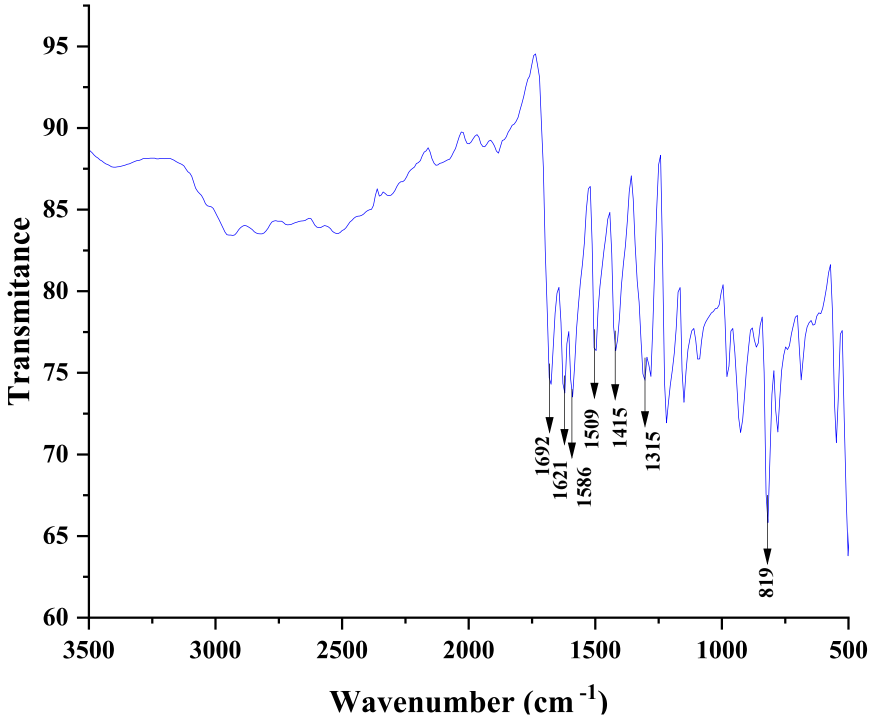

Figure S23. FT-IR of (*E*)-3-(4-fluorophenyl)acrylic acid

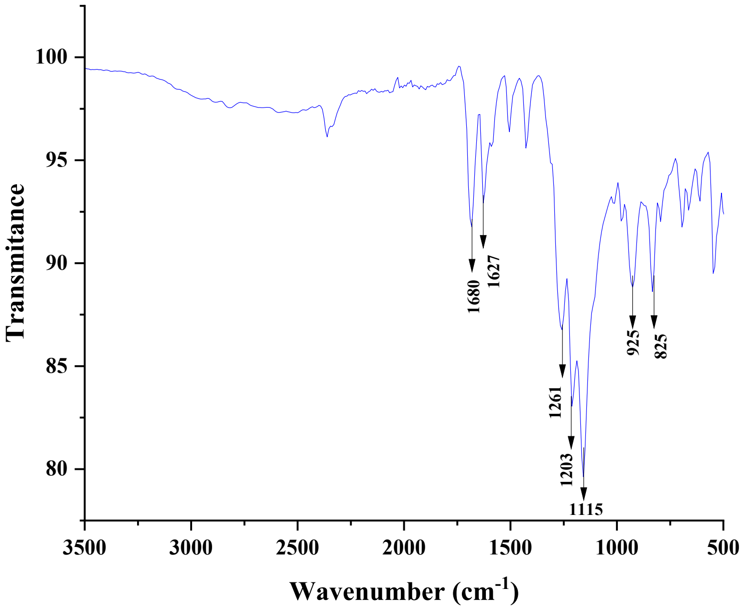

Figure S24. FT-IR of (*E*)-3-(4-(trifluoromethoxy)phenyl)acrylic acid

5. ^{1}H NMR and DEPT ^{13}C spectra of cinnamamides

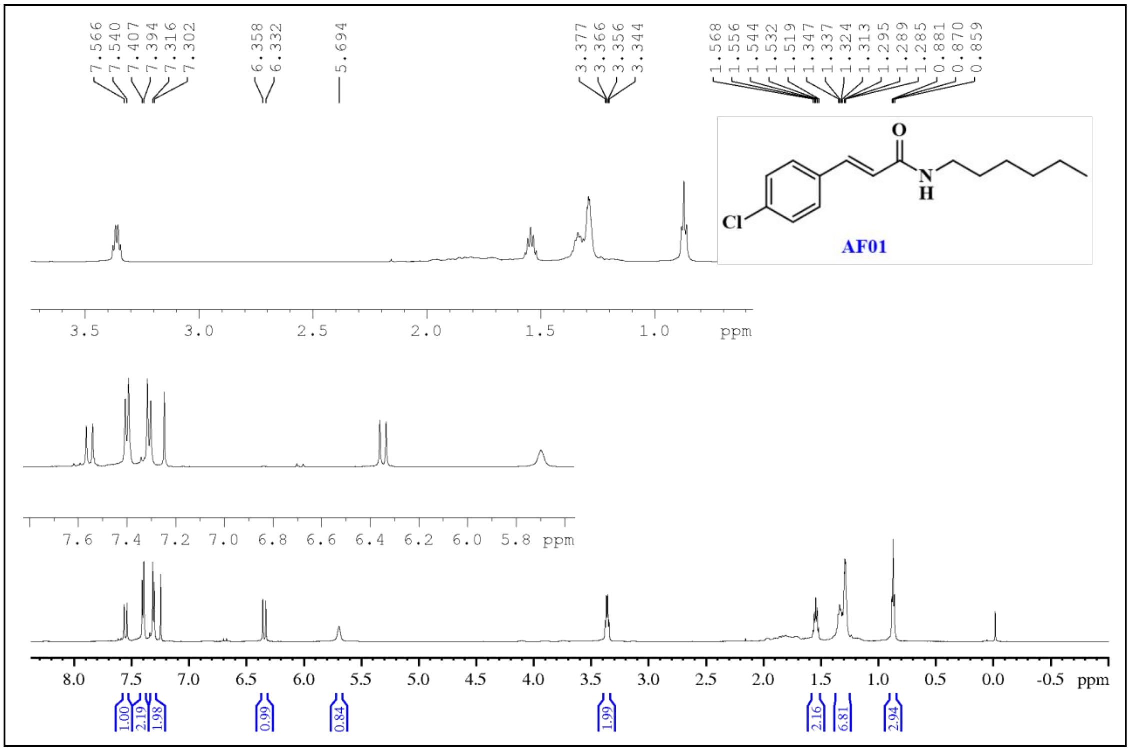

Figure S25. ^{1}H NMR spectrum of (*E*)-3-(4-chlorophenyl)-*N*-hexylacrylamide (600 MHz, $CDCl_3$) (**AF01**)

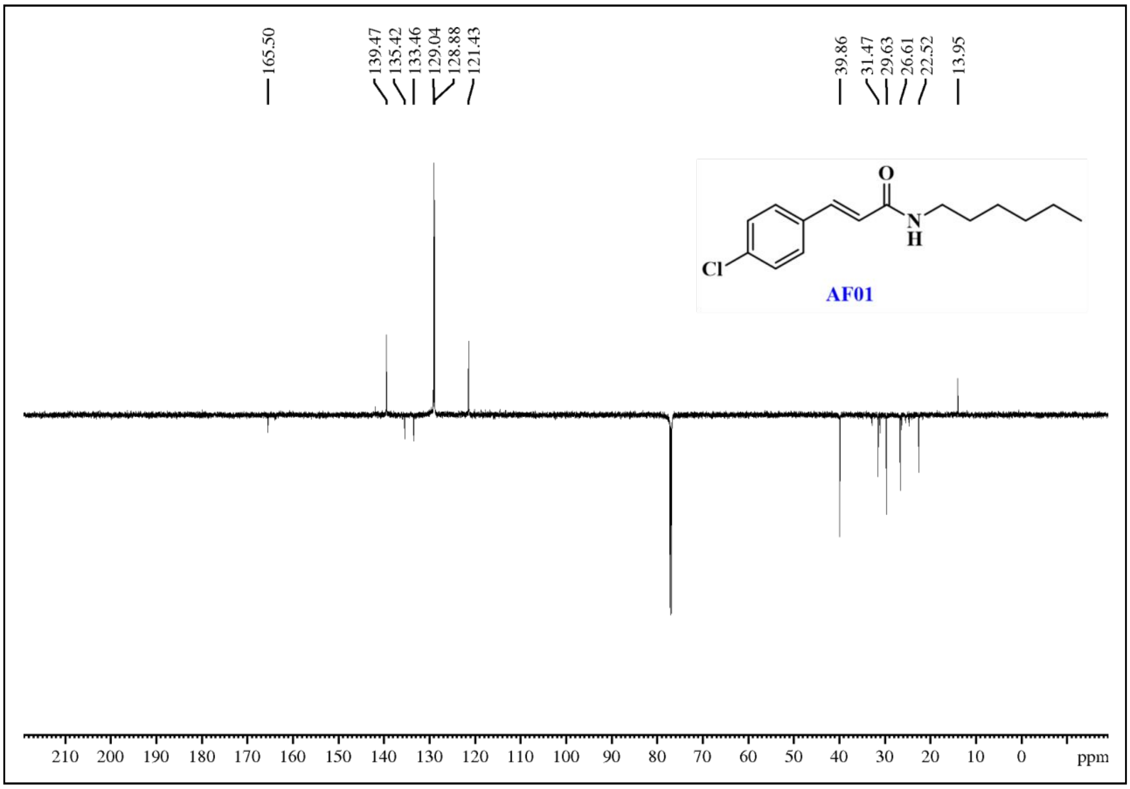

Figure S26. ^{13}C NMR spectrum of (*E*)-3-(4-chlorophenyl)-*N*-hexylacrylamide (150 MHz, $CDCl_3$) (**AF01**)

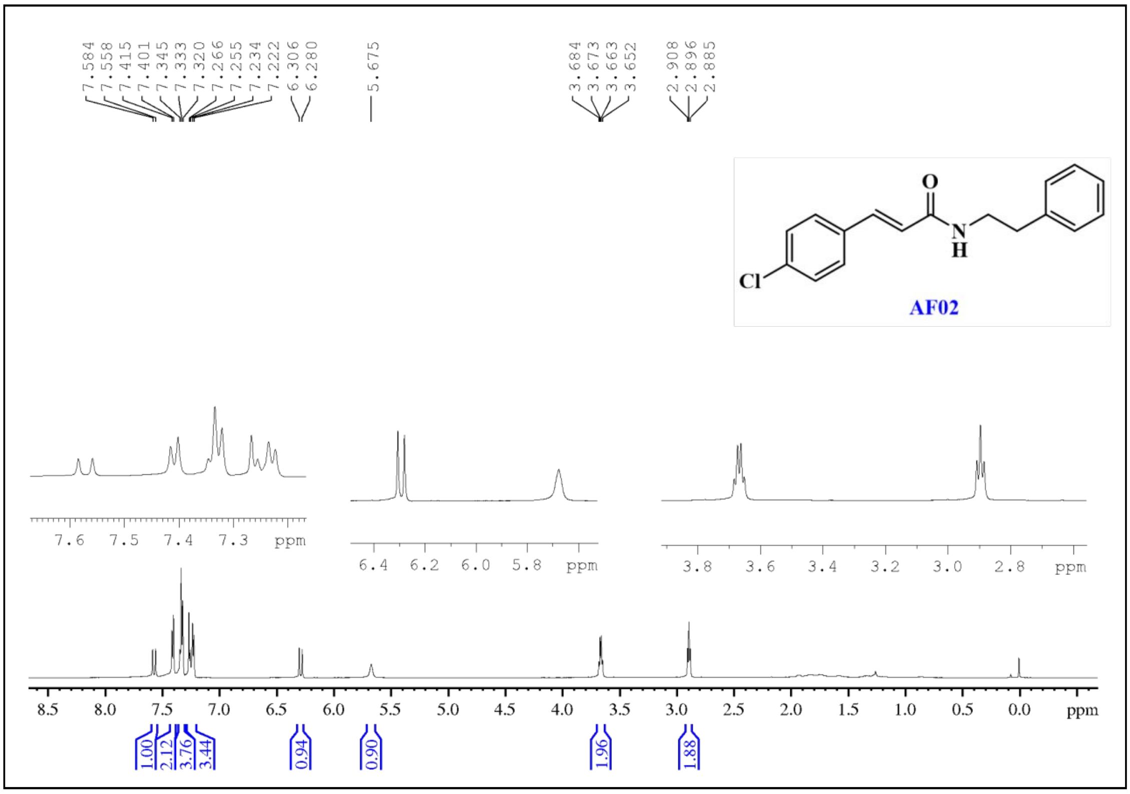

Figure S27. ^{1}H NMR spectrum of (*E*)-3-(4-chlorophenyl)-*N*-phenethylacrylamide (600 MHz, $CDCl_3$) (**AF02**)

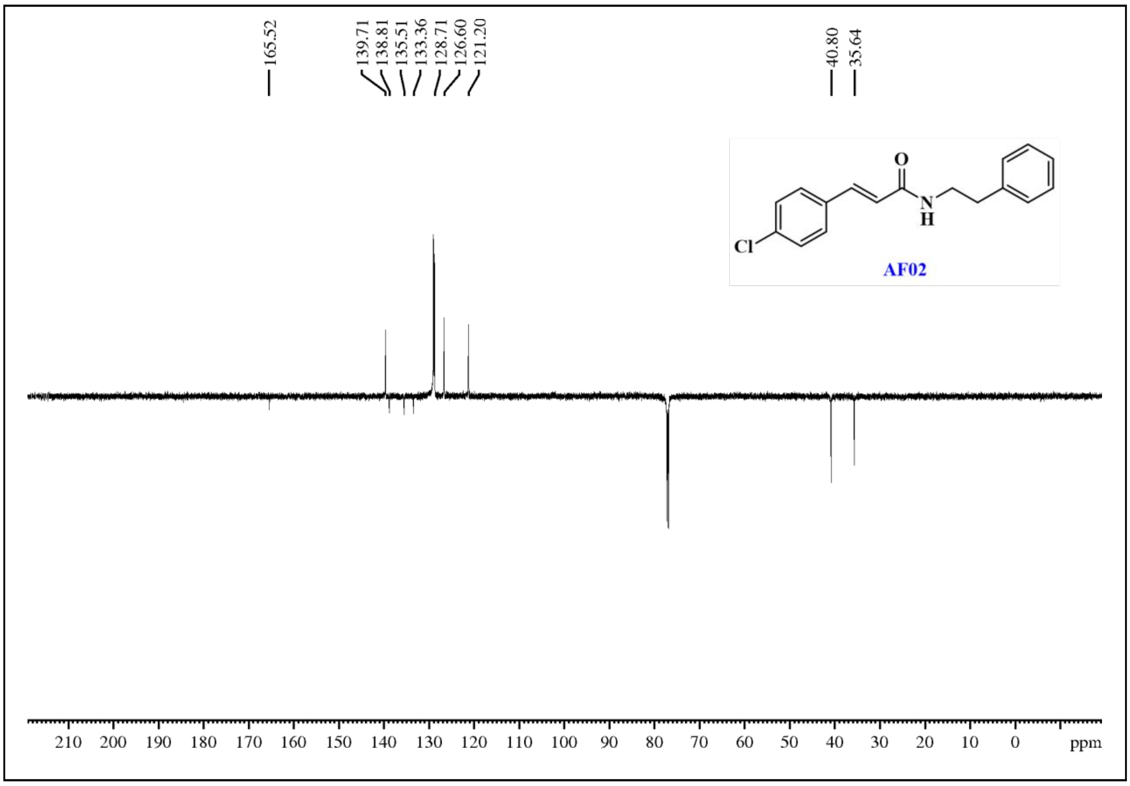

Figure S28. ^{13}C NMR spectrum of (*E*)-3-(4-chlorophenyl)-*N*-phenethylacrylamide (150 MHz, $CDCl_3$) (**AF02**)

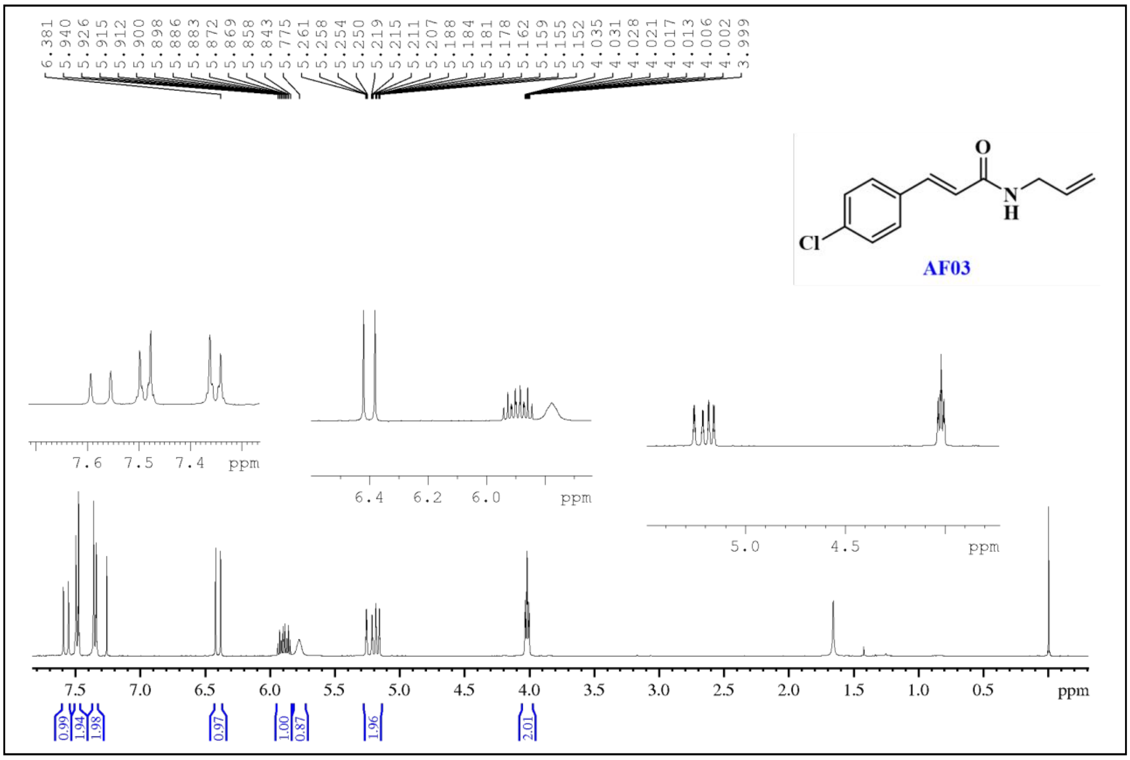

Figure S29. ^{1}H NMR spectrum of (*E*)-*N*-allyl-3-(4-chlorophenyl)acrylamide (400 MHz, $CDCl_3$) (**AF03**)

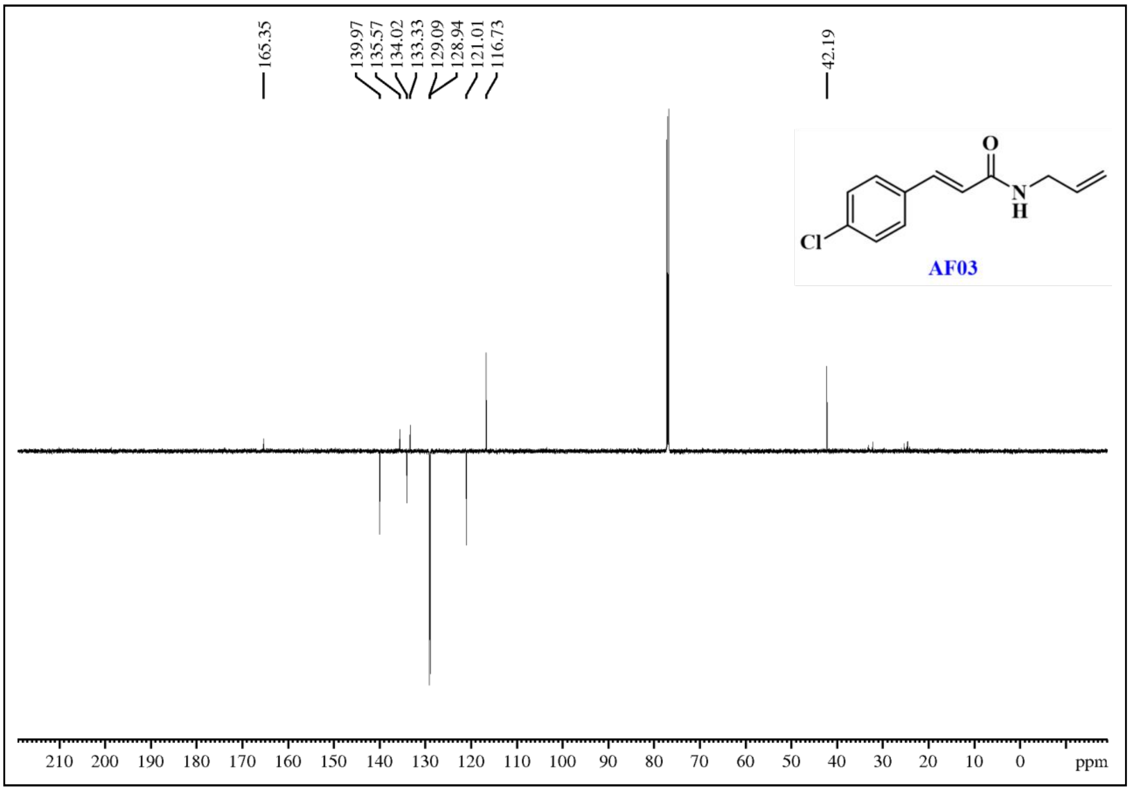

Figure S30. ^{13}C NMR spectrum of (*E*)-*N*-allyl-3-(4-chlorophenyl)acrylamide (100 MHz, $CDCl_3$) (**AF03**)

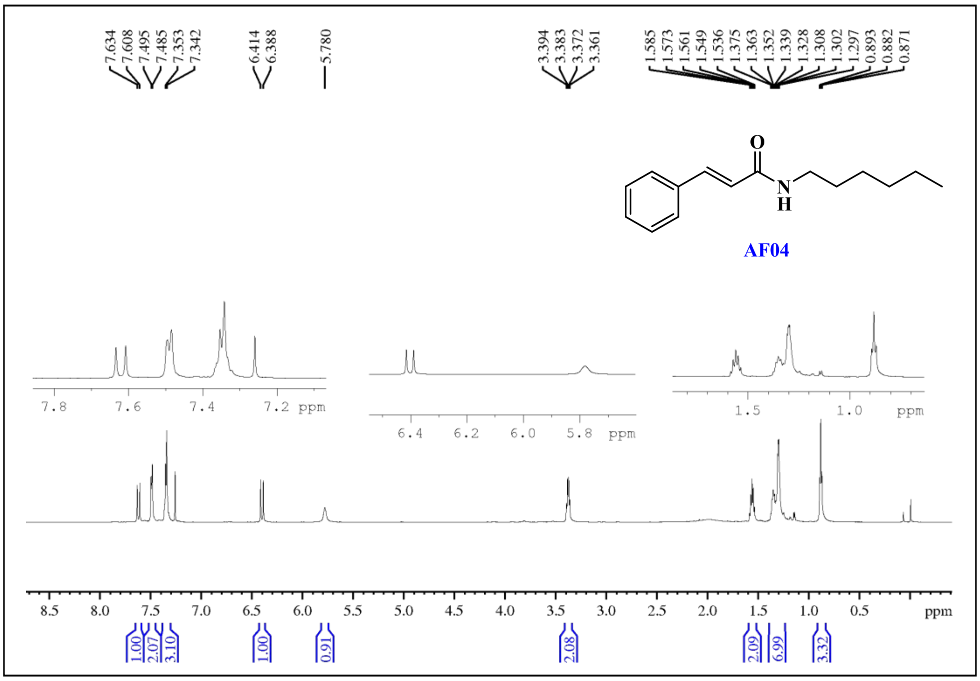

Figure S31. ^{1}H NMR spectrum of ***N*-hexylcinnamamide** (600 MHz, $CDCl_3$) (**AF04**)

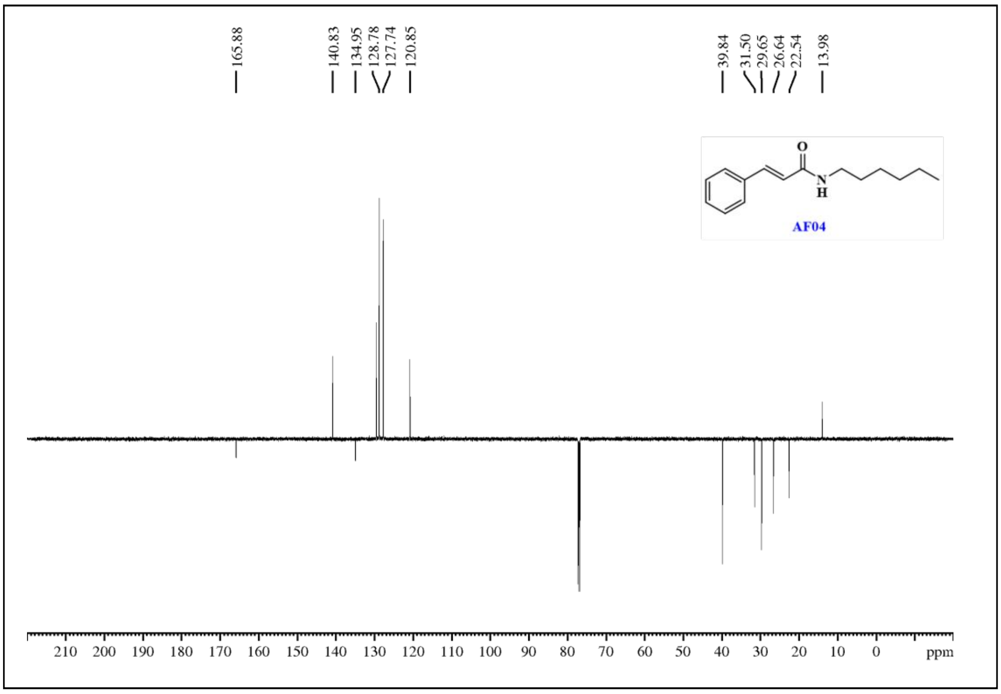

Figure S32. ^{13}C NMR spectrum of *N*-hexylcinnamamide (150 MHz, $CDCl_3$) (**AF04**)

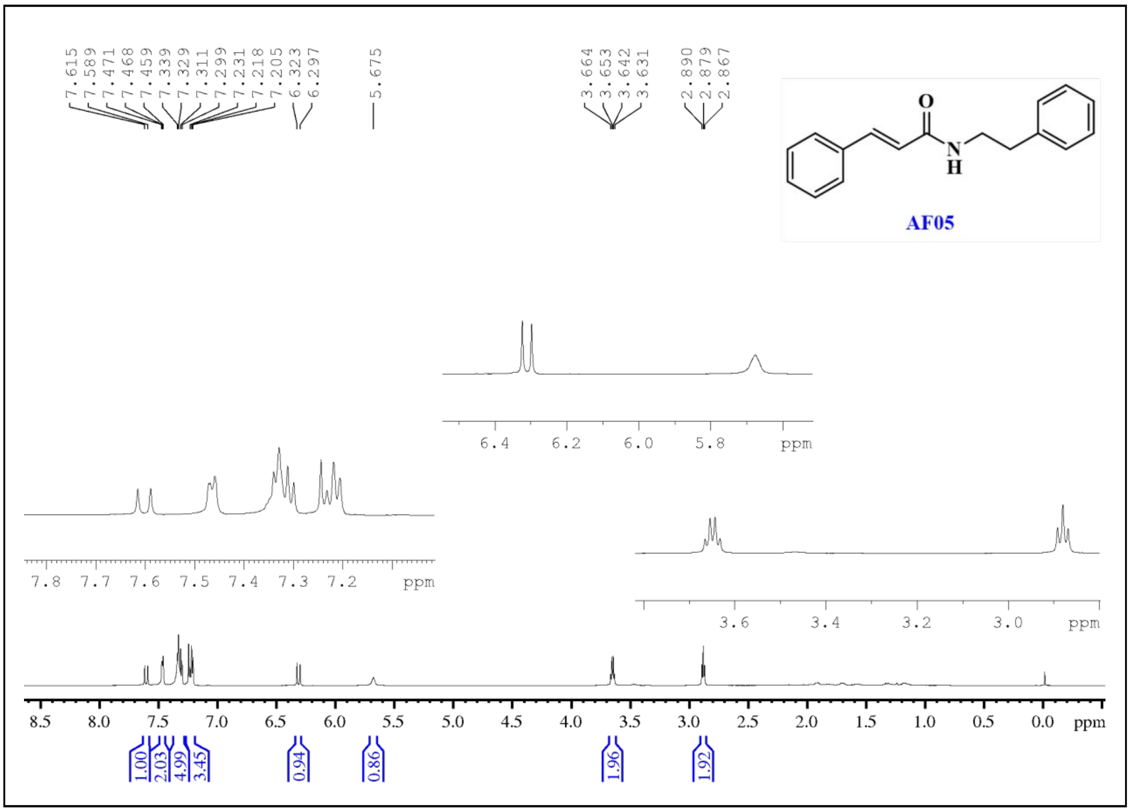

Figure S33. ^{1}H NMR spectrum of (*E*)-*N*-phenethylcinnamamide (600 MHz, $CDCl_3$) (**AF05**)

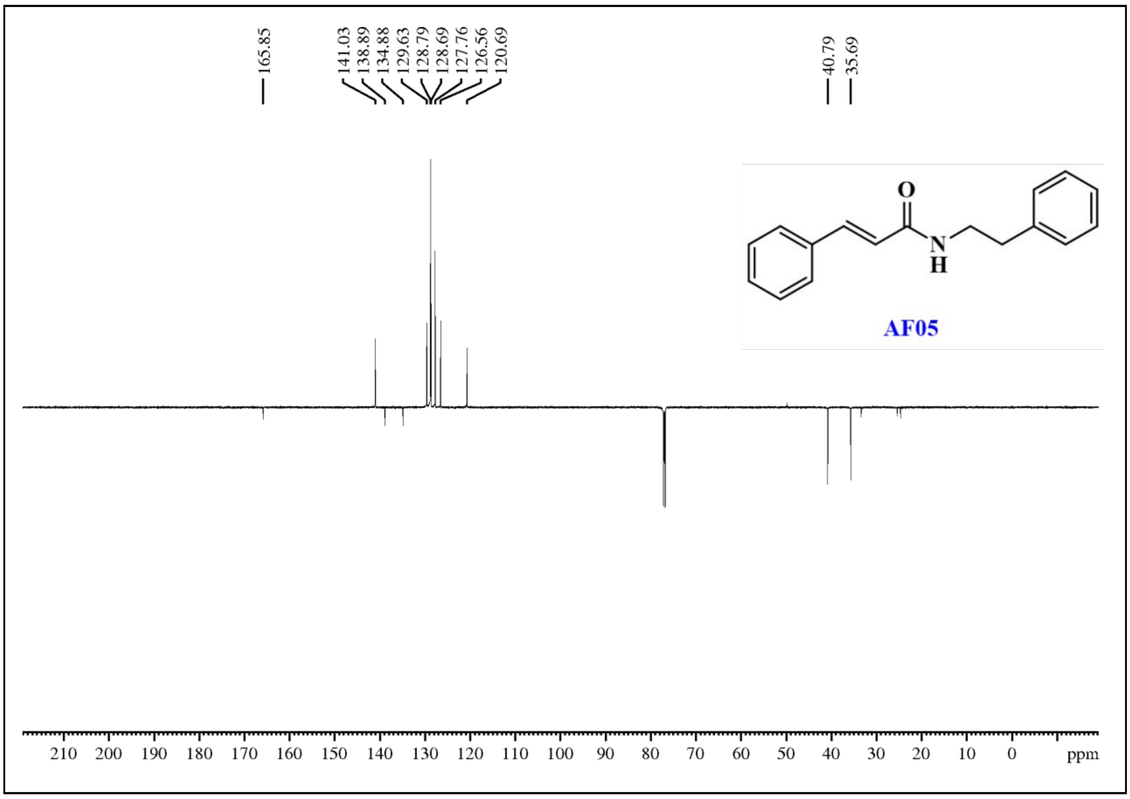

Figure S34. ^{13}C NMR spectrum of (*E*)-*N*-phenethylcinnamamide ((150 MHz, $CDCl_3$) (**AF05**)

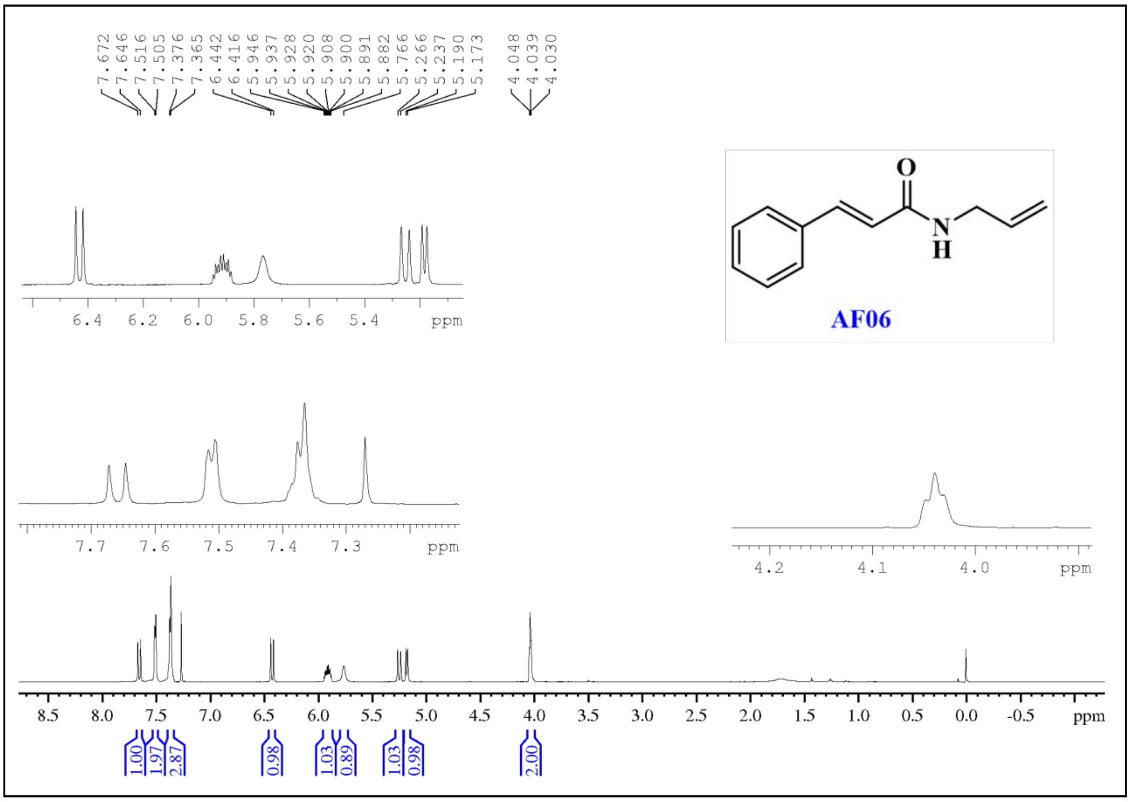

Figure S35. ^{1}H NMR spectrum of N-allylcinnamamide (600 MHz, $CDCl_3$) (**AF06**)

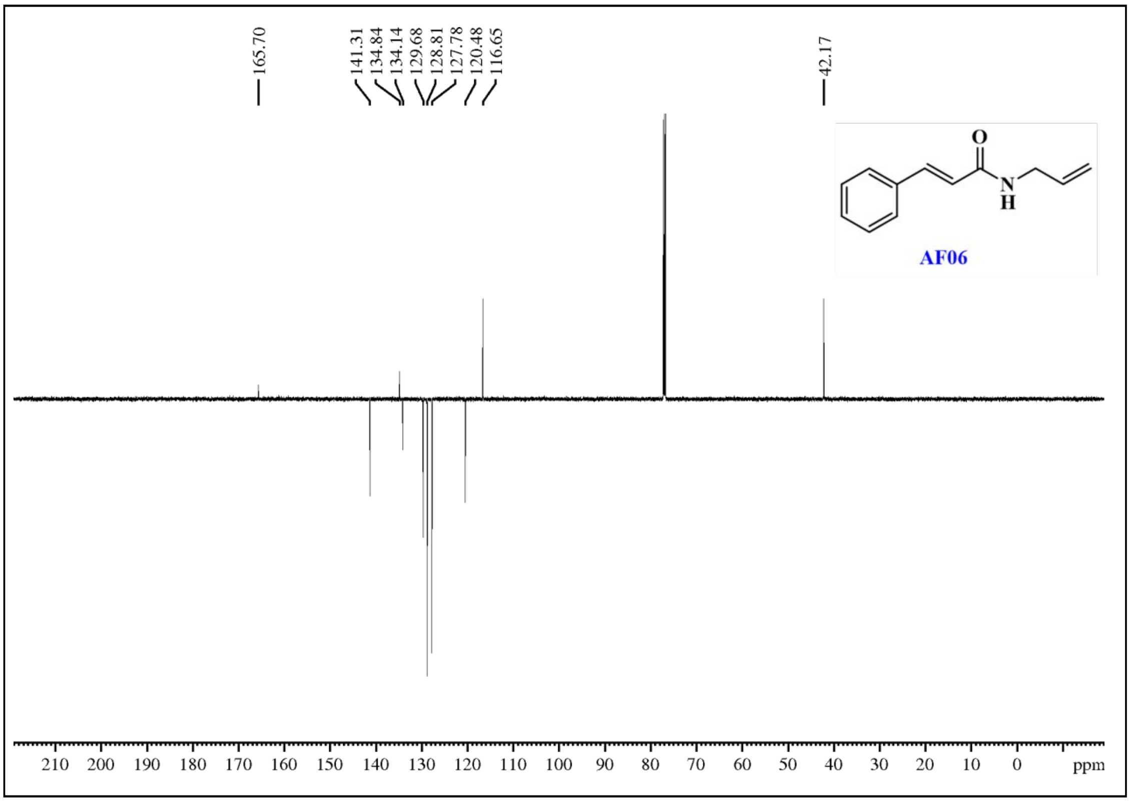

Figure S36. ^{13}C NMR spectrum of *N*-allylcinnamamide (150 MHz, $CDCl_3$) (**AF06**)

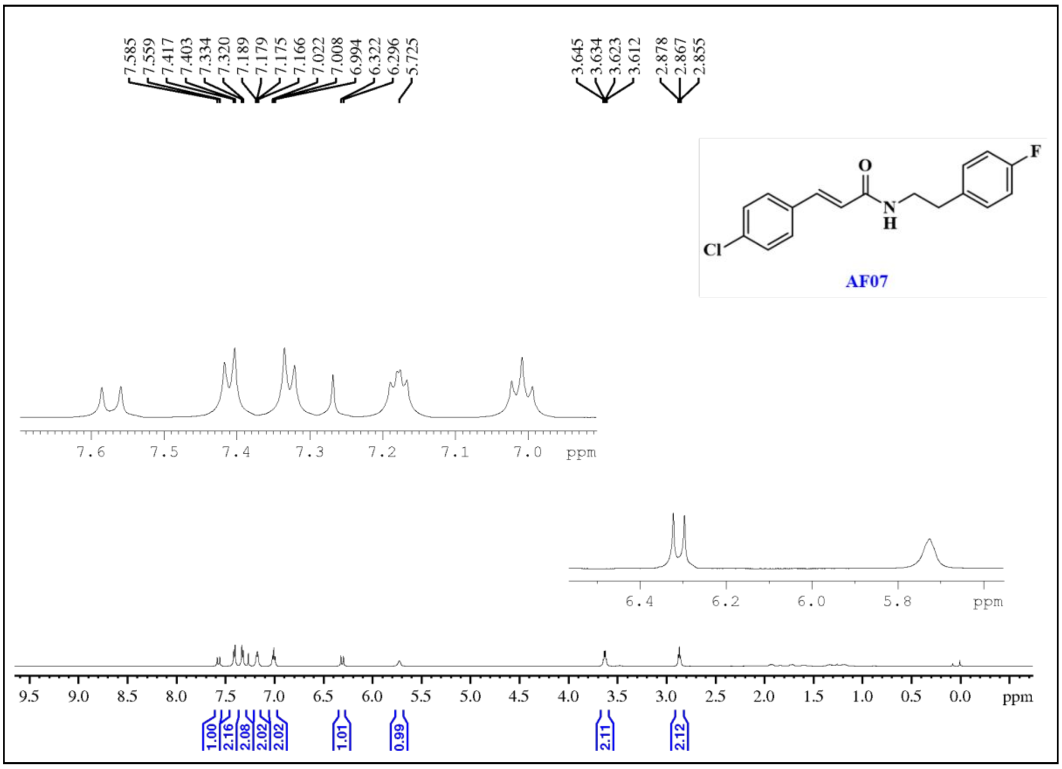

Figure S37. ^{1}H NMR spectrum of (*E*)-3-(4-chlorophenyl)-*N*-(4-fluorophenethyl)acrylamide (600 MHz, $CDCl_3$) (**AF07**)

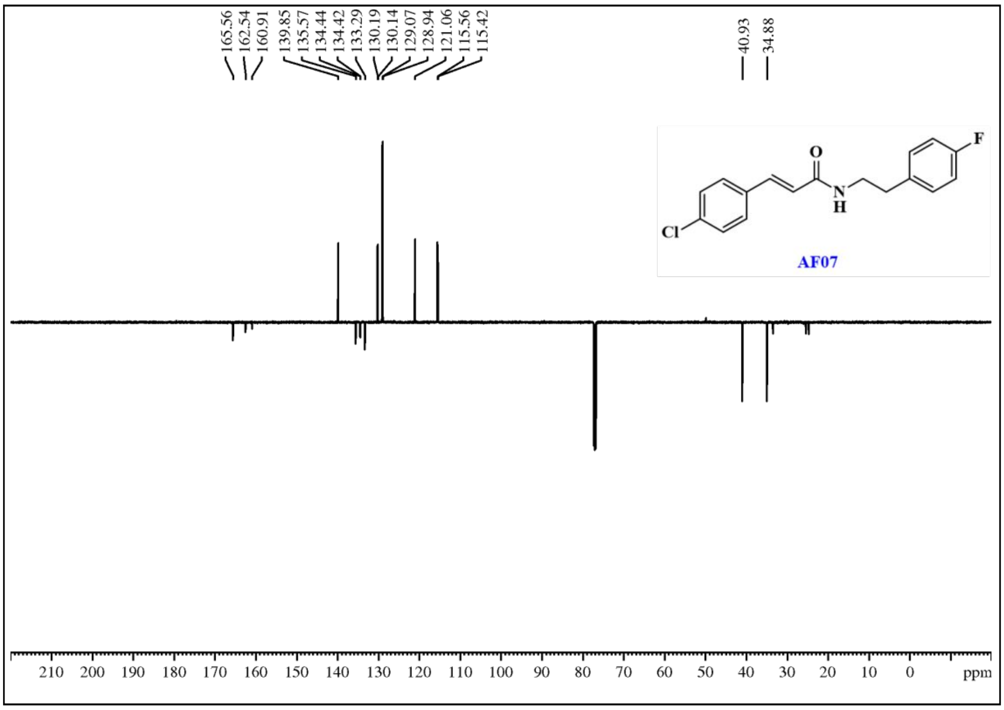

Figure S38. ^{13}C NMR spectrum of (*E*)-3-(4-chlorophenyl)-*N*-(4-fluorophenethyl)acrylamide (150 MHz, $CDCl_3$) (**AF07**)

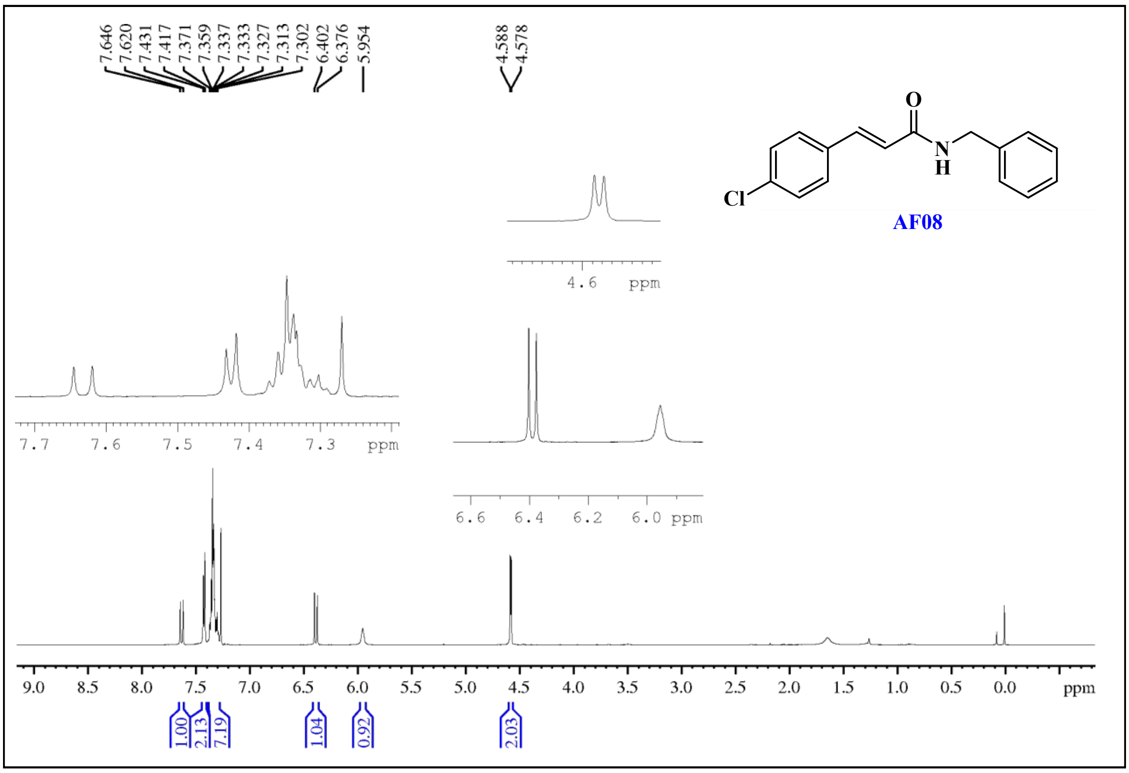

Figure S39. ^{1}H NMR spectrum of (*E*)-*N*-benzyl-3-(4-chlorophenyl)acrylamide (600 MHz, $CDCl_3$) (**AF08**)

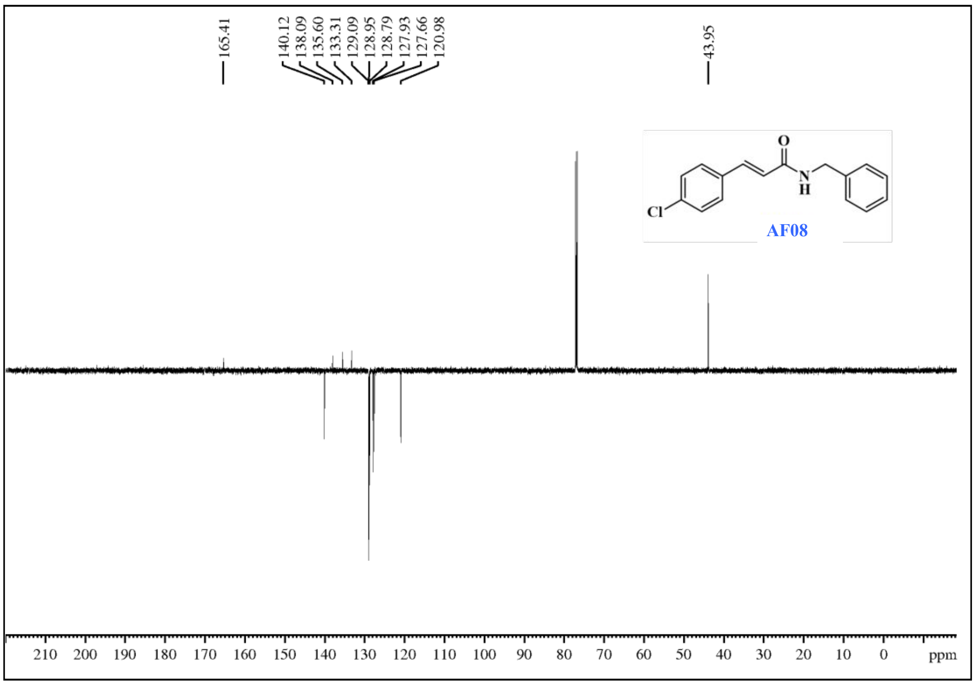

Figure S40. ^{13}C NMR spectrum of (*E*)-*N*-benzyl-3-(4-chlorophenyl)acrylamide (150 MHz, $CDCl_3$) (**AF08**)

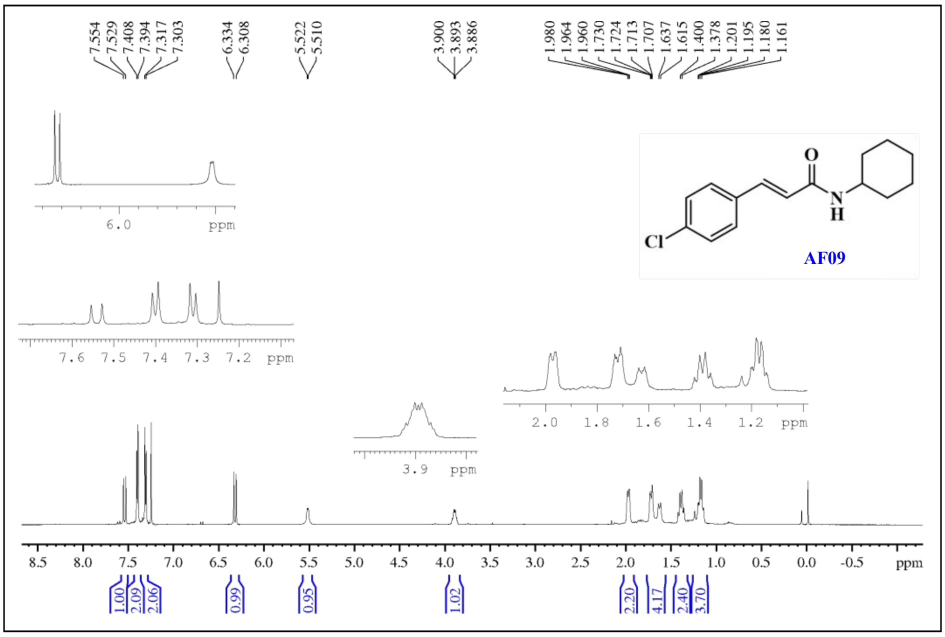

Figure S41. ^{1}H NMR spectrum of (*E*)-3-(4-chlorophenyl)-*N*-cyclohexylacrylamide (600 MHz, $CDCl_3$) (**AF09**)

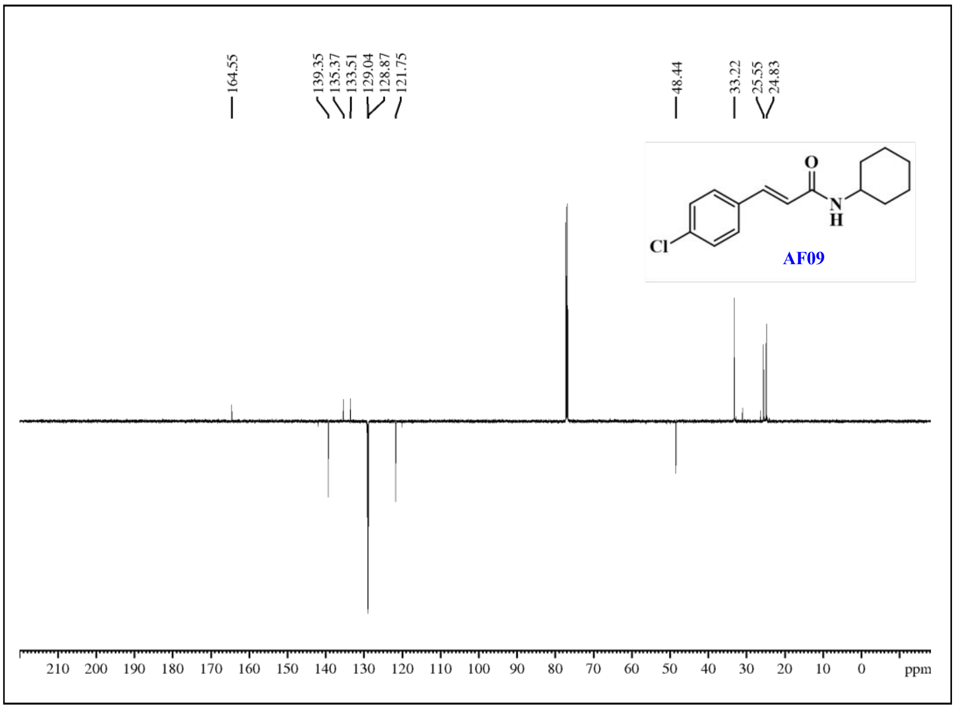

Figure S42. ^{13}C NMR spectrum of (*E*)-3-(4-chlorophenyl)-*N*-cyclohexylacrylamide (150 MHz, $CDCl_3$) (**AF09**)

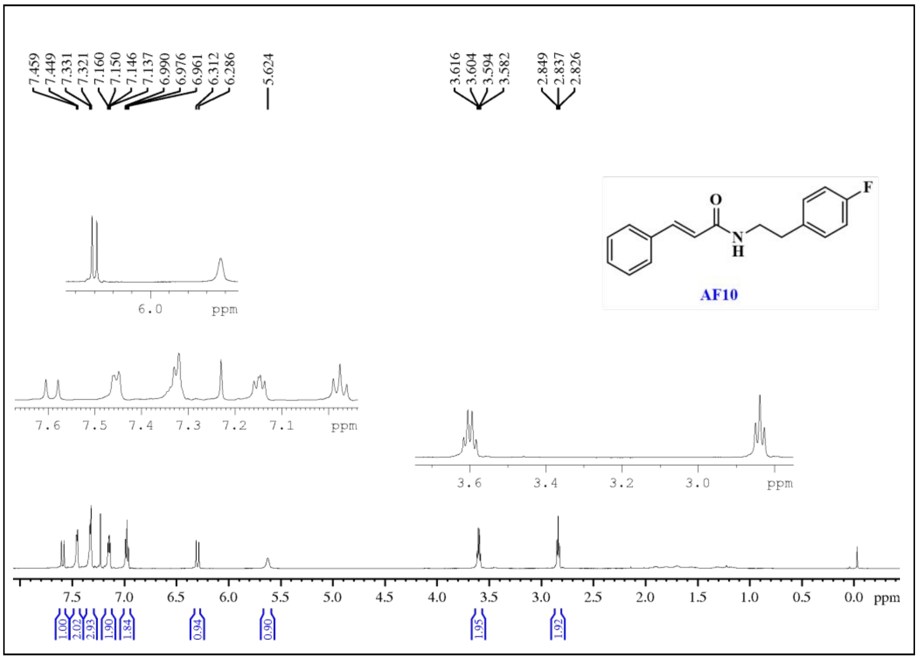

Figure S43. ^{1}H NMR spectrum of *N*-(4-fluorophenethyl)cinnamamide (600 MHz, $CDCl_3$) (**AF10**)

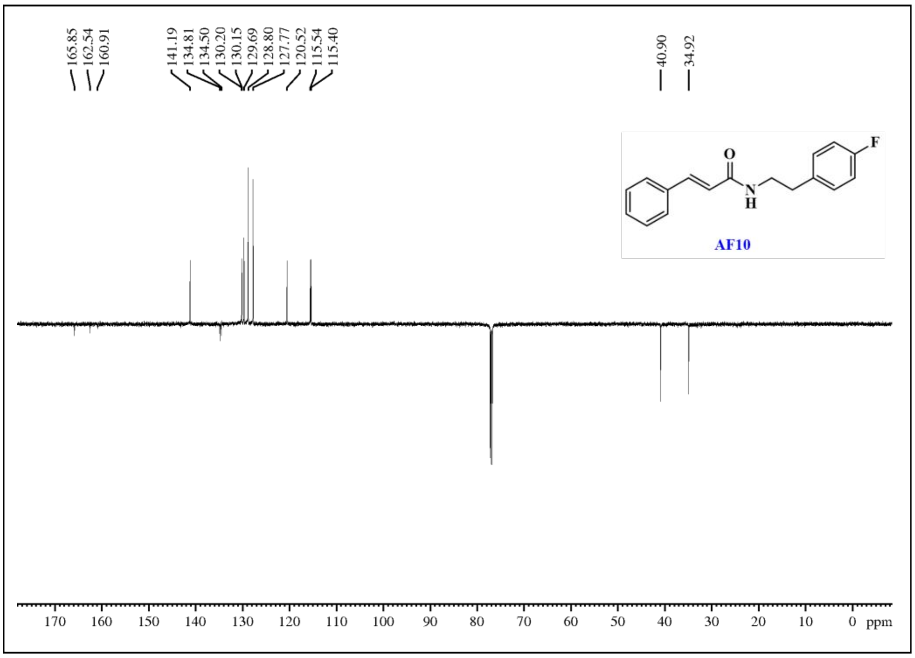

Figure S44. ^{13}C NMR spectrum of *N*-(4-fluorophenethyl)cinnamamide (150 MHz, $CDCl_3$) (**AF10**)

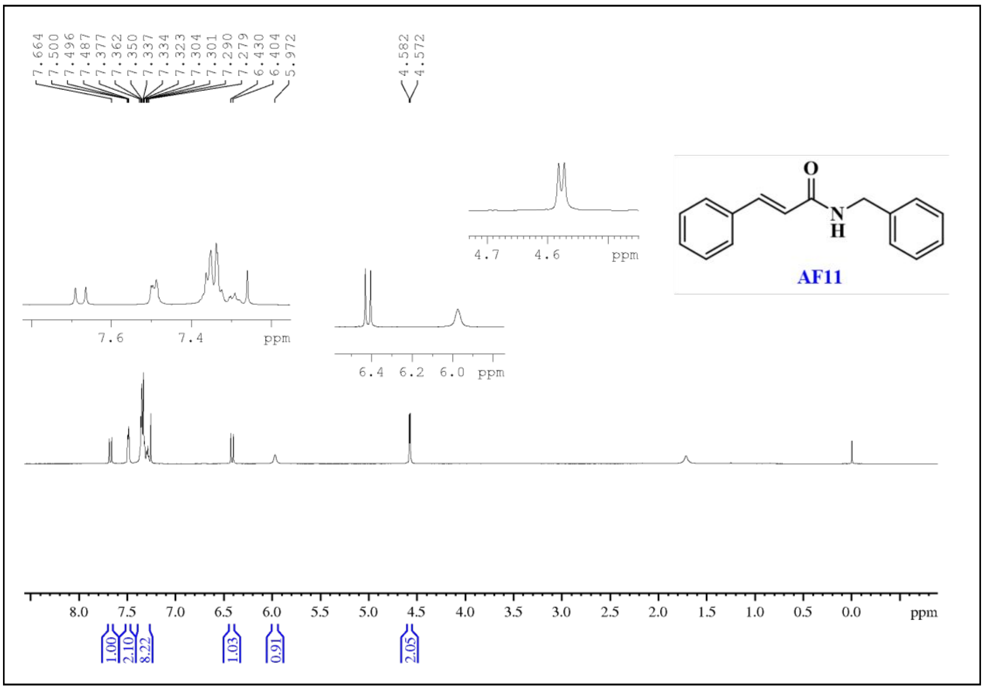

Figure S45. ^{1}H NMR spectrum of *N*-benzylcinnamamide (600 MHz, $CDCl_3$) (**AF11**)

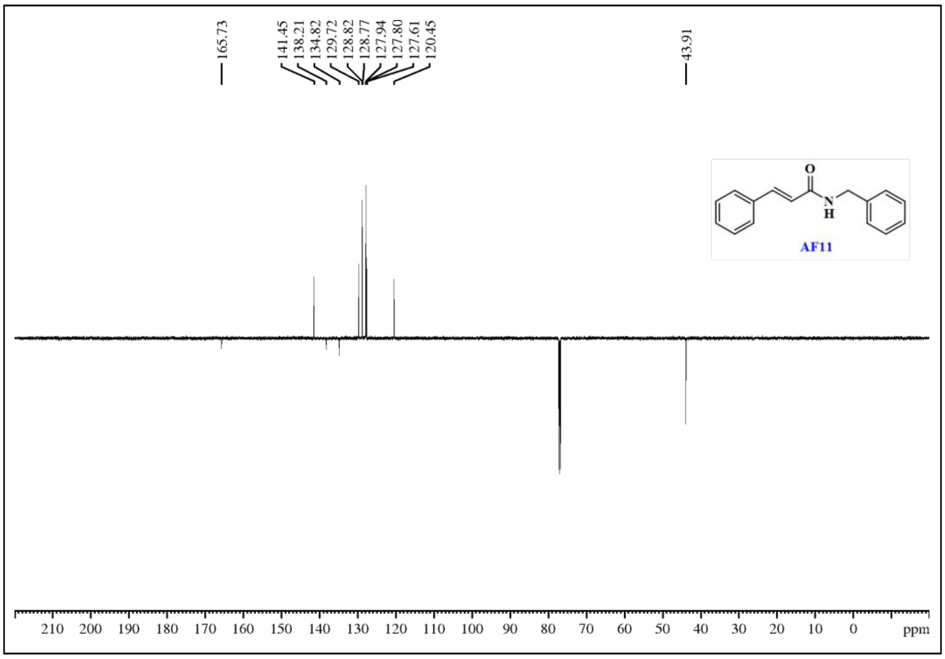

Figure S46. ^{13}C NMR spectrum of *N*-benzylcinnamamide (150 MHz, $CDCl_3$) (**AF11**)

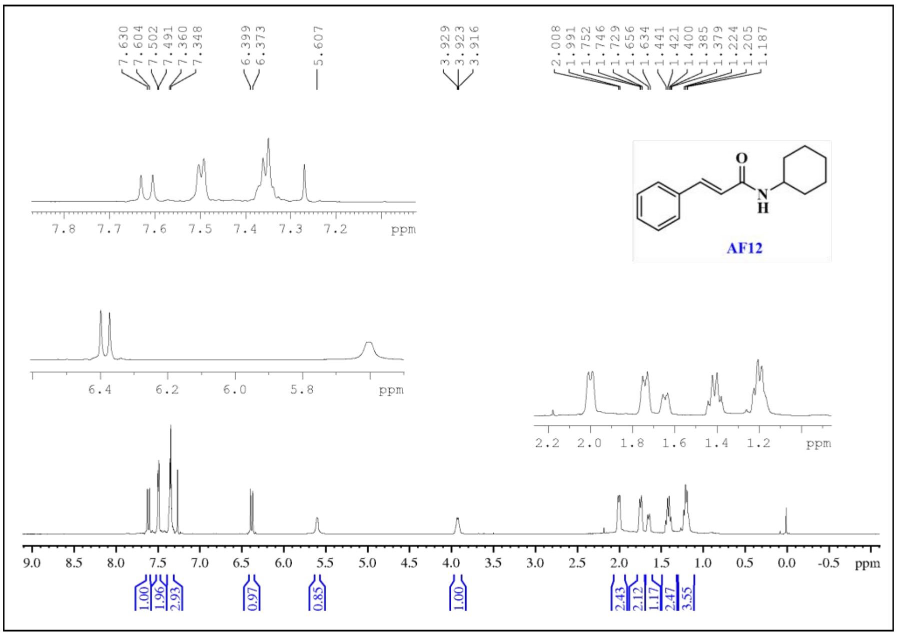

Figure S47. ^{1}H NMR spectrum of *N*-cyclohexylcinnamamide (600 MHz, $CDCl_3$) (**AF12**)

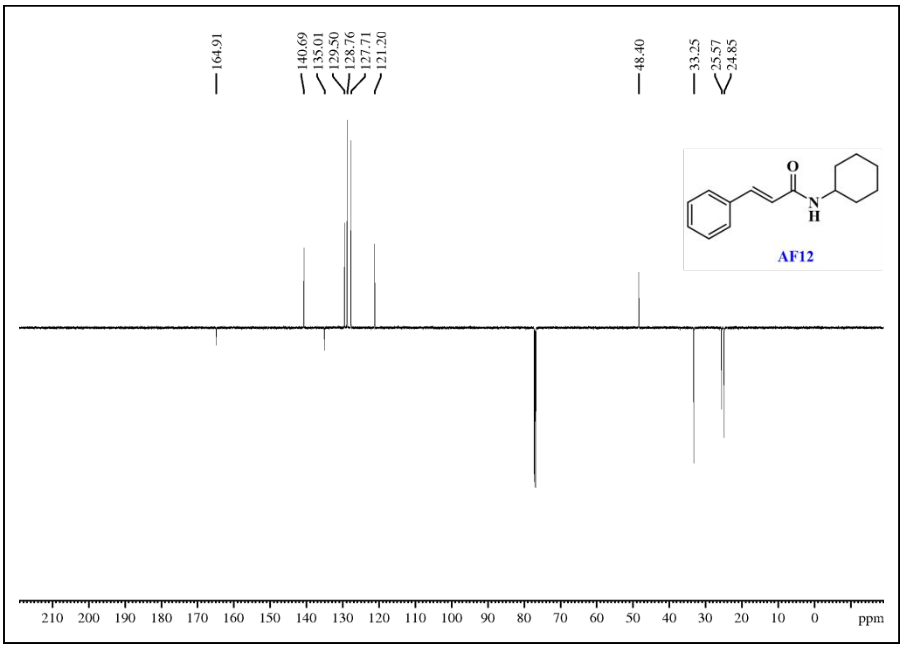

Figure S48. ^{13}C NMR spectrum of *N*-cyclohexylcinnamamide (150 MHz, $CDCl_3$) (**AF12**)

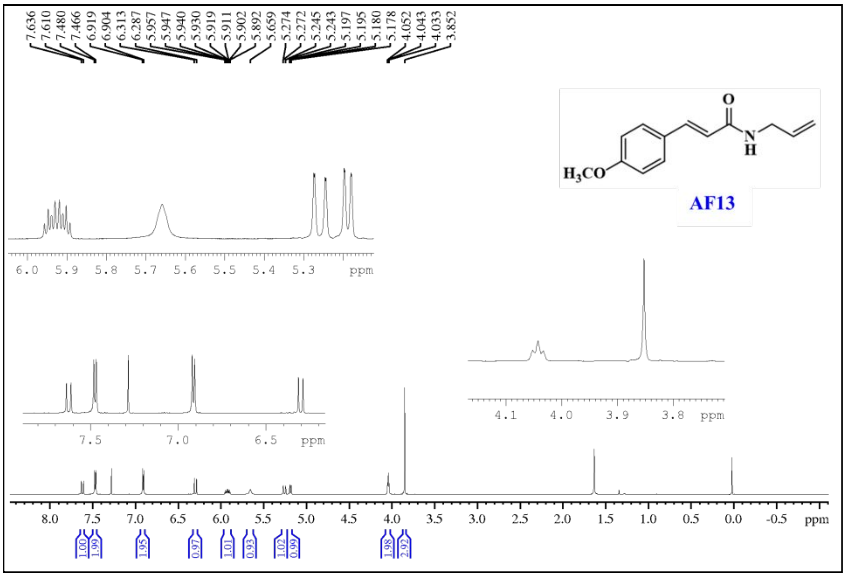

Figure S49. ^{1}H NMR spectrum of (*E*)-*N*-allyl-3-(4-methoxyphenyl)acrylamide (600 MHz, $CDCl_3$) (**AF13**)

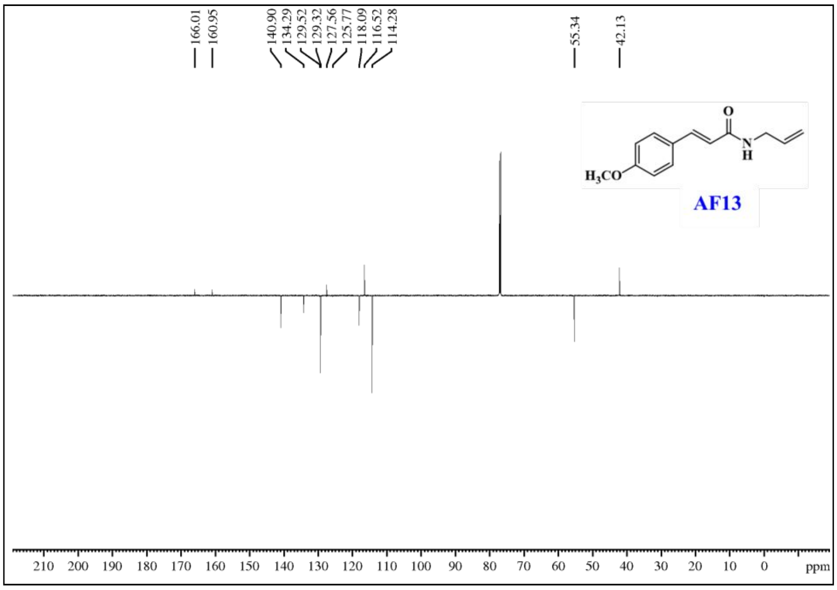

Figure S50. ^{13}C NMR spectrum of (*E*)-*N*-allyl-3-(4-methoxyphenyl)acrylamide (150 MHz, $CDCl_3$) (**AF13**)

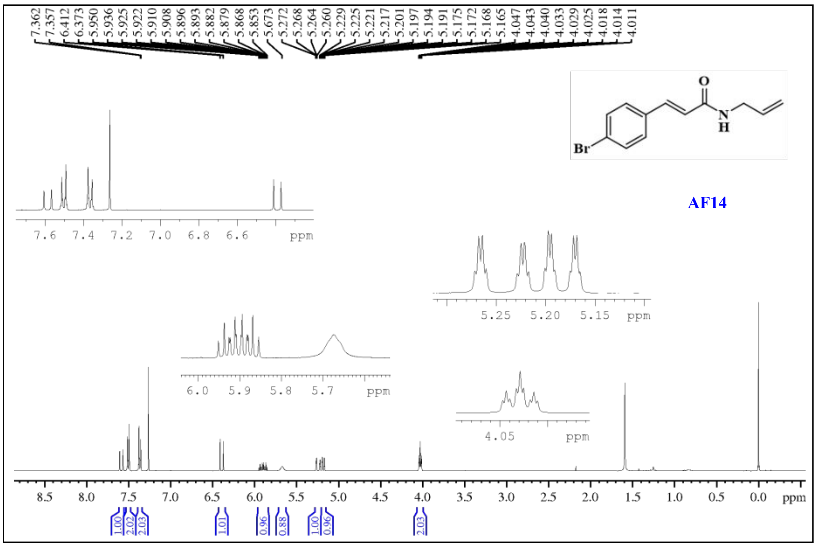

Figure S51. ^{1}H NMR spectrum of (*E*)-*N*-allyl-3-(4-bromophenyl)acrylamide (600 MHz, $CDCl_3$) (**AF14**)

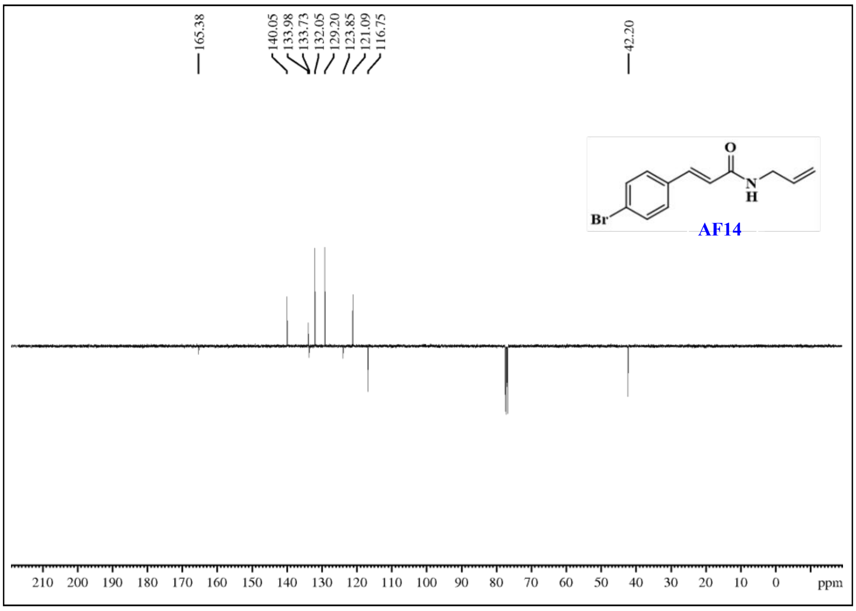

Figure S52. ^{13}C NMR spectrum of (*E*)-*N*-allyl-3-(4-bromophenyl)acrylamide (150 MHz, $CDCl_3$) (**AF14**)

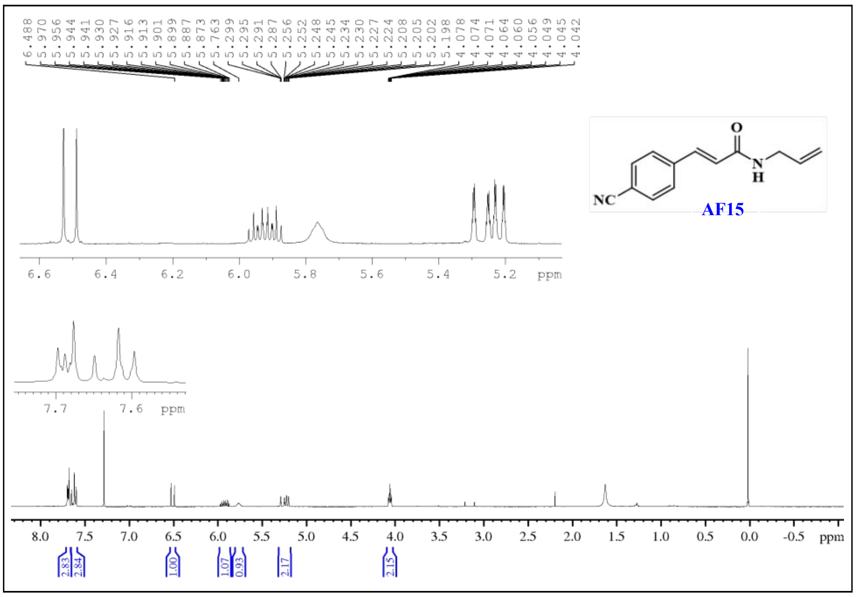

Figure S53. ^{1}H NMR spectrum of (*E*)-N-allyl-3-(4-cyanophenyl)acrylamide (600 MHz, $CDCl_3$) (**AF25**)

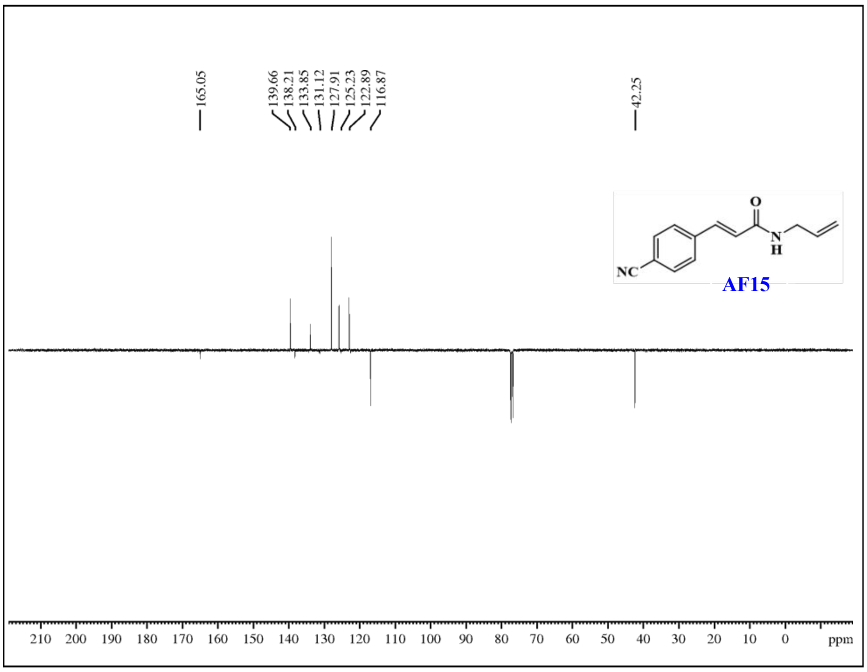

Figure S54. ^{13}C NMR spectrum (*E*)-*N*-allyl-3-(4-cyanophenyl)acrylamide (150 MHz, $CDCl_3$) (**AF15**)

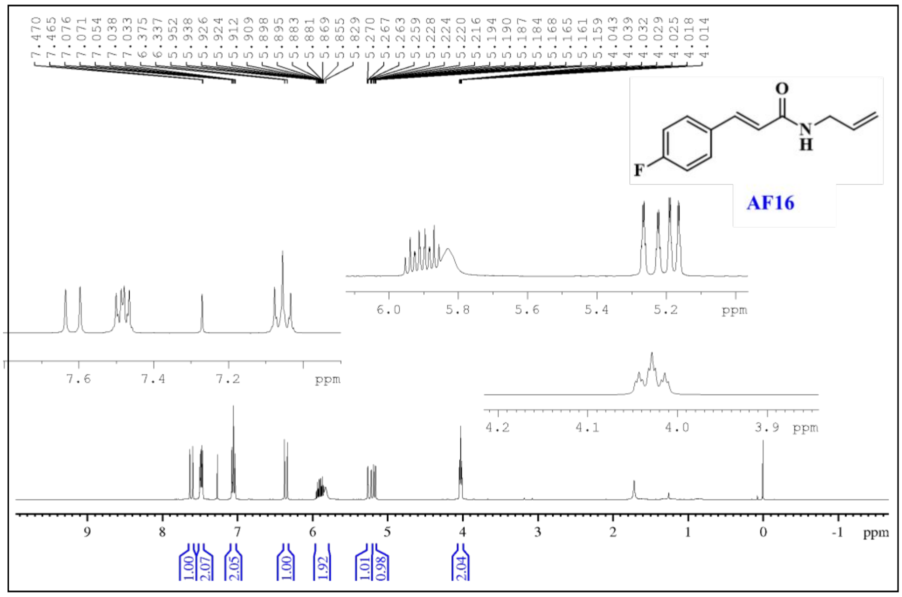

Figure S55. ^{1}H NMR spectrum of (*E*)-*N*-allyl-3-(4-fluorophenyl)acrylamide (600 MHz, $CDCl_3$) (**AF16**)

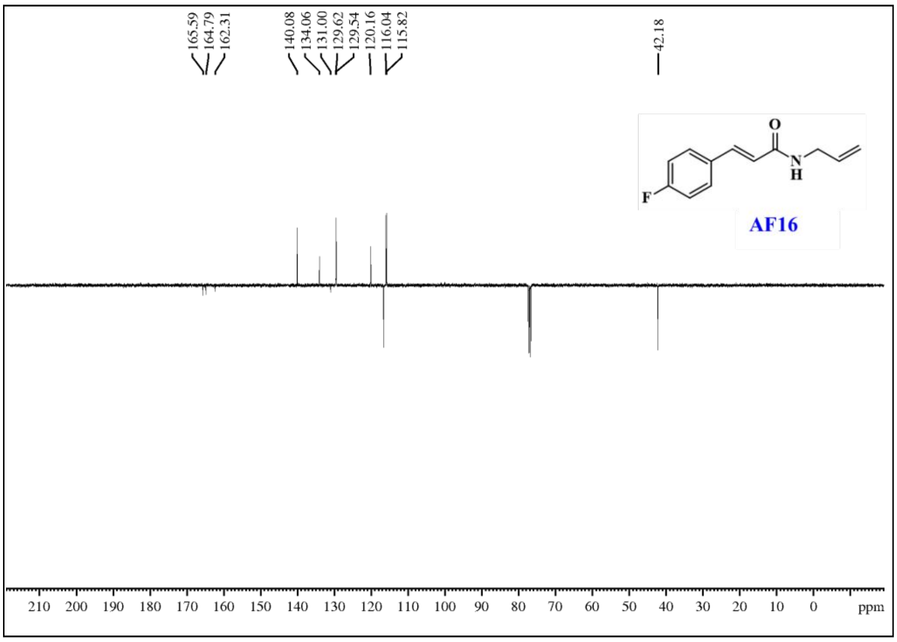

Figure S56. ^{13}C NMR spectrum (*E*)-*N*-allyl-3-(4-fluorophenyl)acrylamide (150 MHz, $CDCl_3$) (**AF16**)

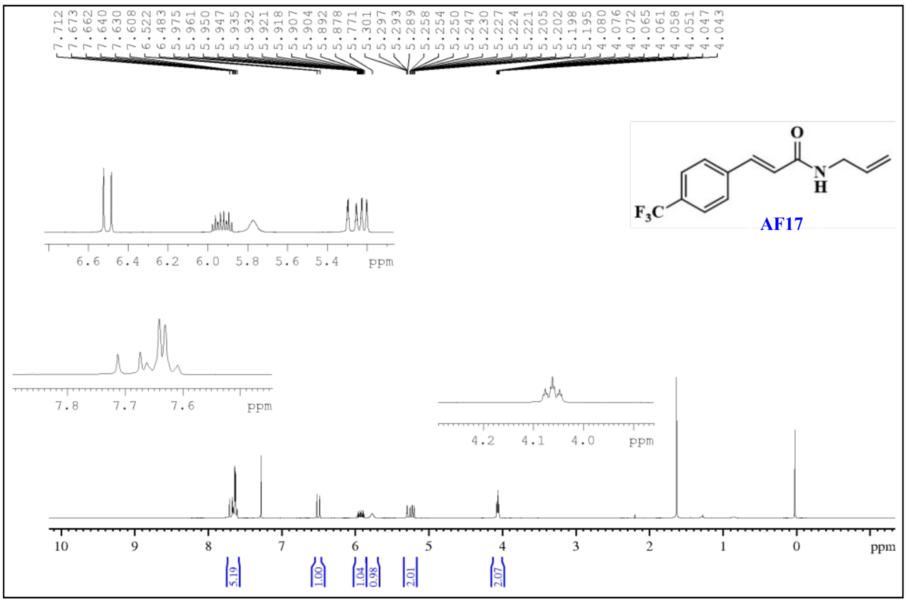

Figure S57 ^{1}H NMR spectrum of (*E*)-*N*-allyl-3-(4-(trifluoromethyl)phenyl)acrylamide (600 MHz, $CDCl_3$) (**AF17**)

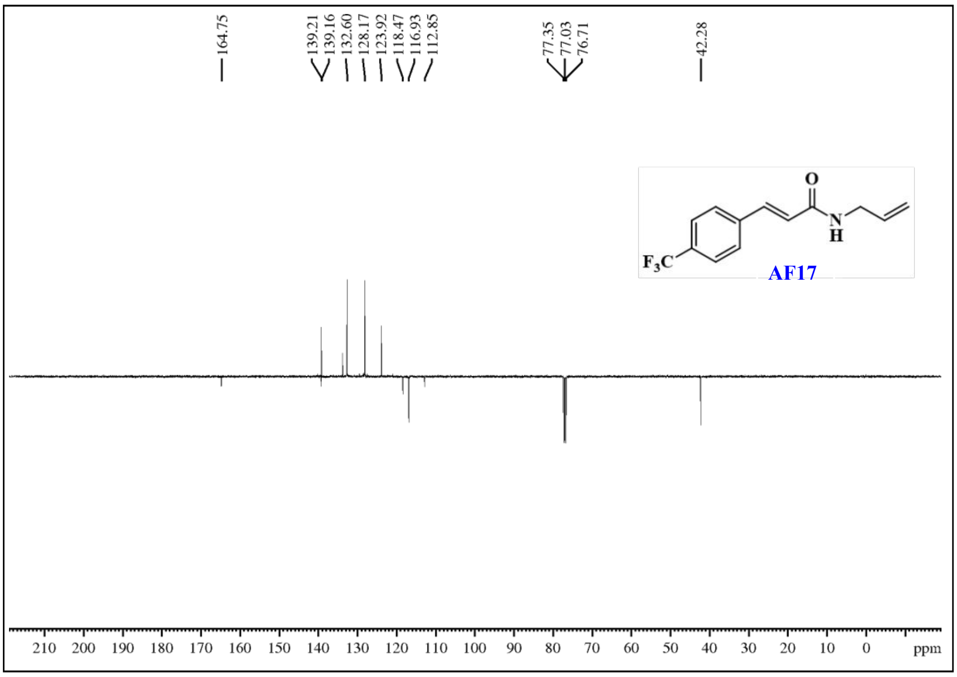

Figure S58. ^{13}C NMR spectrum (*E*)-*N*-allyl-3-(4-(trifluoromethyl)phenyl)acrylamide (150 MHz, $CDCl_3$) (**AF17**)

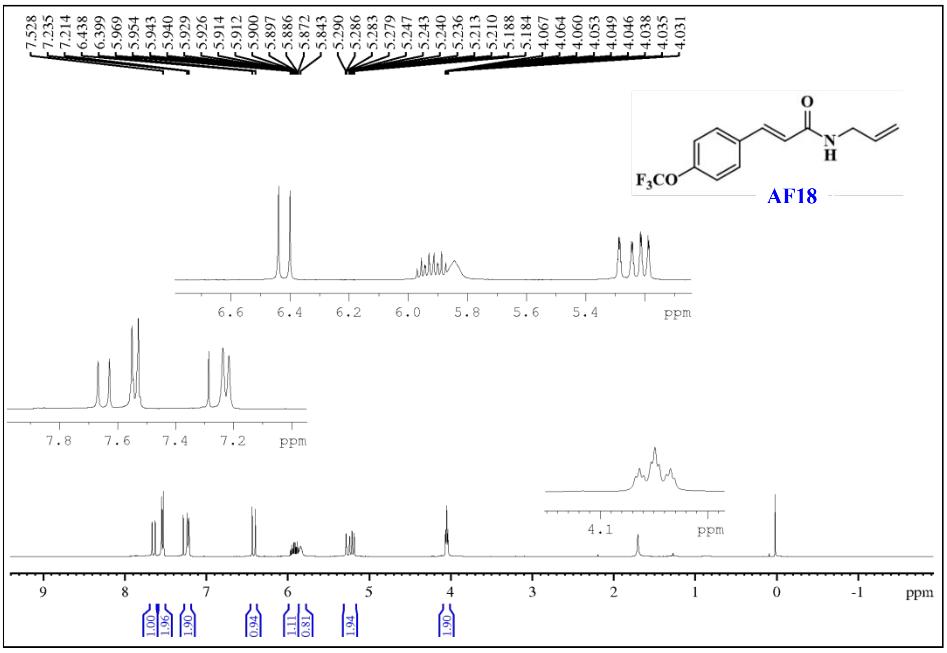

Figure S59. ^{1}H NMR spectrum of (*E*)-*N*-allyl-3-(4-(trifluoromethoxy)phenyl)acrylamide (600 MHz, $CDCl_3$) (**AF18**)

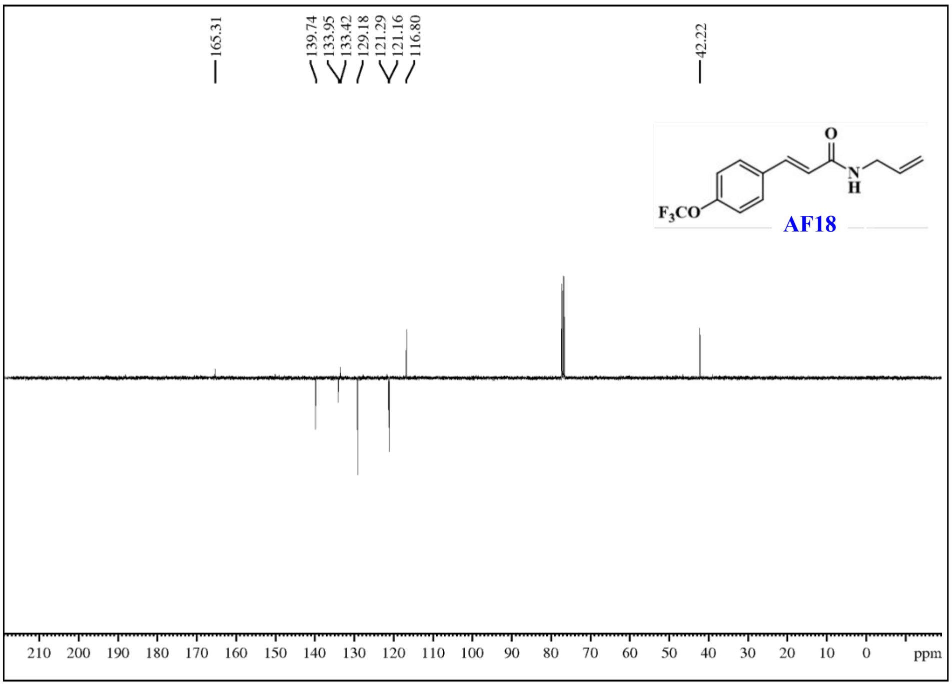

Figure S60. ^{13}C NMR spectrum of (*E*)-*N*-allyl-3-(4-(trifluoromethoxy)phenyl)acrylamide (150 MHz, $CDCl_3$) (**AF18**)

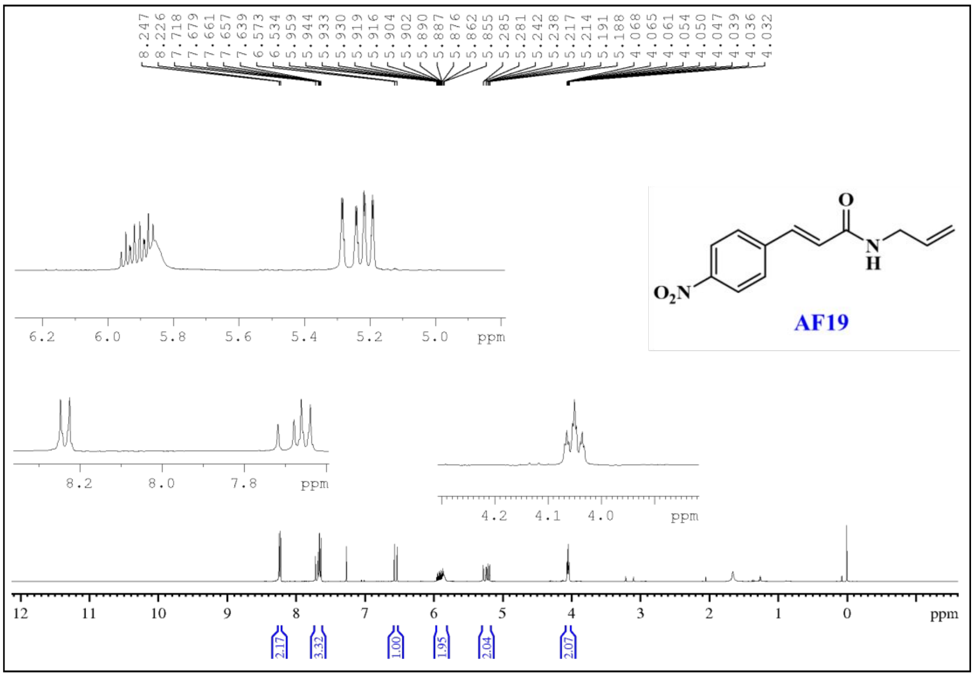

Figure S61. ^{1}H NMR spectrum of (*E*)-*N*-allyl-3-(4-nitrophenyl)acrylamide (600 MHz, $CDCl_3$) (**AF19**)

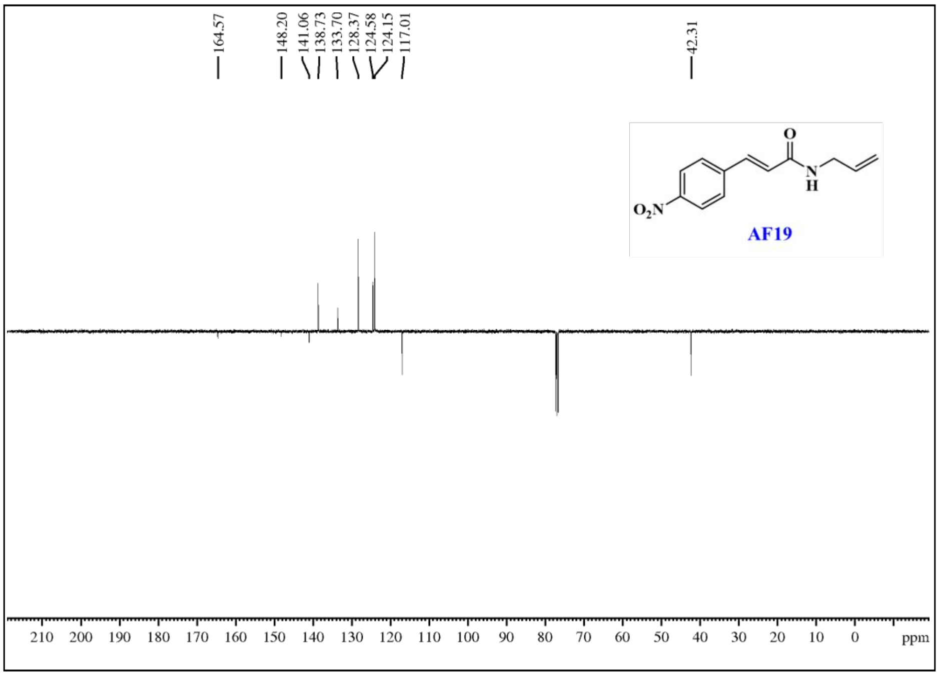

Figure S62. ^{13}C NMR spectrum (*E*)-*N*-allyl-3-(4-nitrophenyl)acrylamide (150 MHz, $CDCl_3$) (**AF19**)

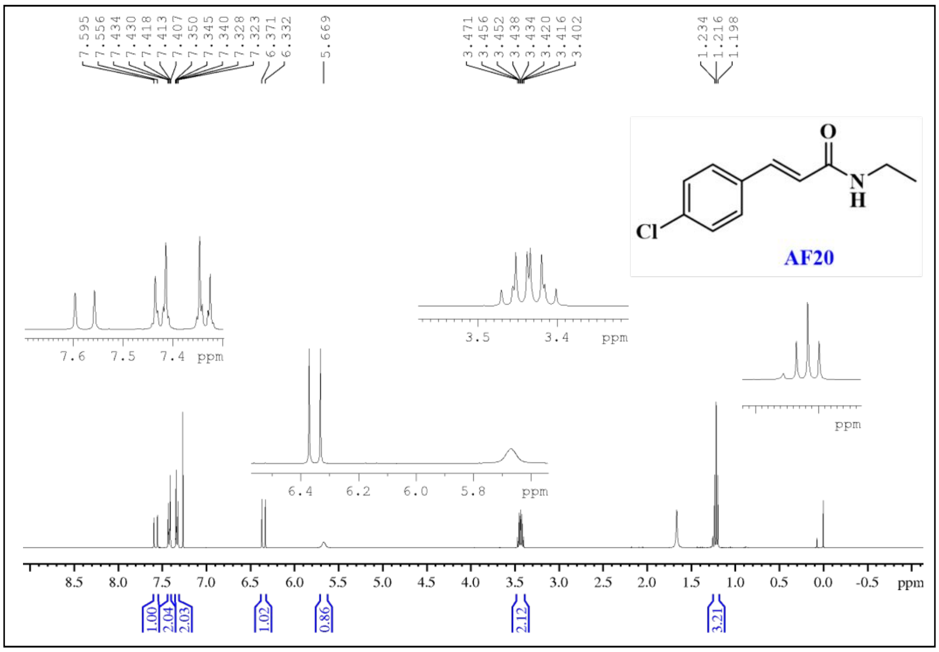

Figure S63. ^{1}H NMR spectrum of (*E*)-3-(4-chlorophenyl)-*N*-ethylacrylamide (400 MHz, $CDCl_3$) (**AF20**)

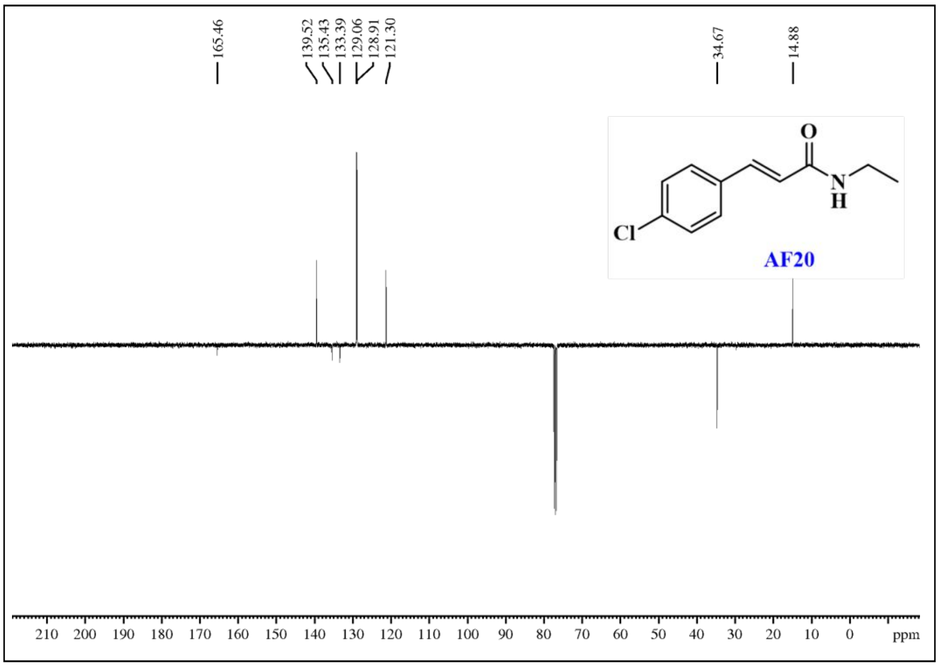

Figure S64. ^{13}C NMR spectrum of (*E*)-3-(4-chlorophenyl)-*N*-ethylacrylamide (100 MHz, $CDCl_3$) (**AF20**)

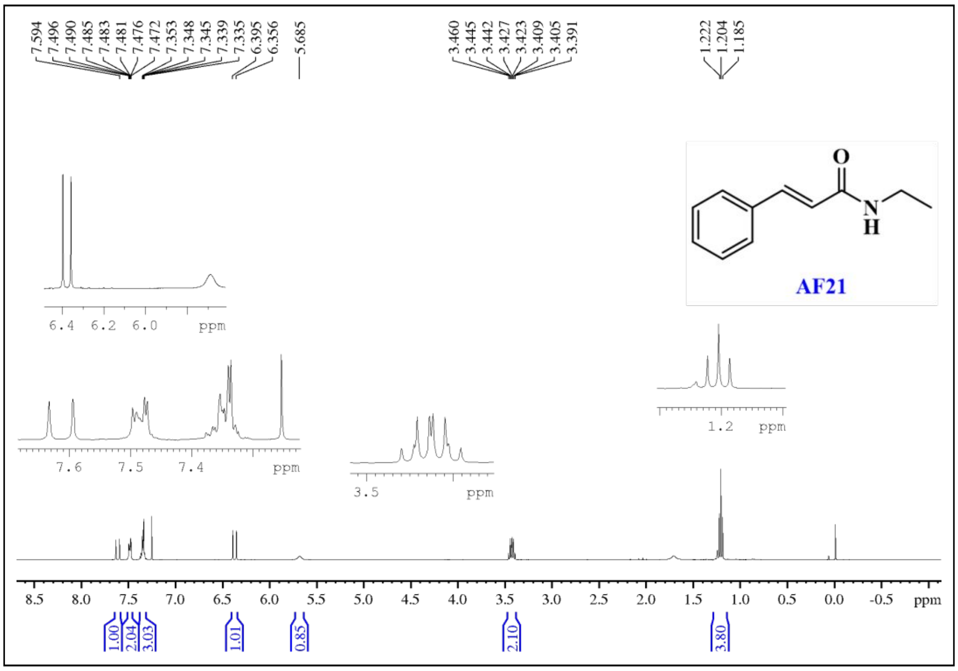

Figure S65. ^{1}H NMR spectrum of Ethylcinnamamide (400 MHz, $CDCl_3$) (**AF21**)

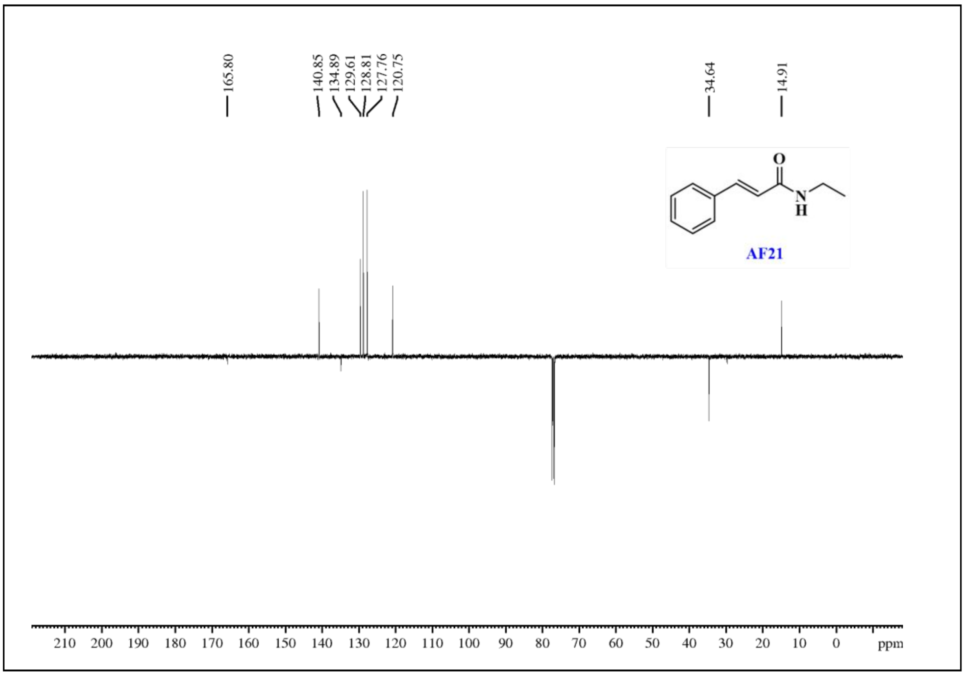

Figure S66. ^{13}C NMR spectrum of Ethylcinnamamide (100 MHz, $CDCl_3$) (**AF21**)

6. FTIR spectra of cinnamamides

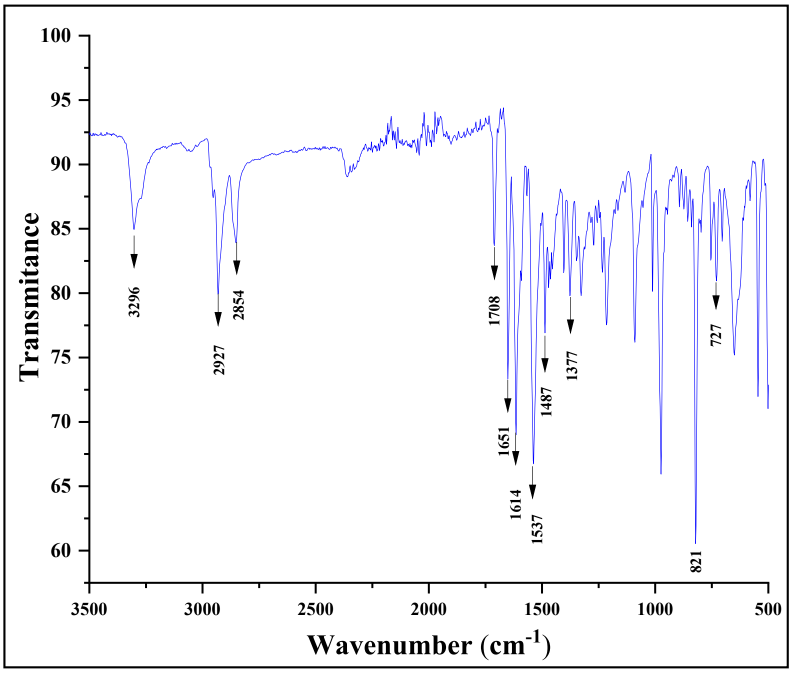

Figure S67. FT-IR of the compound (*E*)-3-(4-chlorophenyl)-*N*-hexylacrylamide **(AF01)**

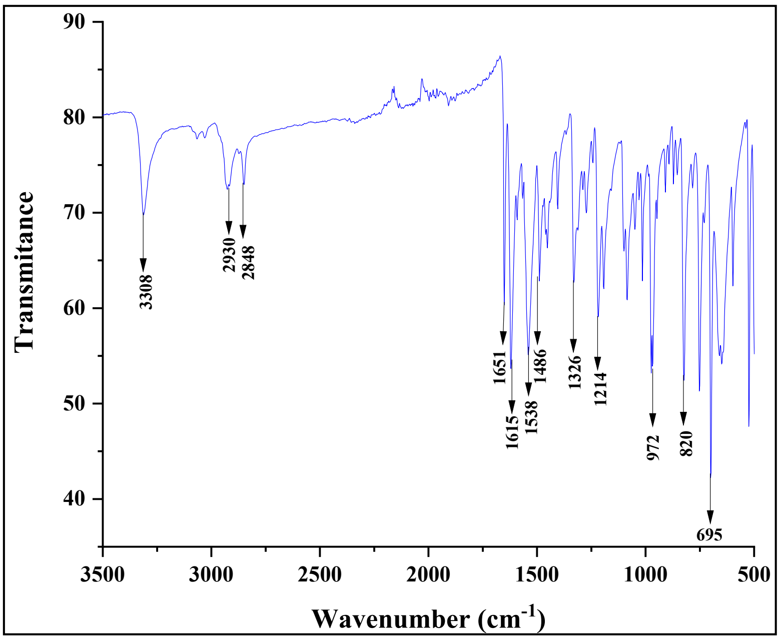

Figure S68. FT-IR of the compound (*E*)-3-(4-chlorophenyl)-*N*-phenethylacrylamide **(AF02)**

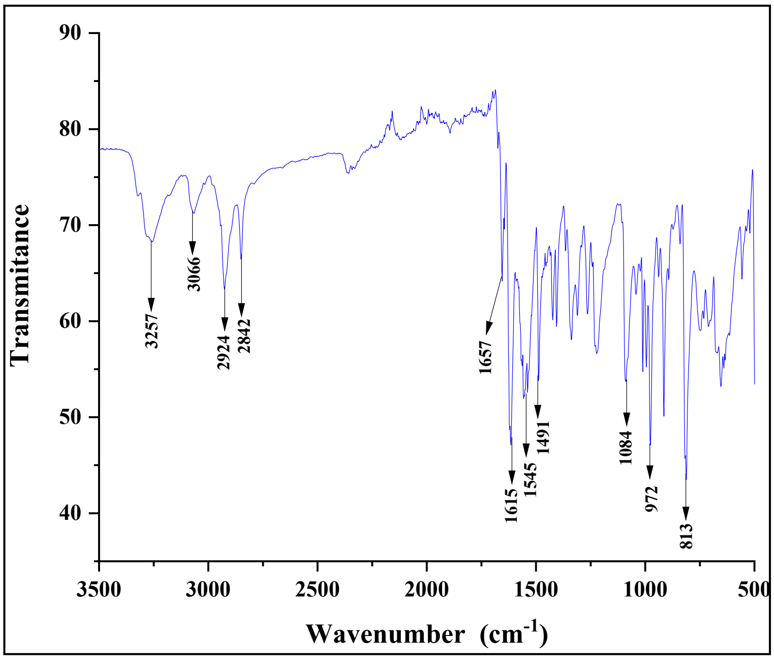

Figure S69. FT-IR of the compound (*E*)-*N*-allyl-3-(4-chlorophenyl)acrylamide (**AF03**)

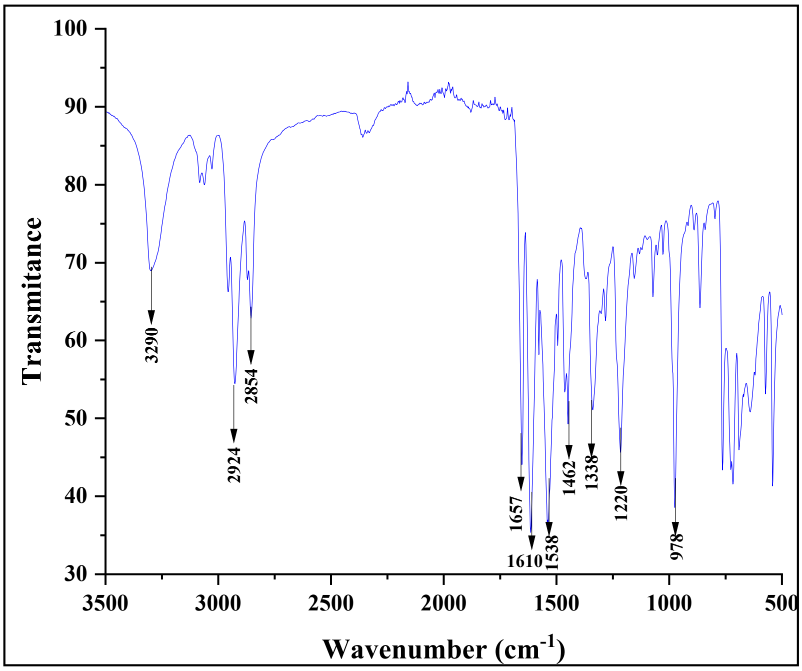

Figure S70. FT-IR of compoud *N*-hexylcinnamamide **(AF04)**

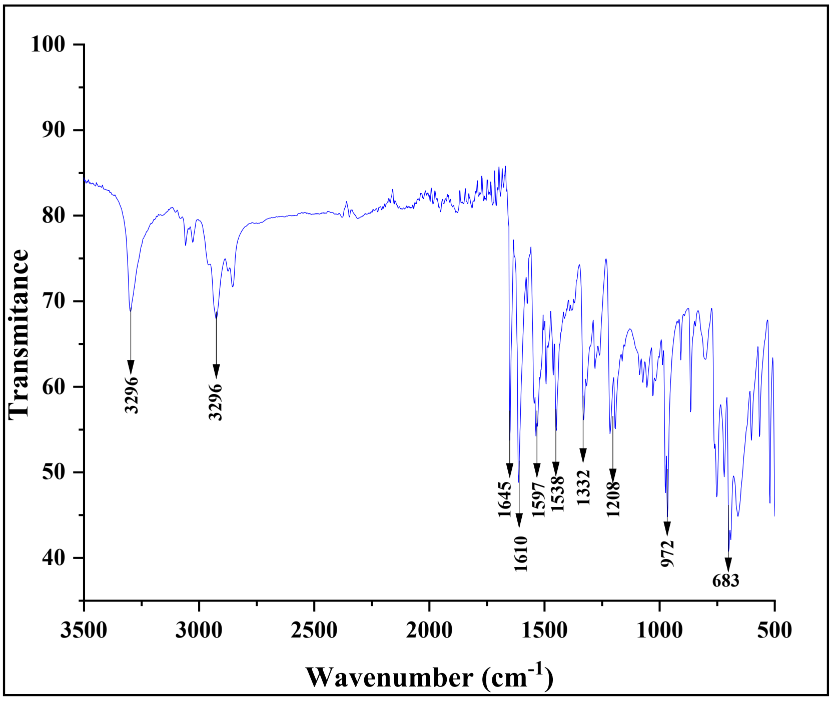

Figure S71. FT-IR of compoud (*E*)-*N*-phenethylcinnamamide **(AF05)**

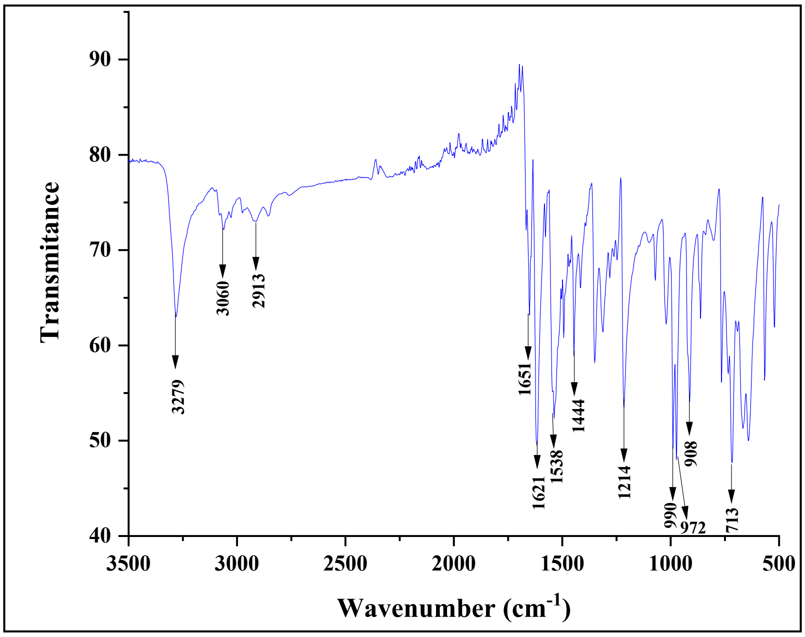

Figure S72. FT-IR of compoud *N*-allylcinnamamide (**AF06**)

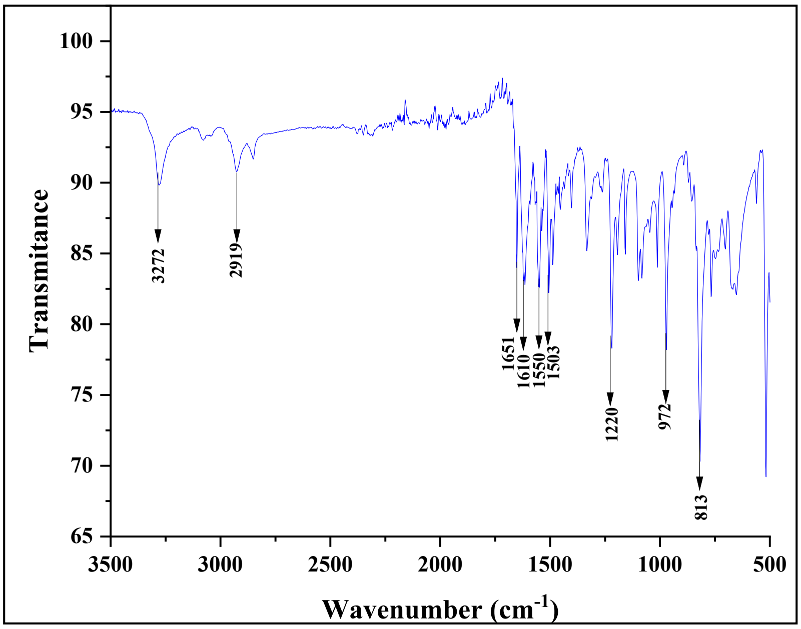

Figure S73. FT-IR of compoud (*E*)-3-(4-chlorophenyl)-*N*-(4-fluorophenethyl)acrylamide **(AF07)**

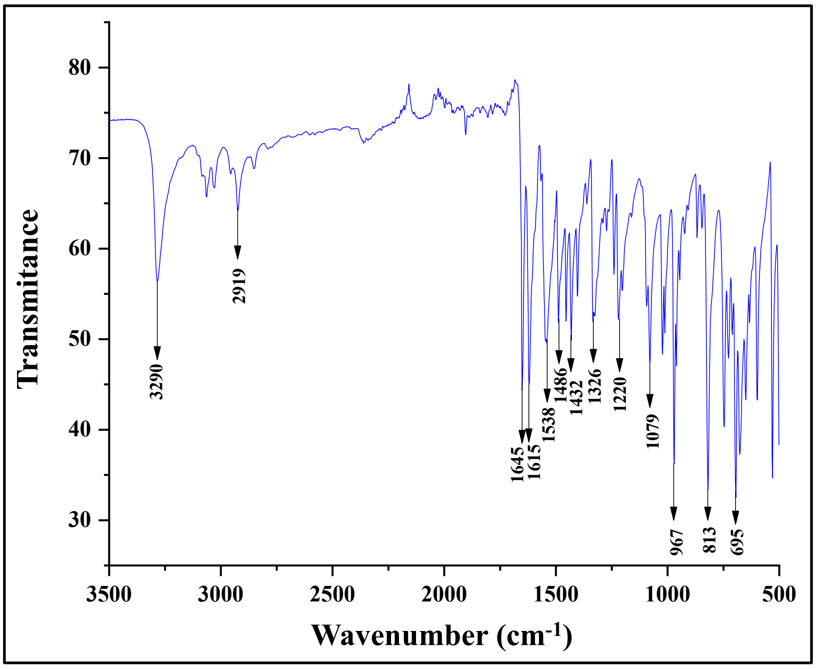

Figure S74. FT-IR of compound (*E*)-*N*-benzyl-3-(4-chlorophenyl)acrylamide (**AF08**)

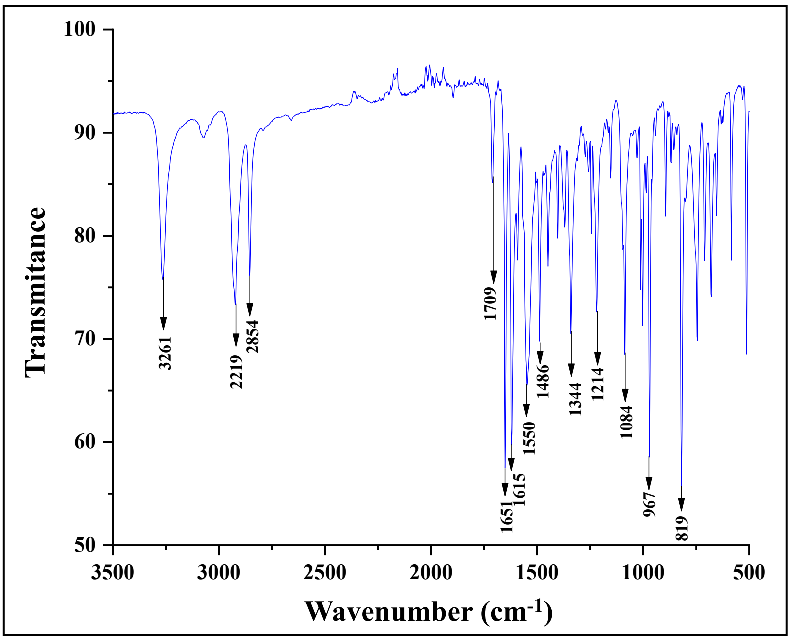

Figure S75. FT-IR of compound (*E*)-3-(4-chlorophenyl)-*N*-cyclohexylacrylamide (**AF09**)

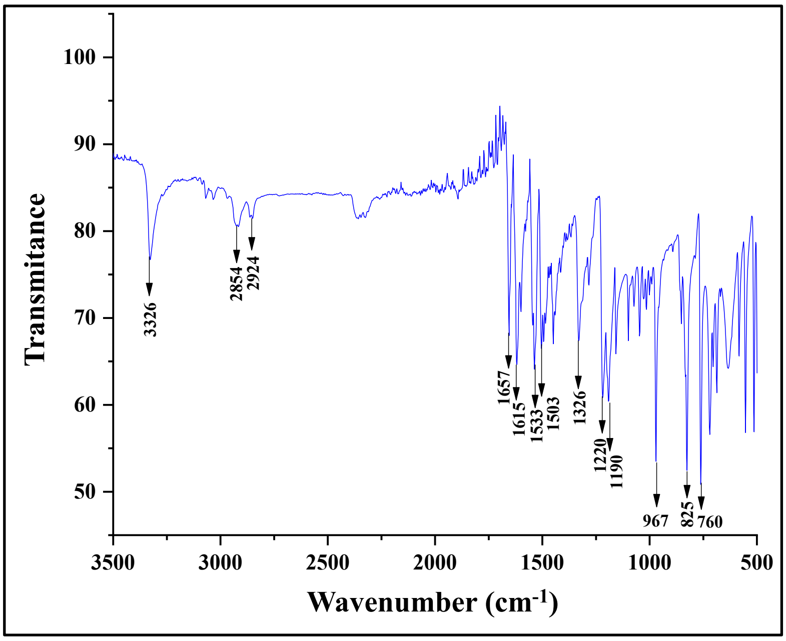

Figure S76. FT-IR of compoud *N*-(4-fluorophenethyl)cinnamamide **(AF10)**

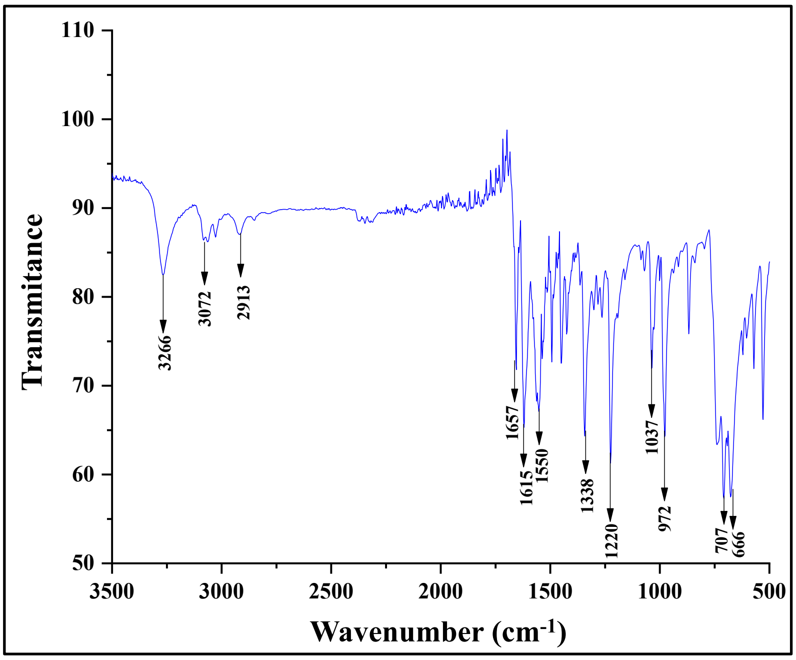

Figure S77. FT-IR of compoud *N*-Benzylcinnamamide **(AF11)**

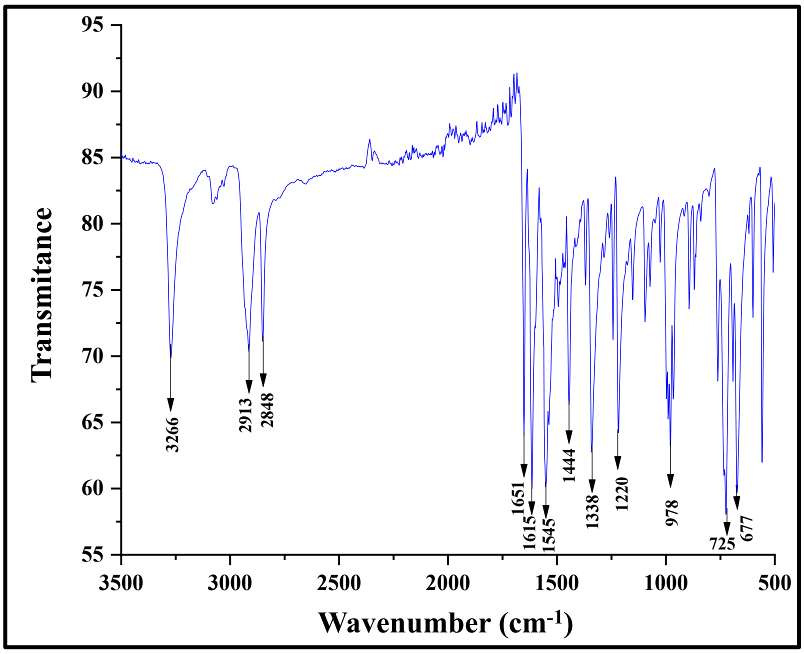

Figure S78. FT-IR of compoud *N*-cyclohexylcinnamamide (**AF12**)

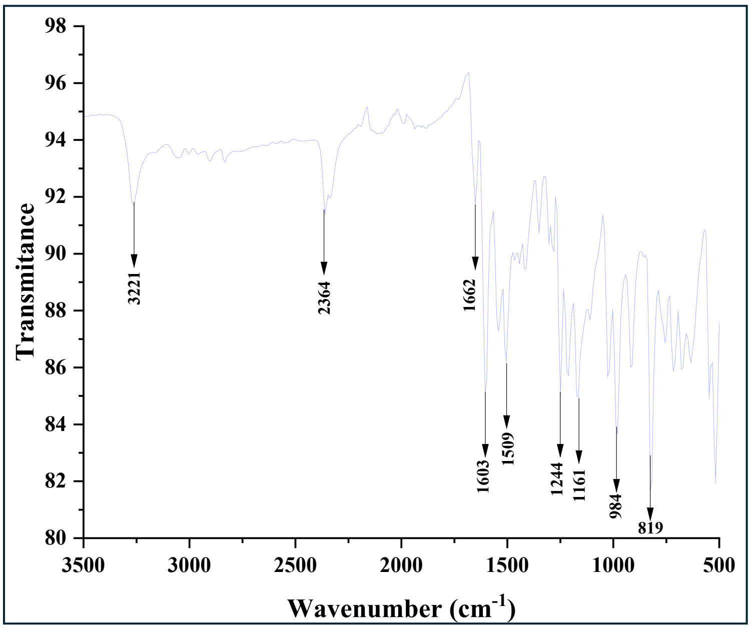

Figure S79. FT-IR of compoud (*E*)-*N*-allyl-3-(4-methoxyphenyl)acrylamide **(AF13)**

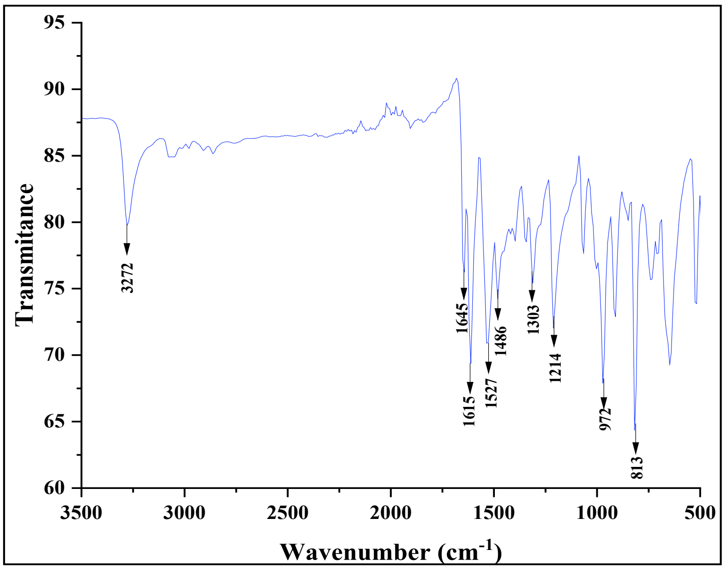

Figure S80. FT-IR of compoud (*E*)-*N*-allyl-3-(4-bromophenyl)acrylamide **(AF14)**

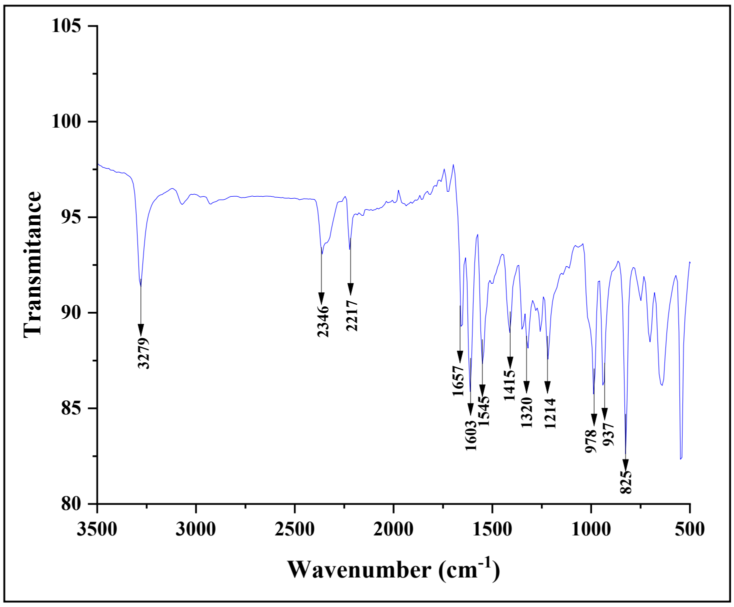

Figure S81. FT-IR of compound (*E*)-*N*-allyl-3-(4-cyanophenyl)acrylamide **(AF15)**

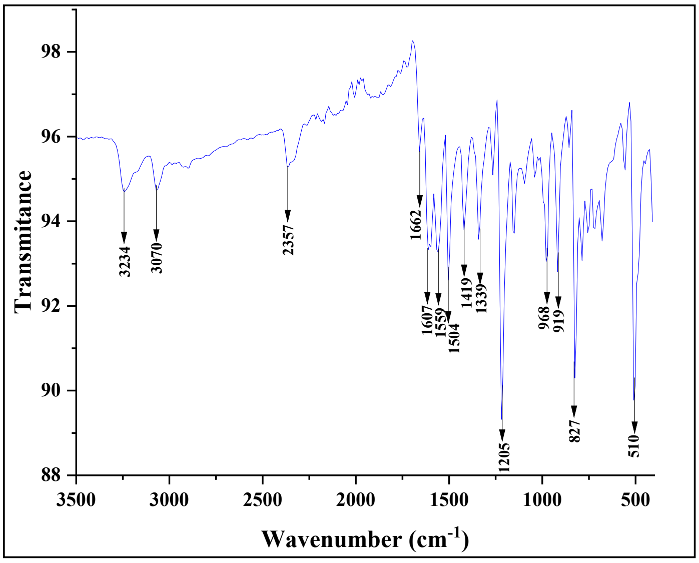

Figure S82. FT-IR of compound (*E*)-*N*-allyl-3-(4-fluorophenyl)acrylamide **(AF16)**

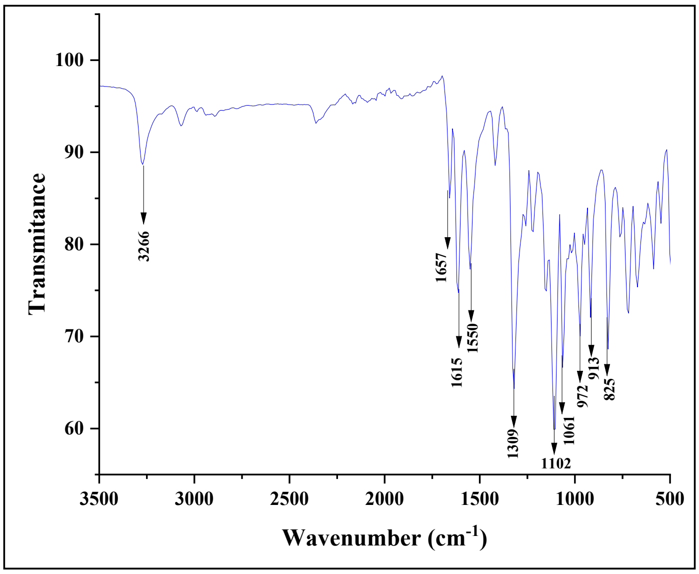

Figure S83. FT-IR of compound (*E*)-*N*-allyl-3-(4-(trifluoromethyl)phenyl)acrylamide **(AF17)**

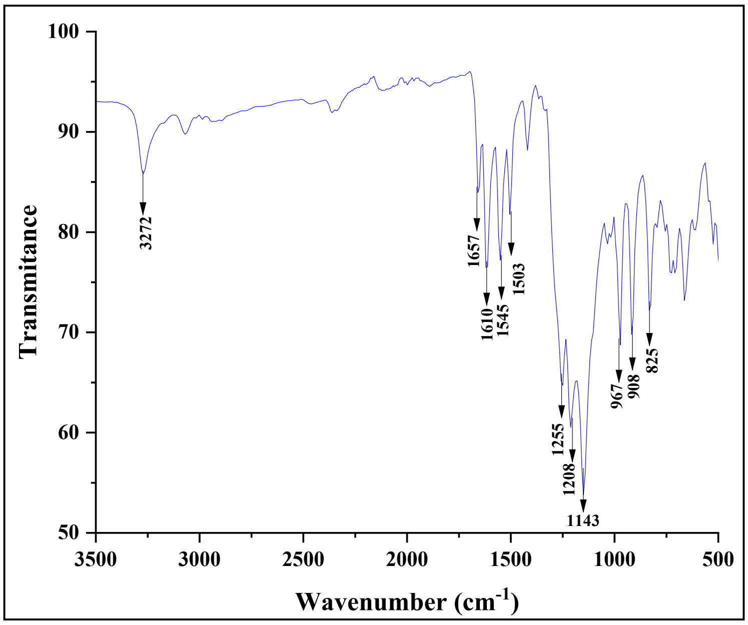

Figure S84. FT-IR of compound (*E*)-*N*-allyl-3-(4-(trifluoromethoxy)phenyl)acrylamide (**AF18**)

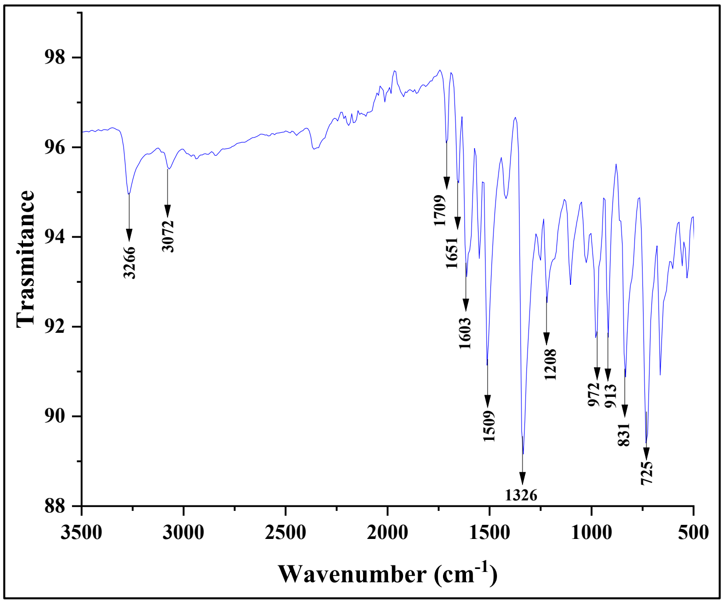

Figure S85. FT-IR of compoud (*E*)-*N*-allyl-3-(4-nitrophenyl)acrylamide **(AF19)**

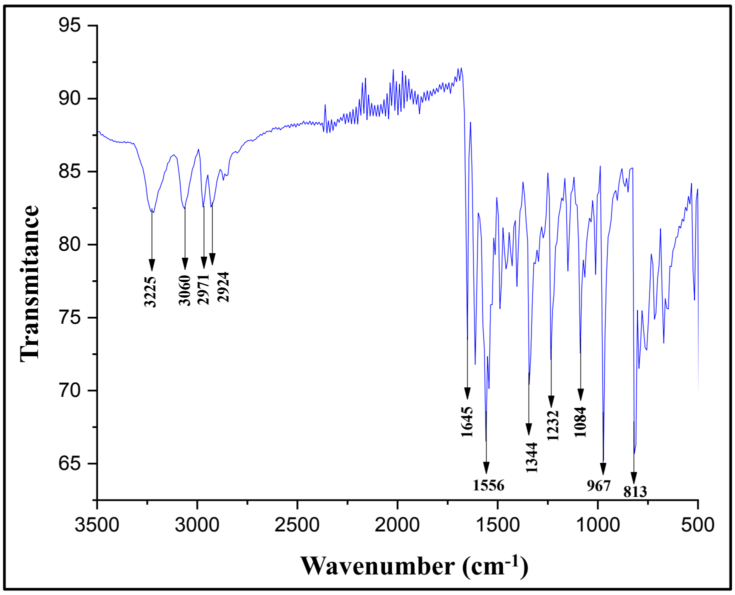

Figure S86. FT-IR of compoud (*E*)-3-(4-chlorophenyl)-*N*-ethylacrylamide (**AF20**)

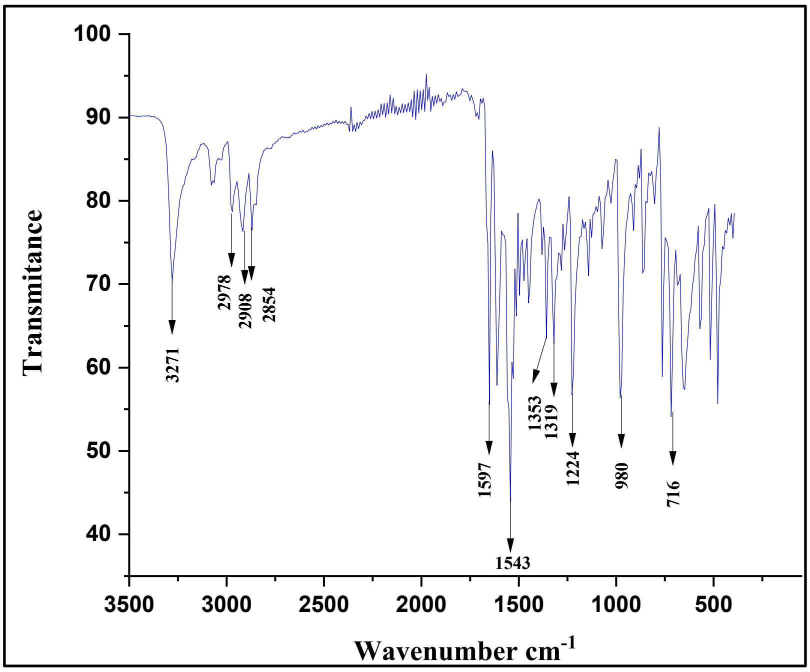

Figure S87. FT-IR of compoud Ethylcinnamamide (**AF21**)

7. HRMS and HPLC of cinnamamides

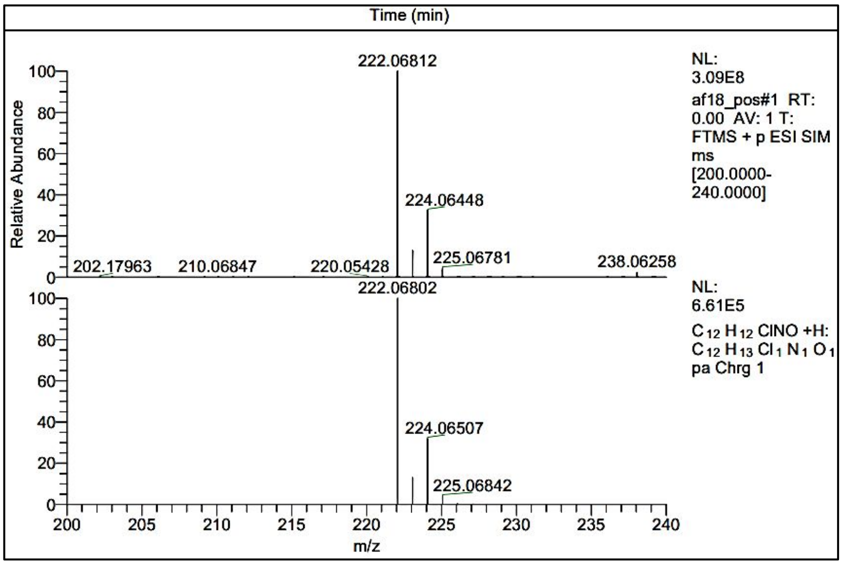

Figure S88. HRMS (ESI) of compoud (*E*)-*N*-allyl-3-(4-chlorophenyl)acrylamide (**AF03**)

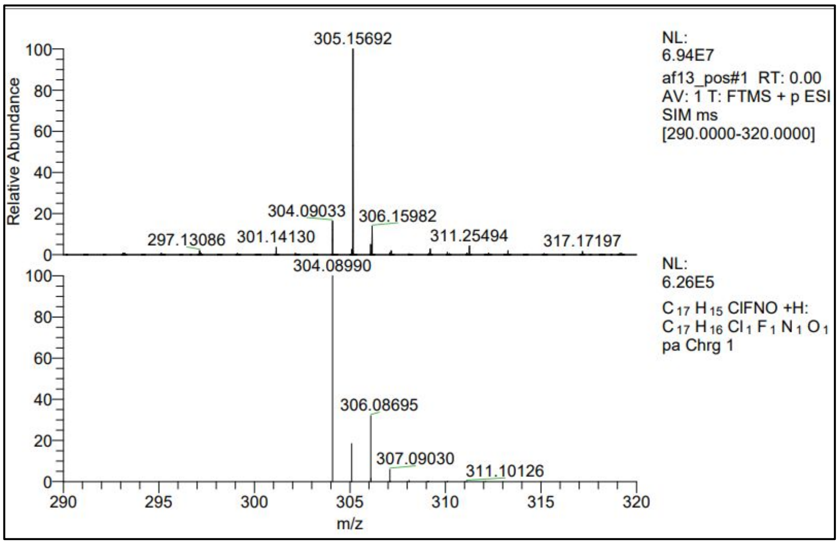

Figure S89. HMRS-ESI of compoud (*E*)-3-(4-chlorophenyl)-*N*-(4-fluorophenethyl)acrylamide (**AF07**)

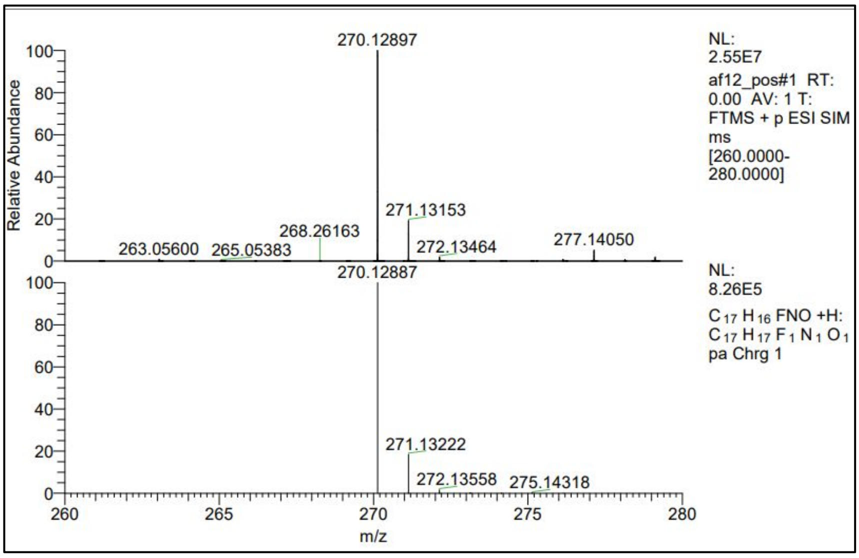

Figure S90. HMRS-ESI of compoud *N*-(4-fluorophenethyl)cinnamamide (**AF10**)

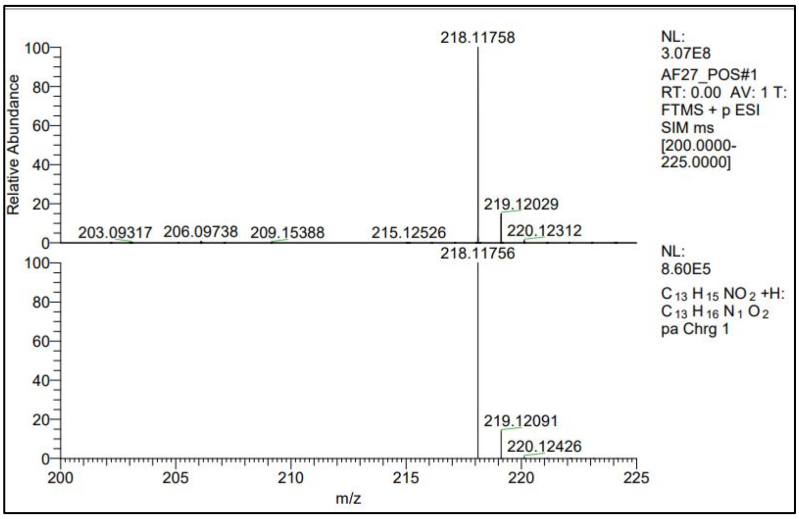

Figure S91. HRMS-ESI of compoud (*E*)-*N*-allyl-3-(4-methoxyphenyl)acrylamide **(AF13)**

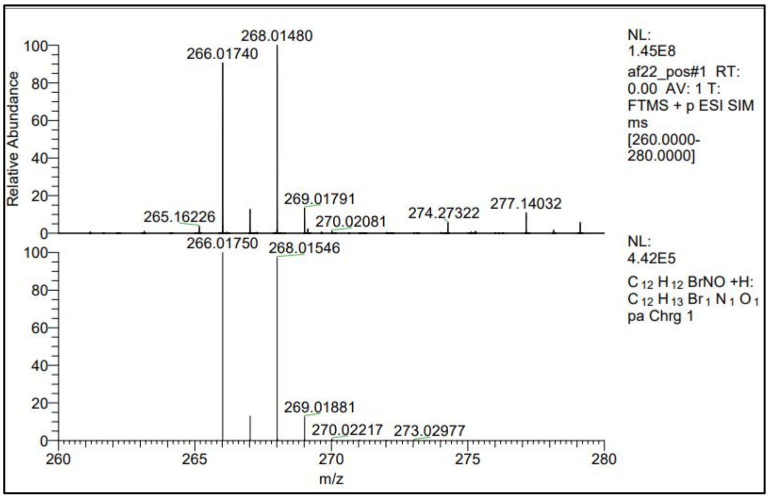

Figure S92. HMRS-ESI of compoud (*E*)-*N*-allyl-3-(4-bromophenyl)acrylamide **(AF14)**

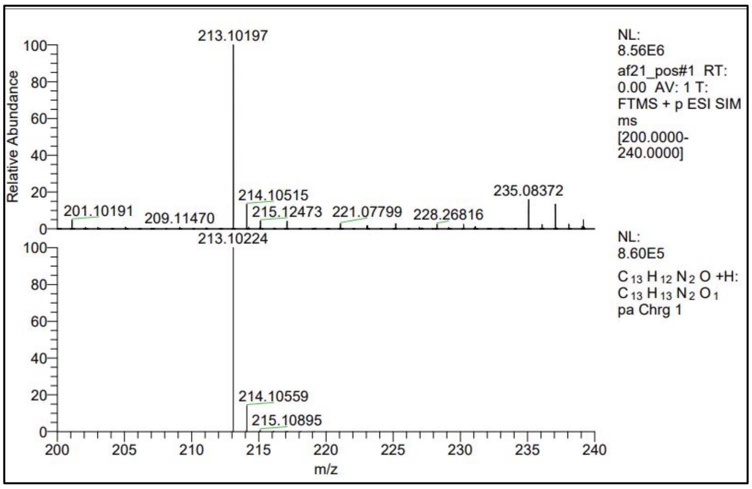

Figure S93. HRMS-ESI of compoud (*E*)-*N*-allyl-3-(4-cyanophenyl)acrylamide **(AF15)**

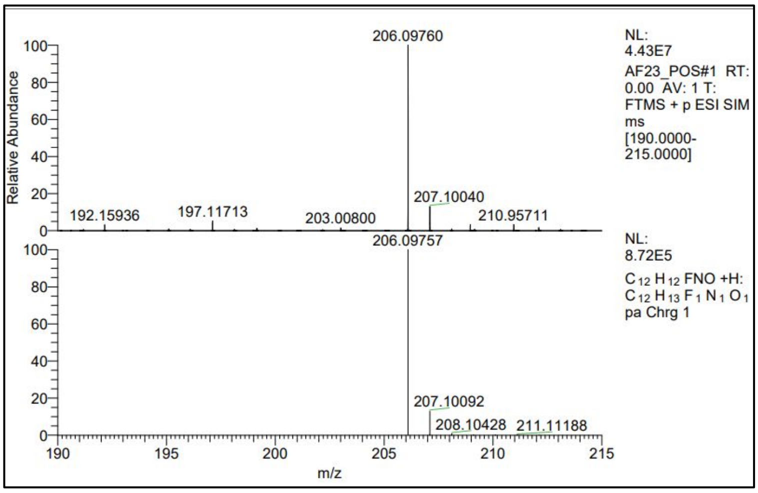

Figure S94. HRMS-ESI of compoud (*E*)-*N*-allyl-3-(4-fluorophenyl)acrylamide **(AF16)**

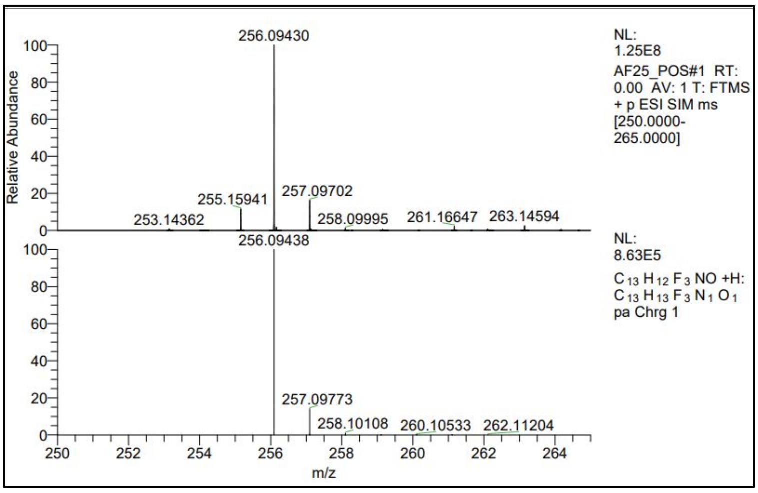

Figure S95. HRMS-ESI of compoud (*E*)-*N*-allyl-3-(4-(trifluoromethyl)phenyl)acrylamide **(AF17)**

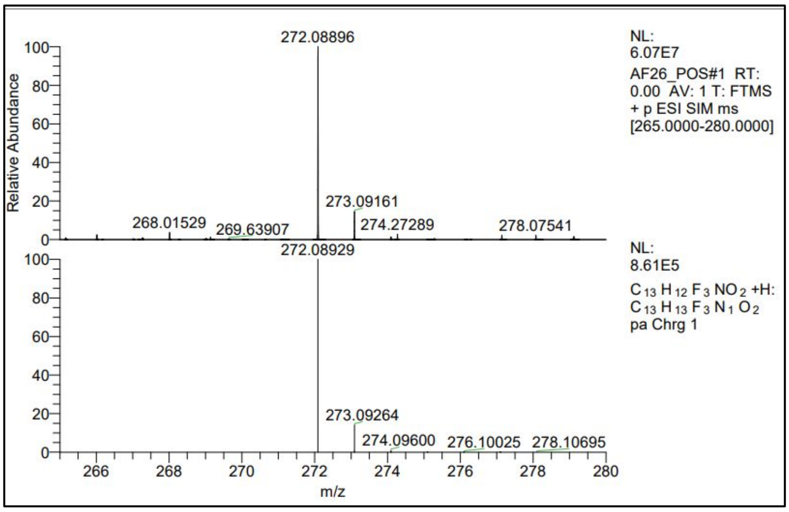

Figure S96. HRMS-ESI of compoud (*E*)-*N*-allyl-3-(4-(trifluoromethoxy)phenyl)acrylamide **(AF18)**

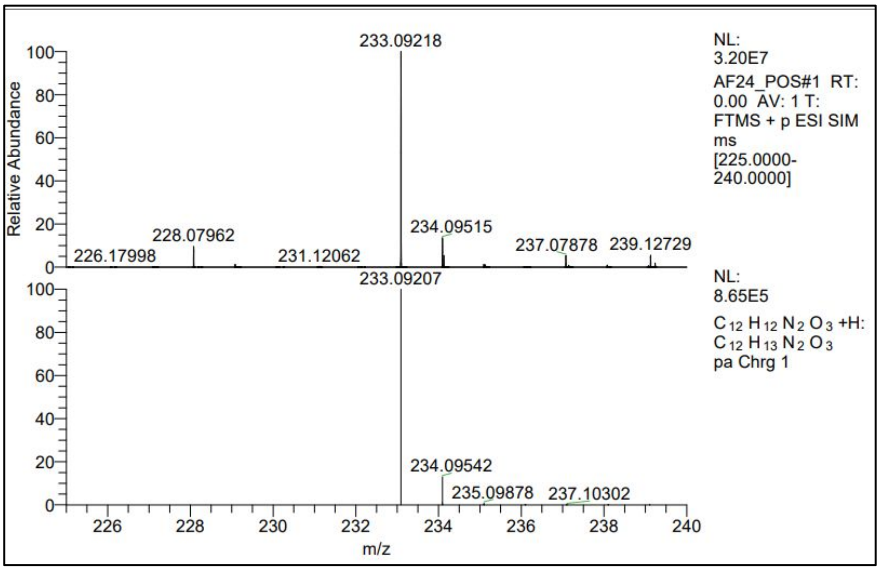

Figure S97. HMRS-ESI of compoud (*E*)-*N*-allyl-3-(4-nitrophenyl)acrylamide (**AF19**)

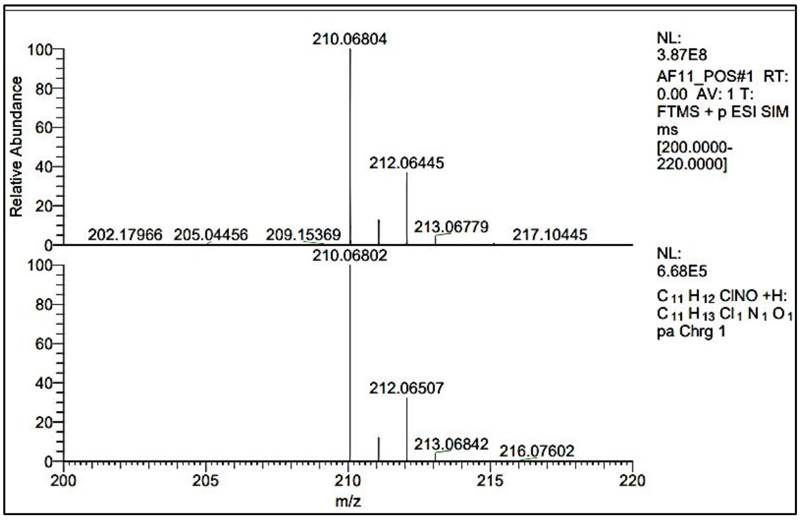

Figure S98. HMRS-ESI (*E*)-3-(4-chlorophenyl)-*N*-ethylacrylamide (**AF20**)

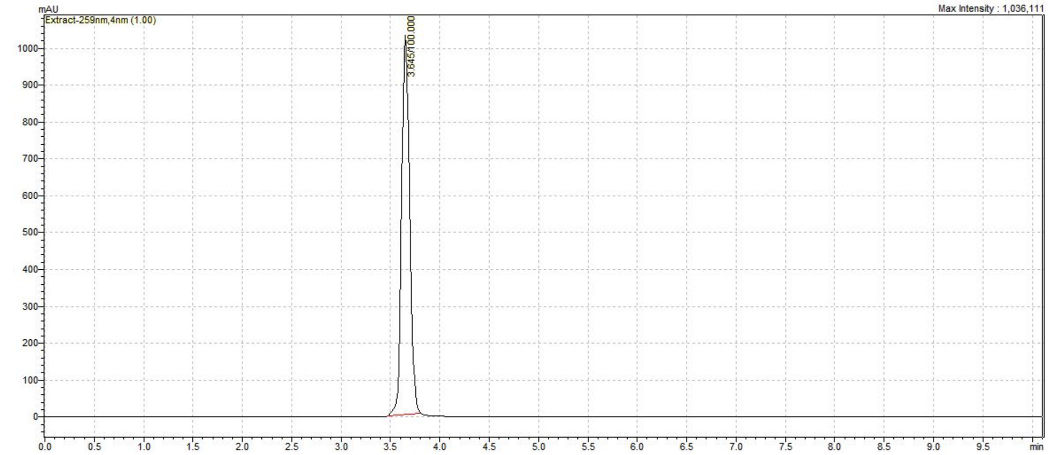

Figure S99. HPLC analysis demonstrating the purity of the compound (*E*)-3-(4-chlorophenyl)-*N*-hexylacrylamide **(AF01)**

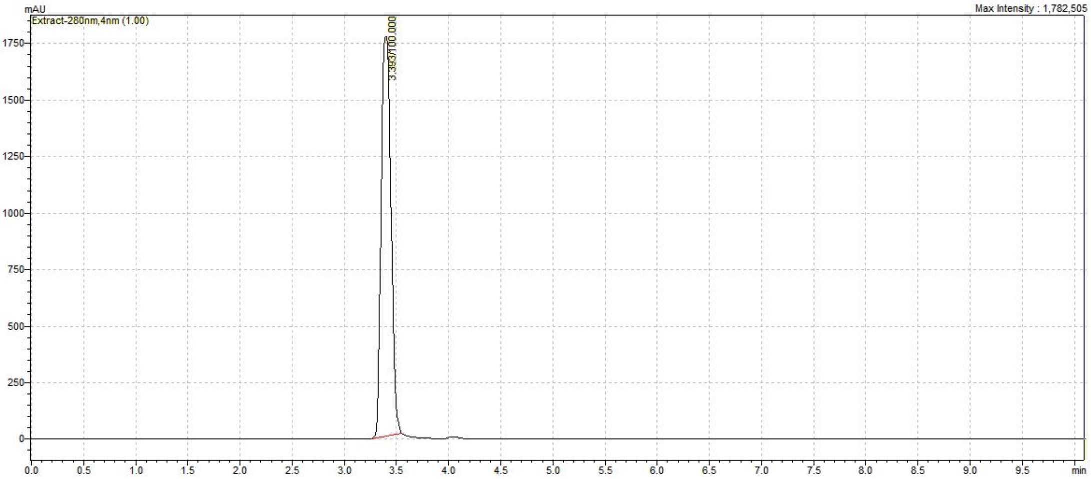

Figure S100. HPLC analysis demonstrating the purity of the compound (*E*)-3-(4-chlorophenyl)-*N*-phenethylacrylamide **(AF02)**

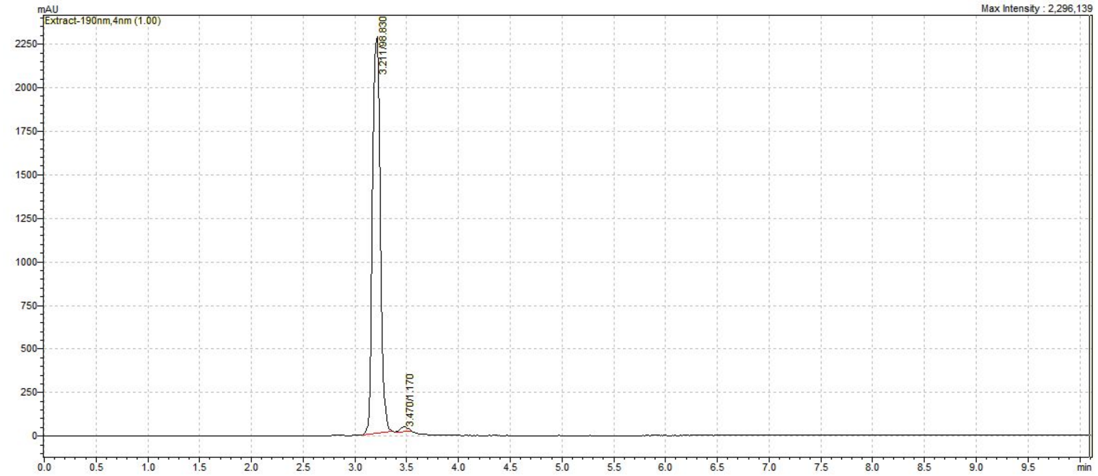

Figure S101. HPLC analysis demonstrating the purity of the compound (*E*)-*N*-allyl-3-(4-chlorophenyl)acrylamide (**AF03**)

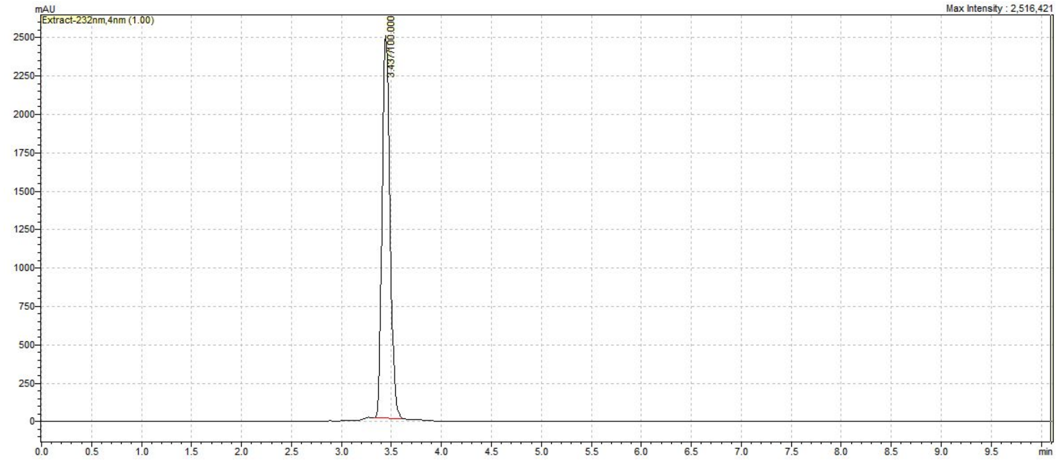

Figure S102. HPLC analysis demonstrating the purity of the compound *N*-hexylcinnamamide **(AF04)**

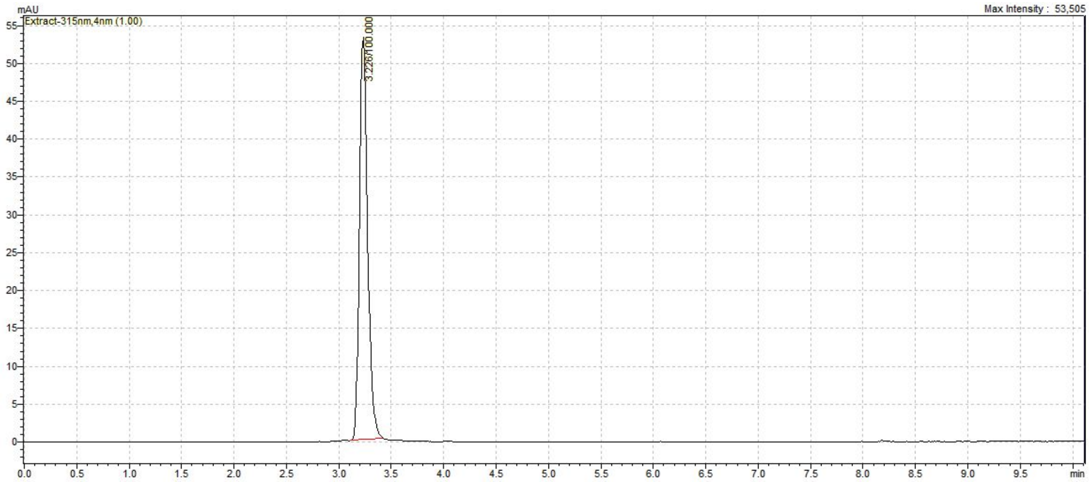

Figure S103. HPLC analysis demonstrating the purity of the compound (*E*)-*N*-phenethylcinnamamide **(AF05)**

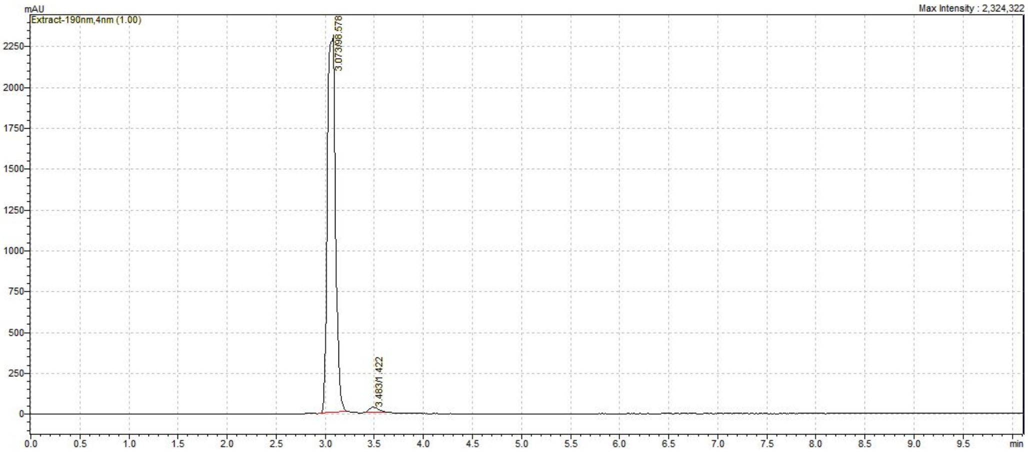

Figure S104. HPLC analysis demonstrating the purity of the compound *N*-allylcinnamamide (**AF06**)

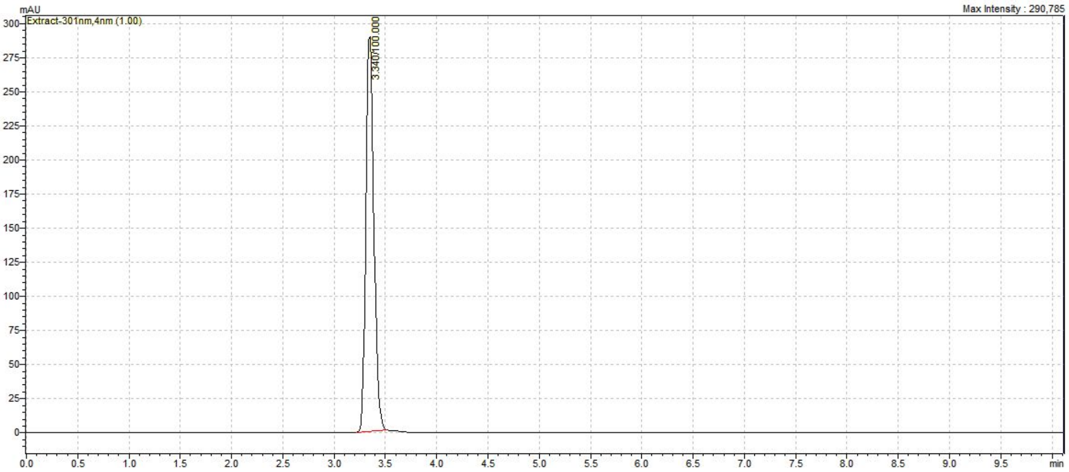

Figure S105. HPLC analysis demonstrating the purity of the compound (*E*)-3-(4-chlorophenyl)-*N*-(4-fluorophenethyl)acrylamide (**AF07**)

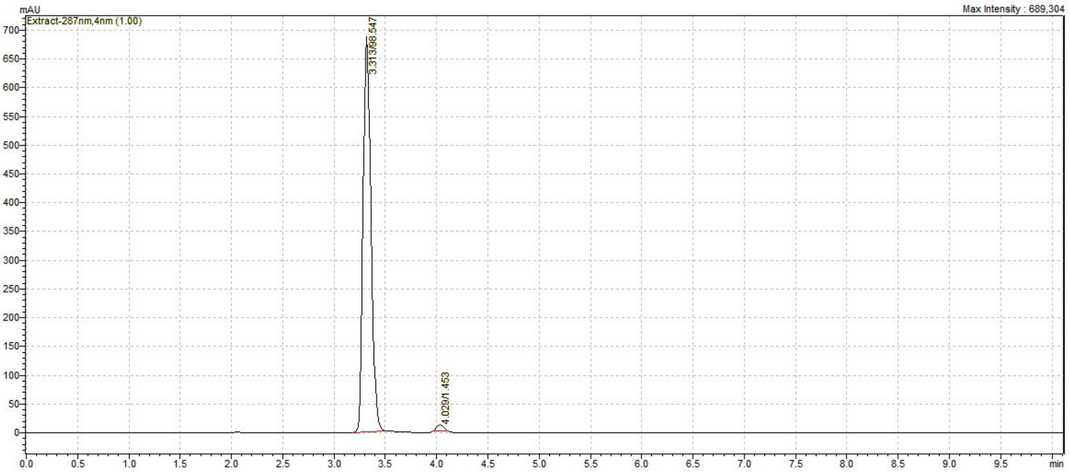

Figure S106. HPLC analysis demonstrating the purity of the compound (*E*)-*N*-benzyl-3-(4-chlorophenyl)acrylamide (**AF08**)

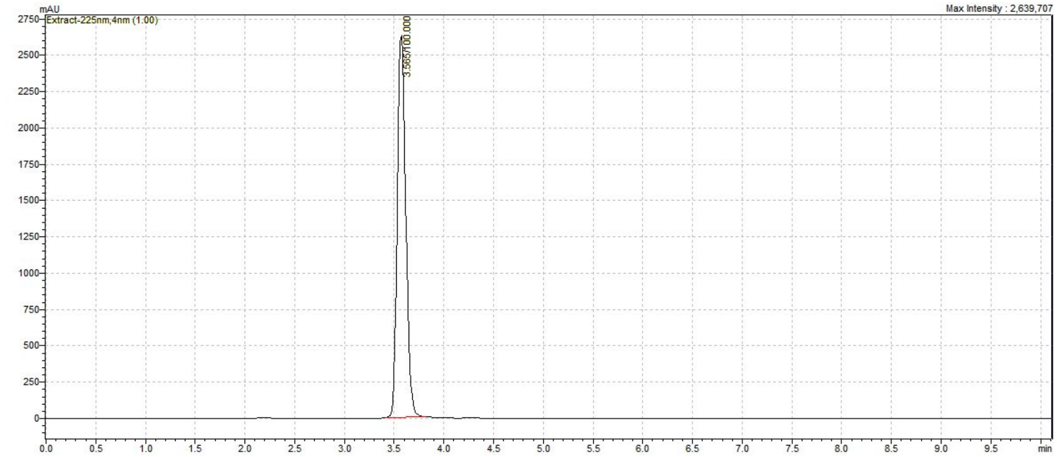

Figure S107. HPLC analysis demonstrating the purity of the compound (*E*)-3-(4-chlorophenyl)-*N*-cyclohexylacrylamide (**AF09**)

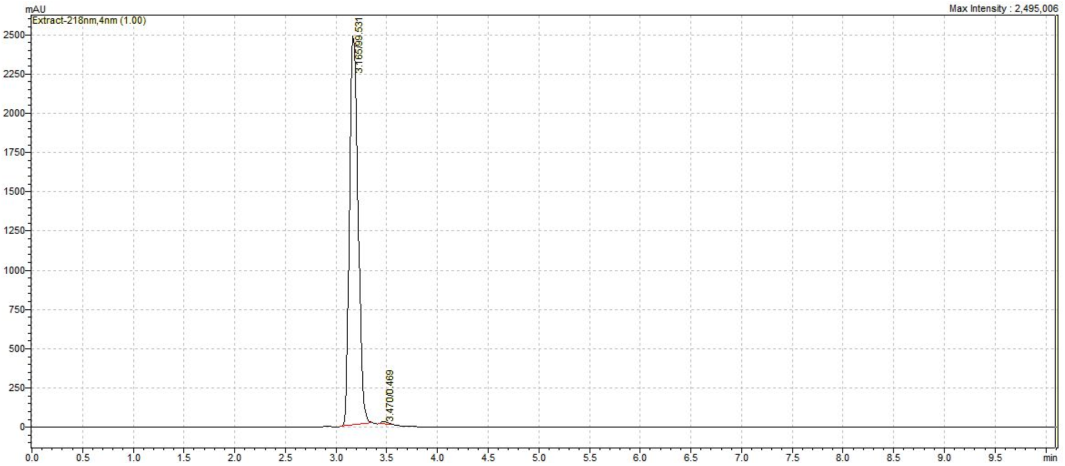

Figure S108. HPLC analysis demonstrating the purity of the compound *N*-(4-fluorophenethyl)cinnamamide (**AF10**)

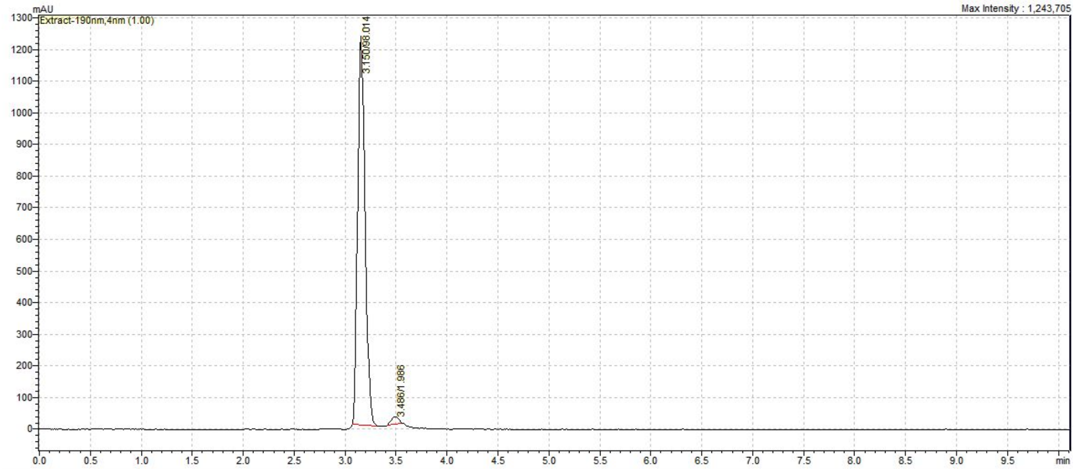

Figure S109. HPLC analysis demonstrating the purity of the compound *N*-Benzylcinnamamide **(AF11)**

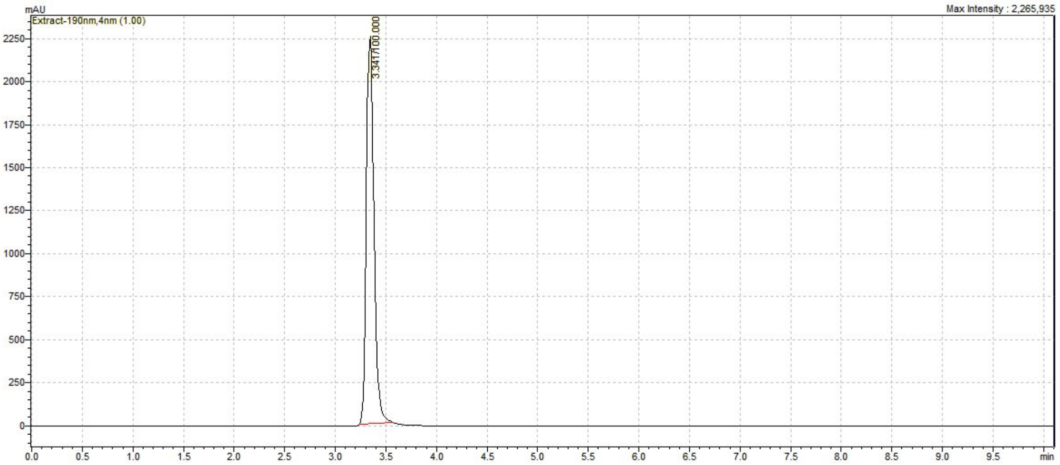

Figure S110. HPLC analysis demonstrating the purity of the compound *N*-cyclohexylcinnamamide (**AF12**)

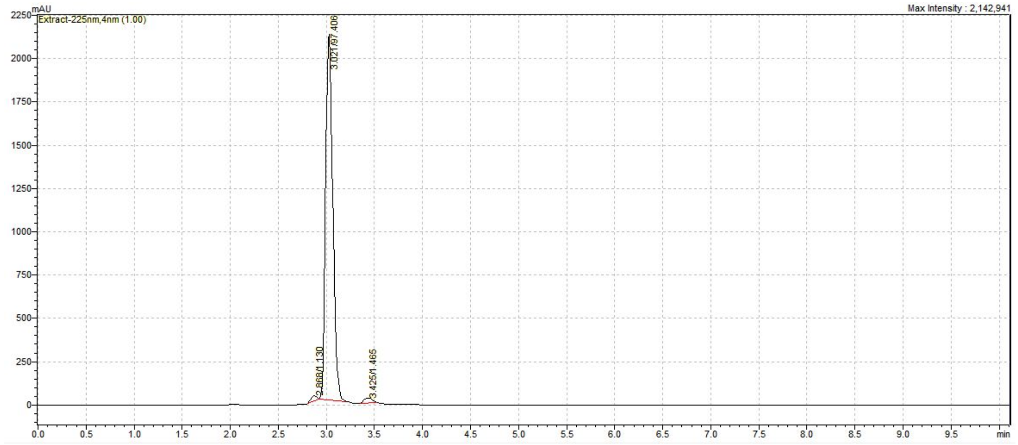

Figure S111. HPLC analysis demonstrating the purity of the compound (*E*)-*N*-allyl-3-(4-methoxyphenyl)acrylamide **(AF13)**

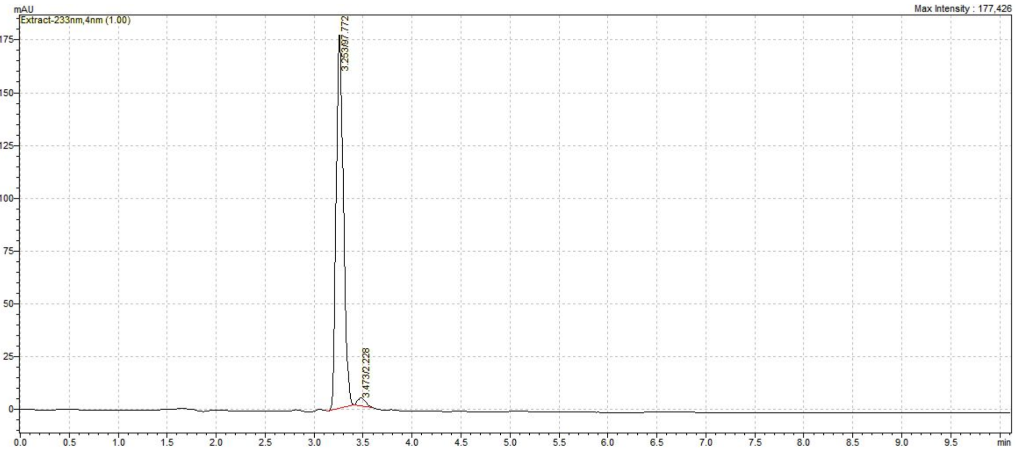

Figure S112. HPLC analysis demonstrating the purity of the compound (*E*)-*N*-allyl-3-(4-bromophenyl)acrylamide **(AF14)**

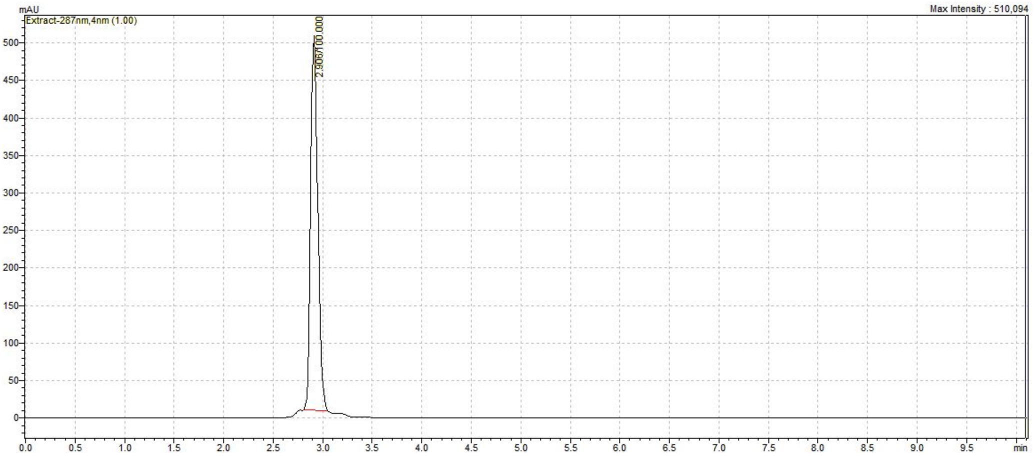

Figure S113. HPLC analysis demonstrating the purity of the compound (*E*)-*N*-allyl-3-(4-cyanophenyl)acrylamide **(AF15)**

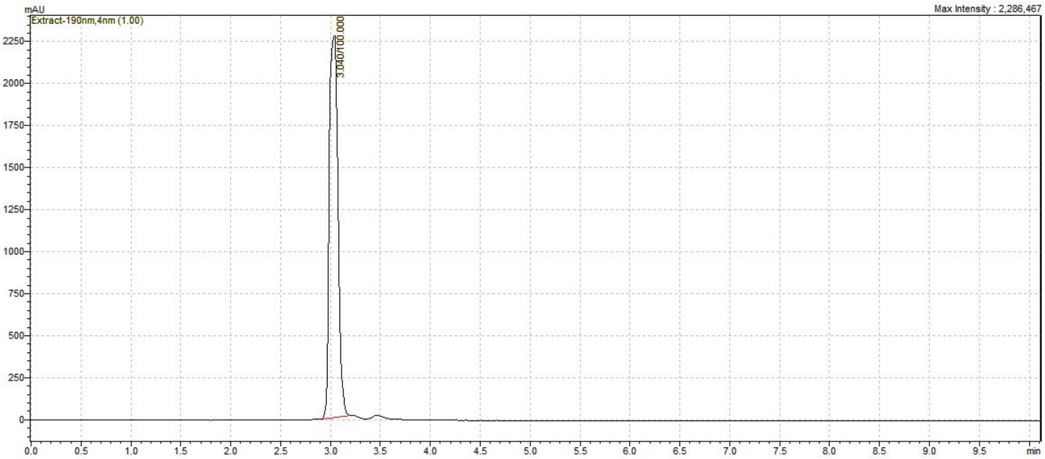

Figure S114. HPLC analysis demonstrating the purity of the compound (*E*)-*N*-allyl-3-(4-fluorophenyl)acrylamide **(AF16)**

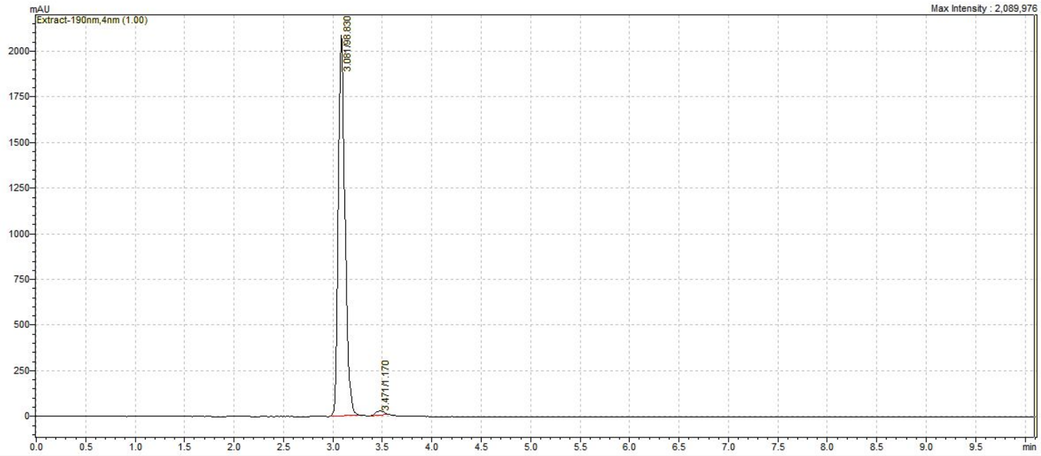

Figure S115. HPLC analysis demonstrating the purity of the compound (*E*)-*N*-allyl-3-(4-(trifluoromethyl)phenyl)acrylamide **(AF17)**

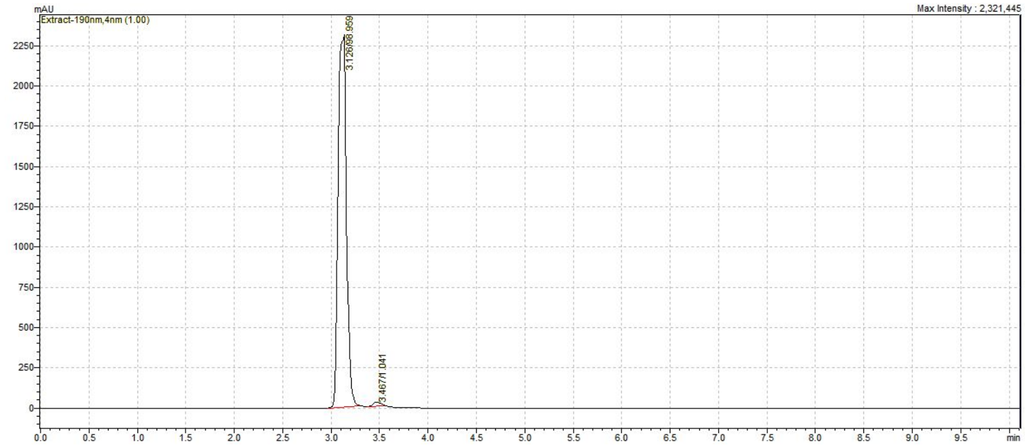

Figure S116. HPLC analysis demonstrating the purity of the compound (*E*)-*N*-allyl-3-(4-(trifluoromethoxy)phenyl)acrylamide **(AF18)**

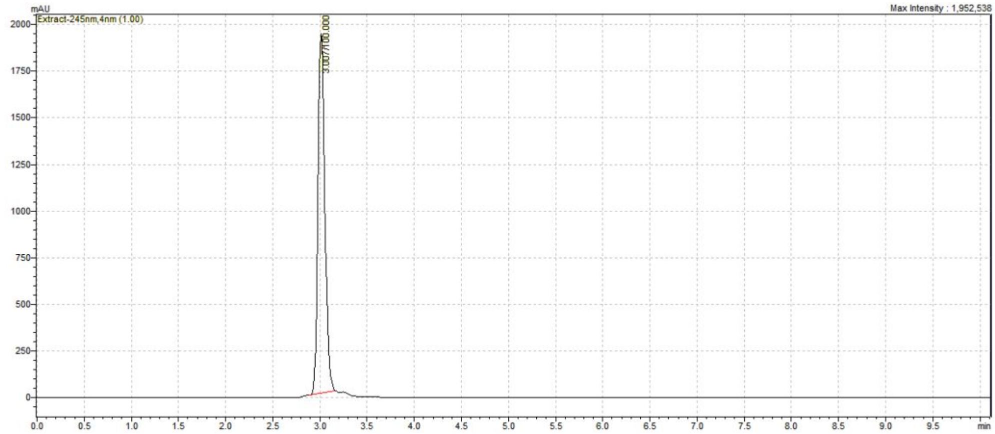

Figure S117. HPLC analysis demonstrating the purity of the compound (*E*)-*N*-allyl-3-(4-nitrophenyl)acrylamide (**AF19**)

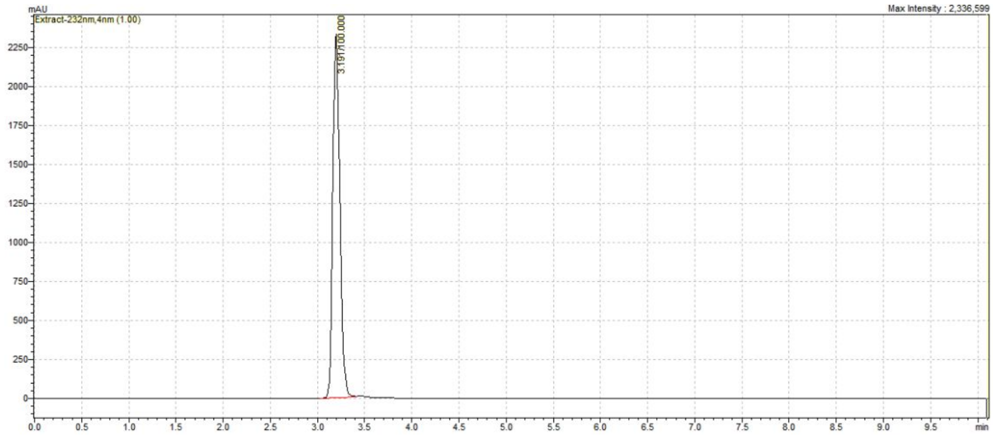

Figure S118. HPLC analysis demonstrating the purity of the compound (*E*)-3-(4-chlorophenyl)-*N*-ethylacrylamide (**AF20**)

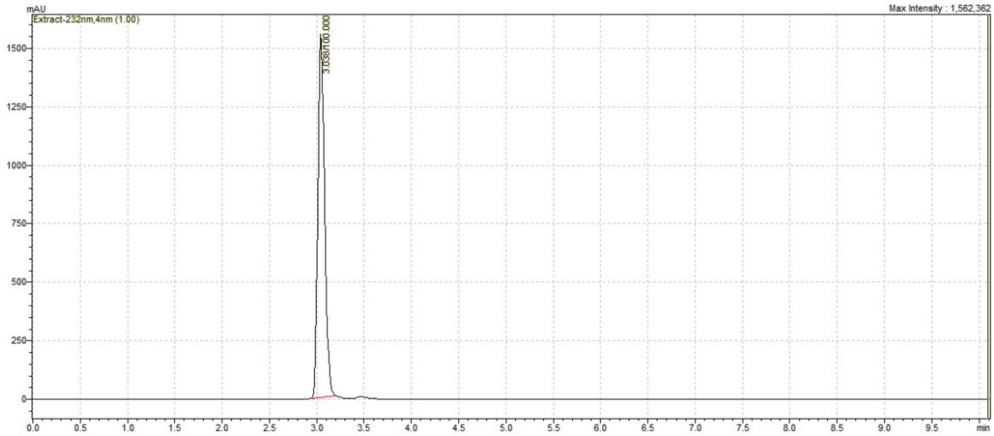

Figure S119. HPLC analysis demonstrating the purity of the compound Ethylcinnamamide (**AF21**)
